# Day-and-night glycaemic control with closed-loop insulin delivery versus conventional insulin pump therapy in free-living adults with well controlled type 1 diabetes: an open-label, randomised, crossover study

**DOI:** 10.1016/S2213-8587(17)30001-3

**Published:** 2017-04

**Authors:** Lia Bally, Hood Thabit, Harald Kojzar, Julia K Mader, Jehona Qerimi-Hyseni, Sara Hartnell, Martin Tauschmann, Janet M Allen, Malgorzata E Wilinska, Thomas R Pieber, Mark L Evans, Roman Hovorka

**Affiliations:** a Wellcome Trust–Medical Research Council Institute of Metabolic Science, University of Cambridge, Cambridge, UK; b Department of Paediatrics, University of Cambridge, Cambridge, UK; c Department of Diabetes & Endocrinology, Cambridge University Hospitals National Health Service Foundation Trust, Cambridge, UK; d Department of Diabetes, Endocrinology, Clinical Nutrition & Metabolism, Inselspital, Bern University Hospital, University of Bern, Bern, Switzerland; e Department of Internal Medicine, Division of Endocrinology & Diabetology, Medical University of Graz, Graz, Austria

## Abstract

**Background:**

Tight control of blood glucose concentration in people with type 1 diabetes predisposes to hypoglycaemia. We aimed to investigate whether day-and-night hybrid closed-loop insulin delivery can improve glucose control while alleviating the risk of hypoglycaemia in adults with HbA_1c_ below 7·5% (58 mmol/mol).

**Methods:**

In this open-label, randomised, crossover study, we recruited adults (aged ≥18 years) with type 1 diabetes and HbA_1c_ below 7·5% from Addenbrooke's Hospital (Cambridge, UK) and Medical University of Graz (Graz, Austria). After a 2–4 week run-in period, participants were randomly assigned (1:1), using web-based randomly permuted blocks of four, to receive insulin via the day-and-night hybrid closed-loop system or usual pump therapy for 4 weeks, followed by a 2–4 week washout period and then the other intervention for 4 weeks. Treatment interventions were unsupervised and done under free-living conditions. During the closed-loop period, a model-predictive control algorithm directed insulin delivery, and prandial insulin delivery was calculated with a standard bolus wizard. The primary outcome was the proportion of time when sensor glucose concentration was in target range (3·9–10·0 mmol/L) over the 4 week study period. Analyses were by intention to treat. This study is registered with ClinicalTrials.gov, number NCT02727231, and is completed.

**Findings:**

Between March 21 and June 24, 2016, we recruited 31 participants, of whom 29 were randomised. One participant withdrew during the first closed-loop period because of dissatisfaction with study devices and glucose control. The proportion of time when sensor glucose concentration was in target range was 10·5 percentage points higher (95% CI 7·6–13·4; p<0·0001) during closed-loop delivery compared with usual pump therapy (65·6% [SD 8·1] when participants used usual pump therapy *vs* 76·2% [6·4] when they used closed-loop). Compared with usual pump therapy, closed-loop delivery also reduced the proportion of time spent in hypoglycaemia: the proportion of time with glucose concentration below 3·5 mmol/L was reduced by 65% (53–74, p<0·0001) and below 2·8 mmol/L by 76% (59–86, p<0·0001). No episodes of serious hypoglycaemia or other serious adverse events occurred.

**Interpretation:**

Use of day-and-night hybrid closed-loop insulin delivery under unsupervised, free-living conditions for 4 weeks in adults with type 1 diabetes and HbA_1c_ below 7·5% is safe and well tolerated, improves glucose control, and reduces hypoglycaemia burden. Larger and longer studies are warranted.

**Funding:**

Swiss National Science Foundation (P1BEP3_165297), JDRF, UK National Institute for Health Research Cambridge Biomedical Research Centre, and Wellcome Strategic Award (100574/Z/12/Z).

## Introduction

Intensive insulin therapy is the standard of care in the management of type 1 diabetes.^1^ Although modern insulin therapy has led to a reduction in the frequency of severe hypoglycaemic events,^2^ tight glycaemic control remains a predisposing factor to hypoglycaemia and its effect is amplified by duration of the disease.^3^ Recurrent exposure to hypoglycaemia might lead to attenuated counter-regulatory response to subsequent hypoglycaemic events and, ultimately, impaired hypoglycaemia awareness.^4^ Frequent hypoglycaemic episodes might have a profound effect on behaviour and diabetes self-management, adversely affecting quality of life.^5^

The advent of continuous glucose monitoring (CGM) has led to improved glycaemic control and reduced exposure to hypoglycaemia, including severe hypoglycaemia.^6^, ^7^ The benefits of hypoglycaemia reduction are enhanced in hypoglycaemia-prone individuals when CGM is integrated with the threshold suspend feature of insulin pumps, which allows insulin delivery to be suspended automatically for up to 2 h when the pre-set glucose threshold is reached^8^ or predicted.^9^ Although these technologies have been shown to reduce the incidence of severe hypoglycaemic events, including those leading to hypoglycaemic seizure or coma,^10^ they do not address the issue of variability in insulin requirements,^11^ which remains an unmet need in patients with type 1 diabetes.

**Research in context**

**Evidence before this study**

We searched PubMed from database inception until Oct 24, 2016, using the search terms “type 1 diabetes” AND (“artificial pancreas” OR “closed-loop”) AND (“home” OR “outpatient”), for reports of randomised controlled trials published in English only. We identified 14 randomised trials assessing the use of closed-loop insulin delivery outside hospital settings. In two randomised home studies in participants with mean HbA_1c_ above 7·5% (58 mmol/mol), long-term (>4 week) use of closed-loop insulin delivery led to a significant decrease in HbA_1c_ and improvement in mean glucose and proportion of time spent within, below, and above the glucose target range (3·9–10·0 mmol/L). However, no studies have so far assessed closed-loop use in non-pregnant adults with HbA_1c_ below 7·5%.

**Added value of this study**

To our knowledge, this study is the first randomised controlled study in free-living adults with type 1 diabetes whose HbA_1c_ is below 7·5%. We showed that, compared with usual insulin pump therapy, day-and-night closed-loop insulin delivery significantly improved overall glycaemic control while reducing the burden of hypoglycaemia. Beneficial effects on glycaemic outcomes included increased time with glucose concentration in target range, reduced time with glucose concentration below and above target range, and decreased mean glucose concentration and glycaemic variability. The findings of increased time in target range and reduced overall hypoglycaemia risks and sensor glucose variability were similarly observed and consistent during night-time and daytime periods of closed-loop use. These outcomes were achieved without change in total insulin delivered. Closed-loop application thereby provides a novel therapeutic approach to optimise glycaemic control in hypoglycaemia-prone adults with HbA_1c_ below 7·5%. Closed-loop application was well tolerated in this population with advanced self-management skills, and might provide clinically significant benefits to their overall diabetes care.

**Implications of all the available evidence**

The use of day-and-night closed-loop insulin delivery might further improve glycaemic control while reducing the risk and burden of hypoglycaemia in adults with type 1 diabetes whose HbA_1c_ is below 7·5%. Results from our study, together with those from previous studies in different target groups, support the benefits of closed-loop insulin delivery in a broad population with type 1 diabetes.

Closed-loop insulin delivery—also known as the artificial pancreas—is a therapeutic approach that is progressing quickly. Closed-loop delivery differs from conventional pump therapy and threshold suspend technology; it has a control algorithm that autonomously increases and decreases subcutaneous insulin delivery in response to real-time sensor glucose levels.^12^ Results from randomised trials^13^, ^14^, ^15^, ^16^ of day-and-night closed-loop use during unsupervised free-living conditions in children, adolescents, and adults have shown improved glycaemic outcomes, reduced risk of non-severe hypoglycaemic events, and positive user experience. However, outside of pregnancy,^15^ none of the studies have focused specifically on patients with well controlled diabetes (HbA_1c_ <7·5% [58 mmol/mol]) who might have a heightened, but masked, risk of hypoglycaemia and glucose variability. In this study, we aimed to investigate whether day-and-night hybrid closed-loop insulin delivery—in which manual administration of prandial bolus was implemented by the user—under free-living conditions in adults with HbA_1c_ below 7·5% can improve glucose control while alleviating the risk of hypoglycaemia, thus informing whether the use and reimbursement of closed-loop systems is justified in this particular population.

## Methods

### Study design and participants

In this open-label, randomised, crossover study, we recruited adults (aged ≥18 years) attending diabetes clinics at Addenbrooke's Hospital (Cambridge, UK) and Medical University of Graz (Graz, Austria). Patients were eligible if they had type 1 diabetes (defined according to WHO criteria), non-hypoglycaemic C-peptide concentration less than 100 pmol/L, and HbA_1c_ less than 7·5%; had been using insulin pump therapy for at least 6 months; had knowledge of insulin self-adjustment; and had been self-monitoring their blood glucose concentration at least six times per day. Exclusion criteria included established nephropathy, neuropathy, or proliferative retinopathy; total daily insulin dose of 2·0 U/kg or more; hypoglycaemia unawareness (determined by Gold score ≥4 on the basis of pre-study clinical records); severe visual or hearing impairment; pregnancy; or breastfeeding (see appendix for the full list of inclusion and exclusion criteria).

The study was approved by the local ethics committees and national competent authorities in the UK and Austria, and the protocol (phase 2 of APhome04 study) is shown in the appendix. All participants provided written informed consent before the start of study-related procedures.

### Randomisation and masking

Participants were randomly assigned (1:1) to receive either day-and-night closed-loop insulin delivery followed by usual pump therapy with blinded CGM, or vice versa. Following the run-in period, the order of the two study periods was randomly determined with an automated web-based programme with locally stratified, randomly permuted blocks of four. Participants and investigators analysing study data were not masked to treatment allocation.

### Procedures

After screening, all participants underwent a 2–4 week run-in period, during which they were trained to use the study insulin pump and CGM device, and calibrated the real-time CGM device according to manufacturer's instructions. Compliance assessment after the run-in period was based on at least 10 days of CGM use in the past 2 weeks.

Participants then received insulin via the day-and-night closed-loop system (closed-loop period) for 4 weeks and via usual pump therapy with blinded CGM (control period) for 4 weeks, in the order assigned at randomisation, with a 2–4 week washout period in between. During the washout period, participants returned to their usual care and did not use the study CGM device. Identical study insulin pumps and CGM devices were used during the two treatment periods. Participants used rapid-acting insulin analogue normally applied in their usual clinical care. The built-in bolus wizard of the study insulin pump was used by participants during both treatment periods to calculate insulin boluses at mealtimes and when administering correction boluses. The study was done under free-living conditions without direct or remote supervision by clinical investigators. Participants were not restricted in their dietary intake or daily activities. Support was available at all times to assist participants in case of clinical or technical issues that arose during the study. Standard local hypoglycaemia and hyperglycaemia treatment guidelines were followed.

The FlorenceD2A closed-loop system (University of Cambridge, Cambridge, UK) comprised a model-predictive control algorithm on a smartphone (Galaxy S4, Samsung, South Korea), which communicated wirelessly with a purpose-made translator unit (Triteq, Hungerford, UK) and the study pump (DANA-R Diabecare, Sooil, Seoul, South Korea) through a Bluetooth communication protocol (appendix). The CGM receiver (FreeStyle Navigator II, Abbott Diabetes Care, Alameda, CA, USA) was inserted into the translator, which translated a serial USB protocol into a Bluetooth communication protocol. The calculations used a compartment model of glucose kinetics^17^ describing the effect of rapid-acting insulin and the carbohydrate content of meals on glucose levels. We applied a hybrid closed-loop approach in which participants were required to count carbohydrates and use a standard bolus calculator for pre-meal boluses according to usual practice. The bolus calculations provided by the study pump's built-in bolus calculator took into account carbohydrate content of meals, insulin on board, and entered capillary blood glucose readings. The algorithm was initialised by pre-programmed basal insulin rates downloaded from the study pump. Participants' bodyweight and total daily insulin dose were entered at set-up. During closed-loop operation, the algorithm adapted itself to a particular participant. The treat-to-target control algorithm aimed to achieve glucose concentrations of 5·8–7·3 mmol/L, and adjusted the actual concentration depending on fasting versus postprandial status and the accuracy of model-based glucose predictions. Control Algorithm version 0.3.46 was used (University of Cambridge, Cambridge, UK) which, compared with that used in our previous study,^13^ included enhanced adaptation of insulin needs based on analysis of past performance and identification of the time of day when insulin needs are consistently lower or higher. Participants were trained to perform a calibration check before breakfast and evening meal. If sensor glucose was above capillary glucose by more than 3 mmol/L, participants were advised to recalibrate the CGM device. These instructions resulted from in-silico assessment of hypoglycaemia and hyperglycaemia risks using the validated Cambridge simulator.^18^ Safety rules limited maximum insulin infusion and suspended insulin delivery at sensor glucose at or less than 4·3 mmol/L, or when sensor glucose was rapidly decreasing.

During the control period, the display of the study CGM device was masked. Participants were allowed to use their own glucose monitoring devices (CGM or flash glucose monitoring^19^) if they were part of their pre-study usual care. The control intervention was chosen according to existing clinical practice and participants' preferences.^20^ The rationale for the control period was to reflect usual clinical practice and national reimbursement policies, and to compare the incremental benefits gained by closed-loop insulin delivery with the therapeutic modality followed by the participants in a pragmatic study design.

At the start of the closed-loop period, participants were admitted to the local clinical research facility for a training session, which covered starting and stopping of the closed-loop system and troubleshooting of technical issues. If sensor glucose readings became unavailable, or in case of other system failures, participants were alerted by an audible alarm and the system restarted the participant's usual insulin delivery rate within 30–60 min to mitigate the risk of insulin under-delivery and over-delivery.^21^ Participants were instructed to have the low-glucose alarm audible at all times. The sensor glucose alarm threshold for hypoglycaemia was initially set at 3·5 mmol/L, but these settings could be later modified by the participants. A user feedback questionnaire was completed by participants at the end of the closed-loop period.

Blood samples for HbA_1c_ and C-peptide measurements were taken after enrolment. Plasma C-peptide was measured with chemiluminescence immunoassays (Diasorin Liaison XL [Deutschland GmbH, Dietzenbach, Germany] used in Cambridge; ADVIA Centaur [Siemens Healthcare Diagnostics, USA] used in Graz). HbA_1c_ was measured with ion exchange high-performance liquid chromatography, compliant with the International Federation of Clinical Chemistry and Laboratory Medicine, at study centres (G8 HPLC Analyzer [Tosoh Bioscience, South San Francisco, CA, USA] in Cambridge; Menarini HA-8160 HbA_1c_ auto-analyser [Menarini Diagnostics, Florence, Italy] in Graz).

### Outcomes

The primary outcome was the proportion of time during the whole study when sensor glucose concentration was in the target range of 3·9–10·0 mmol/L. Secondary efficacy outcomes were proportion of time with sensor glucose concentration above and below target range; time with glucose concentration below 3·5 mmol/L, 3·3 mmol/L (post hoc), and 2·8 mmol/L, and above 13·9 mmol/L (post hoc) and 16·7 mmol/L; the number of nights and mean duration when sensor glucose was below 3·5 mmol/L for at least 20 min; hypoglycaemia burden (ie, area under the curve when sensor glucose concentration was less than 3·5 mmol/L; post hoc); mean, SD, and coefficient of variation (post hoc) of sensor glucose; total daily, basal, and bolus insulin dose; and weekly trends in glucose control and insulin delivery. The between-day coefficient of variation of sensor glucose was calculated from daily mean glucose values (midnight to midnight). To limit multiple comparison, daytime (0601 h to 2359 h) and night-time (0000 h to 0600 h) endpoints were calculated with data from the respective periods for a subset of outcomes—proportion of time with glucose concentration in target range, above and below target range, below 3·5 mmol/L, and below 2·8 mmol/L; SD of sensor glucose concentration; between-day and between-night coefficient of variation of sensor glucose concentration; and area under the curve when sensor glucose concentration was less than 3·5 mmol/L.

Of note, two prespecified secondary outcomes will not be reported. We will not report time spent in target glucose range (3·9–10·0 mmol/L) based on subcutaneous glucose monitoring adjusted for sensor error during the entire home stay because the general consensus is that outcomes based on unadjusted CGM values should be reported.^22^ Additionally, low blood glucose index will not be reported since it is not generally well understood by non-specialists and is highly correlated with time with glucose concentration below 3·9 mmol/L.^23^

Safety outcomes were severe hypoglycaemic events, significant ketonaemia (>3·0 mmol/L), and other adverse and serious adverse events. We also assessed the frequency and duration of use of the closed-loop system at home as a measure of utility outcome.

### Statistical analysis

The power calculation was based on improvements in time spent in glucose concentration target range. Assuming an SD of 18% and mean improvement of time spent in target range of 10%,^13^, ^24^ 31 participants were needed at the desired 80% power and α level of 0·05 (two-tailed). If the mean improvement was 12%, the required sample size was reduced to 20. We planned to recruit up to 34 participants, aiming for 24 participants to complete the study to allow for dropouts (anticipated dropout rate of 25% based on the investigators' experience and expectation). Participants who dropped out of the study during the run-in period and within the first 2 weeks of the first treatment period were allowed to be replaced.

We agreed on the statistical analysis plan following completion of the last patient's last visit but before the final dataset was reviewed and analysed. The analyses were done by intention to treat. Efficacy and safety data from all randomised participants, including those who dropped out, were included in the analysis. We compared the respective measurements obtained during the closed-loop period and the control period using a regression model that accounts for period effect. Log-transformed analyses were used for highly skewed endpoints. Values were presented as mean (SD) or median (IQR) for each study period. A 5% significance level was used to declare statistical significance for all comparisons. Outcomes were calculated with GStat software (University of Cambridge, Cambridge, UK), version 2.2.4, and statistical analyses were done with SPSS (IBM Software, Hampshire, UK), version 23.

This study is registered with ClinicalTrials.gov, number NCT02727231.

### Role of the funding source

The funders of the study had no role in the study design, data collection, data analysis, data interpretation, or writing of the report. Abbott Diabetes Care read the manuscript before submission. The corresponding author had full access to all the data in the study and had final responsibility for the decision to submit for publication.

## Results

**Figure 1:**
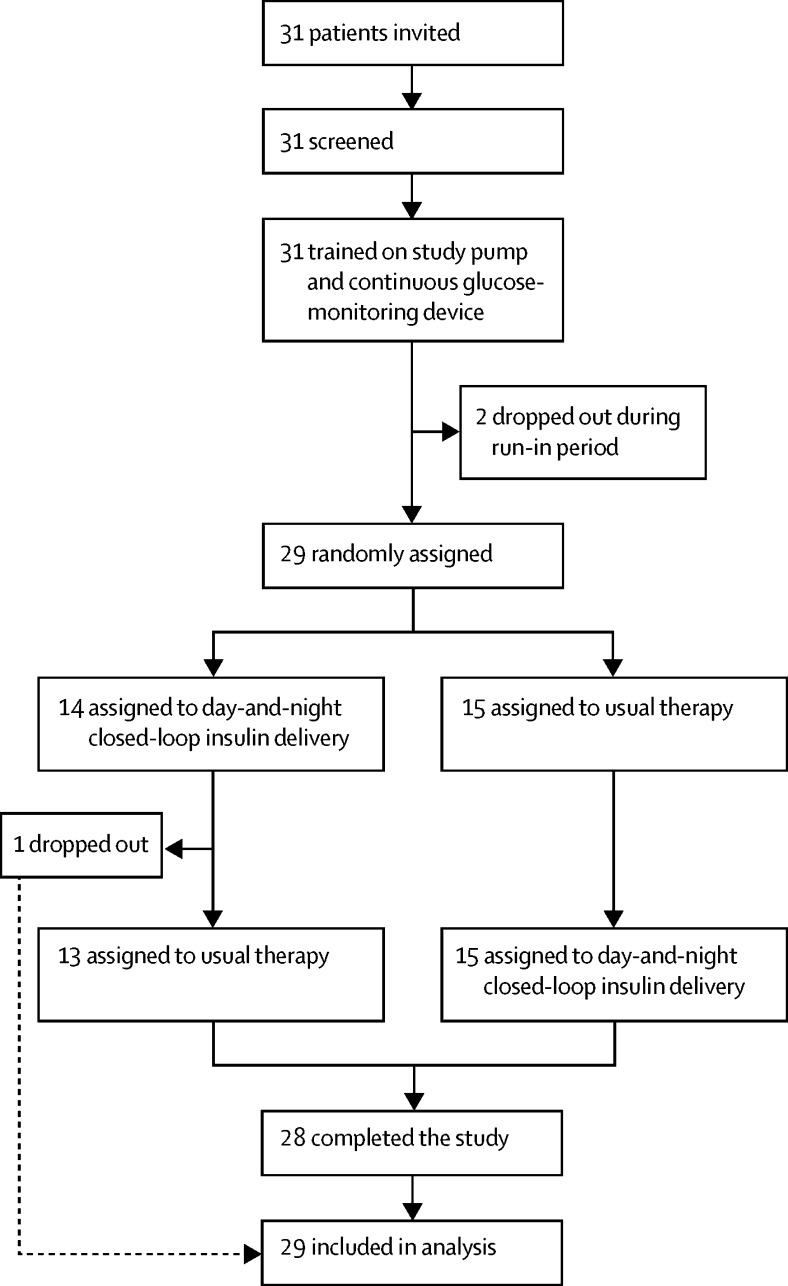
Trial profile

Between March 21 and June 24, 2016, we recruited 31 participants (figure 1). Two participants withdrew during the run-in period because of issues associated with use of the study pump. 29 eligible participants (17 from Cambridge and 12 from Graz) were randomly assigned. One participant dropped out during the first closed-loop period because of dissatisfaction with study devices and glycaemic control. Of 29 randomised participants, five (17%) used real-time CGM and six (21%) used flash glucose monitoring as part of their usual care (table 1; appendix).

**Figure 2:**
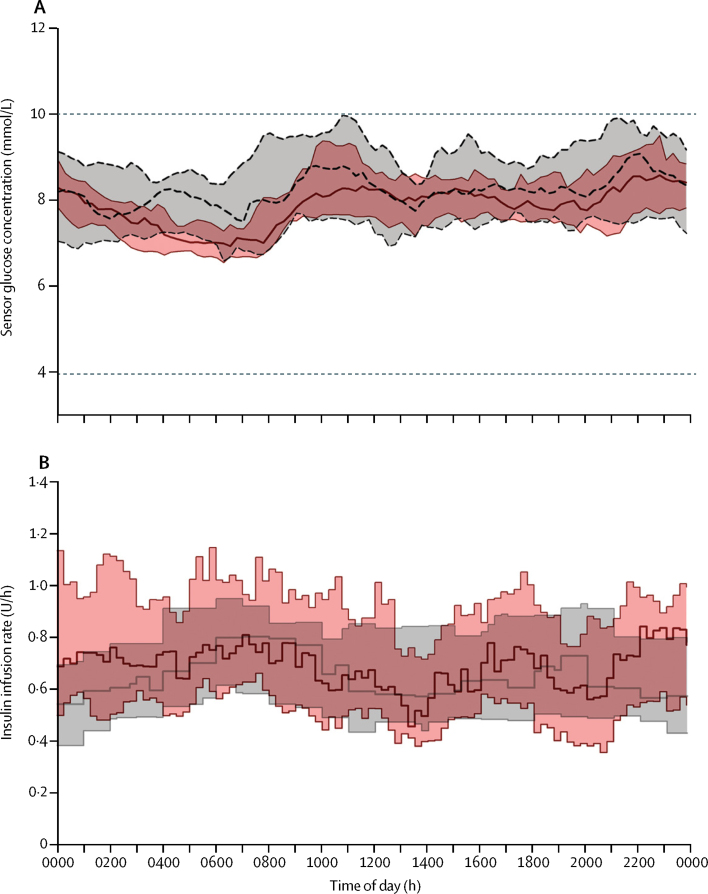
Median sensor glucose and insulin delivery for the 24 h duration over the study period Median (IQR) sensor glucose concentration (A) and insulin delivery (B) during closed-loop period (solid red line and red shaded area) and control period (dashed black line and grey shaded area) for the 24 h duration. The horizontal dashed lines show the lower and upper limits of the glucose target range (3·9–10·0 mmol/L).

The primary outcome of the study—the proportion of time during the whole study period when sensor glucose concentration was in the target range (3·9–10·0 mmol/L)—was 10·5 percentage points higher (95% CI 7·6–13·4; p<0·0001) during the closed-loop period than during the control period (65·6% [SD 8·1] when participants were using usual pump therapy *vs* 76·2% [6·4] when they used closed-loop; table 2). 24 h sensor glucose and insulin delivery profiles are shown in figure 2. The proportion of time sensor glucose concentration was in the target range seemed unchanged over the 4 week intervention periods (appendix).

**Figure 3:**
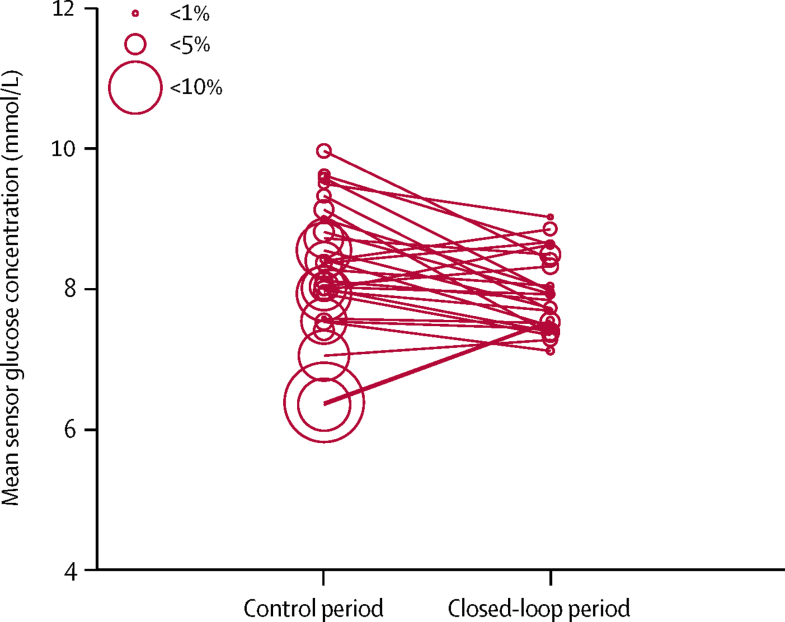
Individual values of mean sensor glucose and proportion of time spent with glucose concentration below the target range for the whole study The size of the circles denotes the proportion of time spent with low glucose (<3·5 mmol/L).

Day-and-night closed-loop insulin delivery reduced mean glucose concentration by 0·4 mmol/L (0·1–0·7, p=0·0226) compared with usual pump therapy (table 2, figure 3). Compared with the control period, day-and-night closed-loop insulin delivery reduced the proportion of time with glucose concentration below 3·9 mmol/L by 50% (37–59, p<0·0001), below 3·5 mmol/L by 65% (53–74, p<0·0001), below 3·3 mmol/L by 70% (57–78, p<0·0001), and below 2·8 mmol/L by 76% (59–86, p<0·0001), as well as the burden of hypoglycaemia (ie, area under the curve when sensor glucose concentration was less than 3·5 mmol/L) by 73% (59–82, p<0·0001). Closed-loop insulin delivery also reduced the number of nights when glucose concentration was below 3·5 mmol/L for at least 20 min as well as the mean duration of such periods (table 2). Compared with usual pump therapy, closed-loop insulin delivery reduced the proportion of time with glucose concentration above the target range (ie, >10 mmol/L) by 6·9 percentage points (3·5–10·2, p=0·0003), above 13·9 mmol/L by 3·0 percentage points (1·6–4·4, p=0·0002) and above 16·7 mmol/L by 1·2 percentage points (0·6–1·9, p=0·0009; table 2). Moreover, all measures of glycaemic variability were significantly lower in the closed-loop period than in the control period: SD of sensor glucose was 0·5 mmol/L (0·3–0·7) lower (p<0·0001), coefficient of variation of sensor glucose within days was 5·0% (3·0–7·1) lower (p<0·0001), and coefficient of variation of sensor glucose between days was 7·5% (5·3–9·7) lower (p<0·0001; table 2). Total daily insulin was similar between study periods (table 3). Weekly trends in glucose control and insulin delivery are shown in the appendix (p 3).

Outcomes at night time (0000 h to 0600 h) and daytime (0601 h to 2359 h) were in concordance with outcomes from the combined day-and-night period (table 4). Night-time use of closed-loop insulin delivery significantly increased the proportion of time with glucose concentration in target range by 17·2 percentage points (95% CI 12·0–22·4, p<0·0001), reduced mean glucose concentration by 0·4 mmol/L (0·1–0·8, p=0·0211), and decreased the burden of hypoglycaemia by 89% (80–94, p<0·0001) compared with the control period. SD and between-night coefficient of variation of sensor glucose were significantly reduced by closed-loop insulin delivery (table 4). Daytime use of closed-loop insulin delivery increased time spent with glucose concentration in the target range by 8·1 percentage points (95% CI 5·3–11·0, p<0·0001) and reduced the burden of hypoglycaemia by 61% (14–75, p=0·0001). Closed-loop insulin delivery significantly reduced the SD and between-day coefficient of variation of sensor glucose (table 4), in line with measured outcomes of night-time glycaemic variability.

Overall mean absolute relative deviation of sensor glucose, using capillary glucose as the reference, was 15·3% (SD 18·2) and median absolute relative deviation was 10·1% (IQR 4·7–19·3), on the basis of 8447 paired capillary-CGM values. Sensor alarm settings were not altered by participants during closed-loop intervention.

No significant difference was seen in sensor glucose availability between study periods (97% [IQR 95–99] in closed-loop period *vs* 96% [91–97] in control period; p=0·10). Day-and-night closed-loop delivery was used for 90% (95% CI 78–89) of the closed-loop period. The user feedback questionnaire was fully completed by 26 participants, and four of the six questions were answered by all participants (appendix). 27 (93%) of 29 participants were happy to have their glucose levels automatically controlled by the closed-loop system. 20 (69%) participants stated that they spent less time managing their diabetes while using the closed-loop system, but seven (24%) disagreed with this statement. 18 (62%) expressed fewer concerns about their glycaemic control while using the closed-loop system. 14 (48%) participants reported improved sleep during the closed-loop period. 23 (88%) of 26 participants reported feeling safe while using the closed-loop system, and 26 (96%) of 27 would recommend it to others.

No serious adverse events, episodes of severe hypoglycaemia, or episodes of hyperglycaemia with ketosis were reported. Skin irritations related to sensor use occurred in four participants. Two participants had mild respiratory tract infections (one during the run-in period and one during the control period). One participant had cystitis during the closed-loop period and one reported allergic rhinoconjunctivitis during the control period. All reported adverse events were resolved without sequelae.

## Discussion

In this two-centre, open-label, randomised, crossover trial, we showed that, in adults with type 1 diabetes and HbA_1c_ below 7·5%, day-and-night hybrid closed-loop insulin delivery significantly improved overall glucose control while reducing hypoglycaemia progressively by 50–75% at lower glucose thresholds compared with usual insulin pump therapy. Beneficial effects on glycaemic outcomes included increased time spent with glucose concentration in target range (3·9–10·0 mmol/L), reduced time with glucose concentration above and below the target range, and decreased mean glucose concentration and glycaemic variability. The findings of increased time spent in the glucose concentration target range, reduced hypoglycaemia, and decreased glycaemic variability were similarly observed during night-time and daytime periods. These outcomes were achieved without change in total insulin delivery.

Hypoglycaemia is associated with increased morbidity and mortality in patients with type 1 diabetes.^25^ A reduction of at least 30% in risk of hypoglycaemia, as observed in our study, is considered clinically relevant.^26^ Threshold and predictive low-glucose suspend insulin delivery systems^8^, ^9^, ^10^ cannot step-up insulin delivery and thus do not address the issue of hyperglycaemia. The advantage of a closed-loop system such as ours is the responsive, graduated modulation of insulin delivery, both below and above the pre-set pump regimen. This notion is supported by findings from our study, which showed that reduction in mean glucose concentration was accompanied by significant reduction in all the measured hypoglycaemia parameters. The multiplicity of beneficial outcomes—including increased time with optimum glucose control (in target range) and reduced time below and above target range, which were consistently observed during both night-time and daytime periods—suggests that the benefits of the closed-loop system can be accrued irrespective of the time of day in adults with HbA_1c_ below 7·5%. The control algorithm used in our study had enhanced adaptive features and coped safely with variations in insulin requirements, trading variability in insulin delivery for consistency in glucose concentrations. Several studies have shown that increased glycaemic variability is associated with the burden of hypoglycaemia.^27^ Glycaemic variability^28^ and hypoglycaemia^25^ have both been associated with adverse clinical outcomes. We hypothesise that the significant reductions in glucose variability and hypoglycaemia by closed-loop insulin delivery in our study might have implications for clinical outcomes, although this hypothesis will need confirmation by longer and larger studies.

Compared with previous unsupervised home-based studies,^13^, ^16^ our study revealed new findings, specifically in relation to the improvements of CGM-derived hypoglycaemia parameters. Compared with Thabit and colleagues' study of closed-loop insulin delivery versus sensor-augmented pump therapy in patients with type 1 diabetes and HbA_1c_ between 7·5% and 10·0% (58–86 mmol/mol),^13^ our study had more pronounced relative reductions in the proportion of time with glucose concentration below 3·9 mmol/L (19% *vs* 50%) and hypoglycaemia burden (39% *vs* 73%). These differences might have been attributable to the use of an enhanced adaptive control algorithm and tight glycaemic control at baseline in the present study (mean screening HbA_1c_ 6·9% *vs* 8·5% in Thabit and colleagues' study^13^), as well as differences in the control therapies between the two studies. The proportion of time spent with glucose concentration below the target range (ie, 3·9 mmol/L) during usual pump therapy (5·3%, IQR 3·5–10·0) was notably higher in our study than that in Thabit and colleagues' study (3·0%, 1·8–6·1)^13^ but still lower than that in a study in adults with a mean baseline HbA_1c_ of 6·7% (mean proportion of time spent with sensor glucose below target range 14% [SD 11]).^19^ Another study^15^ assessed the use of closed-loop insulin delivery during night time in pregnant women with type 1 diabetes, using a control algorithm with a lower glucose setpoint to accommodate lower glucose targets (3·5–7·8 mmol/L) for this group of patients. In this study,^15^ use of the closed-loop system increased the primary endpoint (time in target range overnight) by 15 percentage points compared with sensor-augmented pump therapy. The difference between this study^15^ and ours is the overnight-only use of closed-loop in the former, and the difference in the comparator between the two studies (sensor-augmented pump therapy *vs* usual pump therapy). Comparability between both studies is also challenging because this specific group of women with type 1 diabetes is highly motivated, albeit over a short time period (ie, during pregnancy), and received intensive dietetic, education, and clinical support.

The additional benefit of closed-loop insulin delivery in individuals with well controlled type 1 diabetes (ie, HbA_1c_ <7·5%) is the reduction of the residual risk of complications, hypoglycaemia, and glycaemic variability, as well as the burden of self-management. Reduction of hypoglycaemic burden has benefits such as improved quality of life and reduced societal cost.^5^, ^29^ Existing reimbursement criteria in several countries for CGM are still predominantly focused on HbA_1c_ and might exclude patients with HbA_1c_ below 7·5% because of the dearth of evidence showing efficacy. However, results from our study show efficacy in terms of both improved glycaemic control and reduced risk of hypoglycaemia. Thus, reimbursement of closed-loop technology in patients with HbA_1c_ below 7·5% could be considered justifiable.

The reduction in hypoglycaemic burden in our study was almost identical to that in Russell and colleagues' study,^30^ which compared 5 day use of a bihormonal (insulin and glucagon) closed-loop system with usual pump therapy in adults with type 1 diabetes. Participants in Russell and colleagues' study had a baseline HbA_1c_ of 7·1% (SD 0·8), and lower mean glucose concentration and risk of hypoglycaemia during the closed-loop period than during usual pump therapy. The study was done under direct supervision during the closed-loop period but not during the control period. The incremental reduction in hypoglycaemia achieved by bihormonal versus single-hormone closed-loop systems needs to be further assessed in longer, unsupervised, head-to-head, randomised studies to justify the increased complexity and cost. A single-hormone hybrid closed-loop system was approved in September, 2016, by the US Food and Drug Administration on the basis of results from a 3 month study^31^ in adolescents and adults with mean HbA_1c_ of 7·4% (SD 0·9) at screening. However, this study was non-randomised and did not have a control group, and the length of study periods was not matched (2 week run-in phase, which was used as baseline comparator for a 3 month intervention).

Results from participants' feedback indicate a high level of trust in the closed-loop system autonomously modulating their glucose concentrations, with the majority agreeing that the time spent on management of diabetes was reduced during the closed-loop period. However, seven (24%) of 29 participants disagreed with this statement, reflecting that user input is still needed for a hybrid closed-loop system. CGM alarms and connectivity issues, especially at night, might have negatively affected participants' experience of the closed-loop system. One participant withdrew from the study because of recurrent technical issues related to the closed-loop system. However, an overall positive endorsement of the closed-loop system was observed, since most participants were willing to recommend the closed-loop system to others.

The strengths of this study include the randomised, two-centre, two-country, crossover design. So far, none of the home-based studies of closed-loop insulin delivery have focused specifically on patients with HbA_1c_ below 7·5% who might be early adopters of closed-loop technologies striving to further improve control of their diabetes. We did not use remote monitoring or direct supervision, so as to adhere to real-world conditions. Additionally, we did not restrict the participants' dietary habits, activity level, and geographical movements. However, we acknowledge that our study has several limitations. The relatively short study duration might have been insufficient to assess long-term compliance. We excluded participants with hypoglycaemia unawareness, therefore restricting assessment of the closed-loop system in those who might benefit greatly. The prototype nature of the closed-loop system and the number of devices might have increased the participants' device burden and negatively affected some aspects of user feedback. The heterogeneity of sensor use in the control period might have confounded the reported glycaemic outcomes. However, the individualised therapy approaches used in the control period reflect present clinical strategies adopted by this population to achieve their baseline HbA_1c_, and do not diminish the incremental effects of closed-loop use.^20^

To conclude, day-and-night closed-loop insulin delivery in adults with type 1 diabetes and HbA_1c_ below 7·5% significantly improved glycaemic control while reducing the risk of hypoglycaemia. Thus, in adults who are actively engaged with self-management, closed-loop insulin delivery might provide additional benefits, justifying its use in this particular population. The overall positive feedback from participants reflected the acceptance of closed-loop technology during daily diabetes management, albeit with some limitations to its use, which might affect user adherence and experience. Larger and longer studies are needed to validate our findings.

## Figures and Tables

**Table 1:** Baseline characteristics

		**Data (n=29)**
Sex		
	Female	15 (52%)
	Male	14 (48%)
Age (years)		41 (13)
Bodyweight (kg)		72·9 (13·0)
BMI (kg/m^2^)		25·1 (3·0)
HbA_1c_ (%)		6·9 (0·5)
HbA_1c_ (mmol/mol)		51·7 (4·8)
Duration of diabetes (years)		24 (12)
Duration using pump (years)		6 (4)
Total daily insulin (U/kg/day)		0·5 (0·1)
Glucose sensor use		
	No previous glucose sensor use	18 (62%)
	Real-time continuous glucose monitoring	5 (17%)
	Flash glucose monitoring	6 (21%)

Data are mean (SD) or n (%).

**Table 2:** Overall day-and-night glucose control during closed-loop and control periods based on sensor glucose measurements

		**Closed-loop period (n=29)**	**Control period (n=28)**	Paired difference*or paired ratio†**(95% CI)**	**p value**
Proportion of time with glucose concentration in range (%)					
	3·9–10·0 mmol/L‡	76·2% (6·4)	65·6% (8·1)	10·5 (7·6 to 13·4)	<0·0001
	>10·0 mmol/L	20·4% (6·3)	27·4% (9·6)	−6·9 (−10·2 to −3·5)	0·0003
	>13·9 mmol/L	3·8% (2·6)	6·9% (3·9)	−3·0 (−4·4 to −1·6)	0·0002
	>16·7 mmol/L	0·9% (0·8)	2·1% (1·8)	−1·2 (−1·9 to −0·6)	0·0009
	<3·9 mmol/L	2·9% (2·3 to 4·0)	5·3% (3·5 to 10·0)	0·50 (0·41 to 0·63)†	<0·0001
	<3·5 mmol/L	1·3% (0·8 to 2·3)	3·4% (1·9 to 7·2)	0·35 (0·26 to 0·47)†	<0·0001
	<3·3 mmol/L	0·9% (0·5 to 1·7)	2·6% (1·3 to 5·5)	0·30 (0·22 to 0·43)†	<0·0001
	<2·8 mmol/L	0·3% (0·1 to 0·5)	1·0% (0·5 to 2·6)	0·24 (0·14 to 0·41)†	<0·0001
AUC_day_ <3·5 mmol/L (min × mmol/L)		9·1 (3·7 to 18·2)	26·7 (13·1 to 65·5)	0·27 (0·18 to 0·41)†	<0·0001
Mean glucose concentration (mmol/L)		7·9 (0·5)	8·3 (0·9)	−0·4 (−0·7 to −0·1)	0·0226
SD of glucose concentration (mmol/L)		2·8 (0·4)	3·3 (0·5)	−0·5 (−0·7 to −0·3)	<0·0001
Coefficient of variation of glucose concentration					
	Within days (%)	35·3 (3·0)	40·3 (5·1)	−5·0 (−7·1 to −3·0)	<0·0001
	Between days (%)	12·8 (3·3)	20·2 (4·6)	−7·5 (−9·7 to −5·3)	<0·0001
Sensor glucose concentration <3·5 mmol/L for at least 20 min					
	Number of nights	2·1 (1·0)	5·6 (3·5)	−3·6 (−4·9 to −2·2)	<0·0001
	Mean duration of each episode (min)	45 (13)	75 (25)	−29 (−38 to −20)	<0·0001

Data are mean (SD) or median (IQR), unless otherwise stated. No significant period effect was observed. AUC_day_=sensor glucose area under the curve per day.

* Unless specified otherwise, data are normally distributed and presented as mean difference of closed-loop period minus control period, with 95% CI for mean; a positive value indicates that the measurement was higher during the closed-loop period than in the control period.

† Non-normally distributed data are presented as ratio of closed-loop data over control data, with 95% CI for ratio; a value greater than unity indicates that the measurement was higher in the closed-loop period than in the control period.

‡ Primary outcome.

**Table 3:** Insulin delivery over 24 h period

	**Closed-loop period (n=29)**	**Control period (n=28)**	Paired difference*(95% CI)	**p value**
Total daily insulin (U/day)	37·5 (13·8)	37·4 (12·6)	0·8 (−1·0 to 2·6)	0·36
Total bolus insulin (U/day)	18·6 (7·9)	20·2 (8·4)	−1·1 (−2·4 to 0·2)	0·11
Total basal insulin (U/day)	18·9 (7·8)	17·2 (5·7)	1·9 (0·7 to 3·1)	0·0038

Data are mean (SD), unless otherwise stated.

* Normally distributed data are presented as mean difference of closed-loop period minus control period, with 95% CI for mean; a positive value indicates that the measurement was higher in the closed-loop period than in the control period.

**Table 4:** Night-time and daytime glucose control during closed-loop and control periods based on sensor glucose measurements

		**Closed-loop period (n=29)**	**Control period (n=28)**	Paired difference*or paired ratio†**(95% CI)**	**p value**
**Night time (0000 h to 0600 h)**					
Proportion of time with glucose concentration in range (%)					
	3·9–10·0 mmol/L	82·0% (9·7)	64·5% (11·5)	17·2 (12·0 to 22·4)	<0·0001
	>10·0 mmol/L	14·9% (8·5)	25·4% (11·8)	−10·2 (−15·4 to −5·1)	0·0004
	<3·9 mmol/L	3·2% (1·6 to 4·0)	9·0% (4·6 to 15·7)	0·33 (0·24 to 0·45)†	<0·0001
	<3·5 mmol/L	1·1% (0·4 to 2·2)	5·4% (2·9 to 11·4)	0·19 (0·12 to 0·28)†	<0·0001
	<2·8 mmol/L	0·1% (0·0 to 0·6)	1·7% (0·9 to 5·7)	0·14 (0·08 to 0·26)†	<0·0001
AUC_day_ <3·5 mmol/L (mmol/L × min)		5·1 (1·4 to 15·5)	41·5 (21·5 to 122·8)	0·11 (0·06 to 0·20)†	<0·0001
Mean glucose concentration (mmol/L)		7·5 (0·6)	8·0 (1·0)	−0·4 (−0·8 to −0·1)	0·0211
SD of glucose concentration (mmol/L)		2·5 (0·6)	3·2 (0·6)	−0·7 (−1·0 to −0·4)	<0·0001
Coefficient of variation of glucose concentration between nights (%)		24·9 (7·1)	34·4 (6·4)	−9·6 (−13·2 to −5·9)	<0·0001
**Daytime (0601 h to 2359 h)**					
Proportion of time with glucose concentration in range (%)					
	3·9–10·0 mmol/L	74·3% (6·9)	66·1% (8·8)	8·1 (5·3 to 11·0)	<0·0001
	>10·0 mmol/L	22·2% (7·2)	27·9% (10·4)	−5·6 (−8·9 to −2·3)	0·0023
	<3·9 mmol/L	2·7% (1·9 to 4·5)	4·4% (2·8 to 8·6)	0·61 (0·49 to 0·76)†	0·0001
	<3·5 mmol/L	1·2% (0·7 to 2·2)	2·3% (1·4 to 6·0)	0·47 (0·35 to 0·64)†	<0·0001
	<2·8 mmol/L	0·2% (0·1 to 0·5)	0·5% (0·2 to 1·3)	0·48 (0·29 to 0·80)†	0·0076
AUC_day_ <3·5 mmol/L (mmol/L × min)		8·3 (3·4 to 14·3)	15·9 (7·5 to 45·7)	0·39 (0·25 to 0·86)†	0·0001
Mean glucose concentration (mmol/L)		8·0 (0·6)	8·4 (1·0)	−0·3 (−0·6 to 0·0)	0·0498
SD of glucose concentration (mmol/L)		2·9 (0·4)	3·3 (0·6)	−0·5 (−0·7 to −0·2)	0·0002
Coefficient of variation of glucose concentration between days (%)		13·8 (3·0)	20·6 (5·1)	−6·8 (−9·1 to −4·6)	<0·0001

Data are presented as mean (SD) or median (IQR), unless otherwise stated. No significant period effect was observed. AUC_day_=sensor glucose area under the curve per day.

* Unless specified otherwise, data are normally distributed and presented as mean difference of closed-loop period minus control period, with 95% CI for mean; a positive value indicates that the measurement was higher in the closed-loop period than in the control period.

† Non-normally distributed data are presented as ratio of closed-loop data over control data, with 95% CI for ratio; a value greater than unity indicates that the measurement was higher in the closed-loop period than in the control period.
