# Supplementary material for: Day-and-night glycaemic control with closed-loop insulin delivery versus conventional insulin pump therapy in free-living adults with well controlled type 1 diabetes: an open-label, randomised, crossover study
Source: Lancet Diabetes Endocrinol. 2017 Apr;5(4):261–70. doi: 10.1016/S2213-8587(17)30001-3 (PMC5379244; doi:10.1016/S2213-8587(17)30001-3)
Supplement: Supplementary appendix [file mmc1.pdf]

THE LANCET
Diabetes & Endocrinology

Supplementary appendix

This appendix formed part of the original submission and has been peer reviewed. We post it as supplied by the authors.

Supplement to: Bally L, Thabit H, Kojzar H, et al. Day-and-night glycaemic control with closed-loop insulin delivery versus conventional insulin pump therapy in free-living adults with well controlled type 1 diabetes: an open-label, randomised, crossover study. *Lancet Diabetes Endocrinol* 2017; published online Jan 13. http://dx.doi.org/10.1016/S2213-8587(17)30001-3.

Figure S1. Study design comparing 24/7 closed-loop insulin delivery with control.

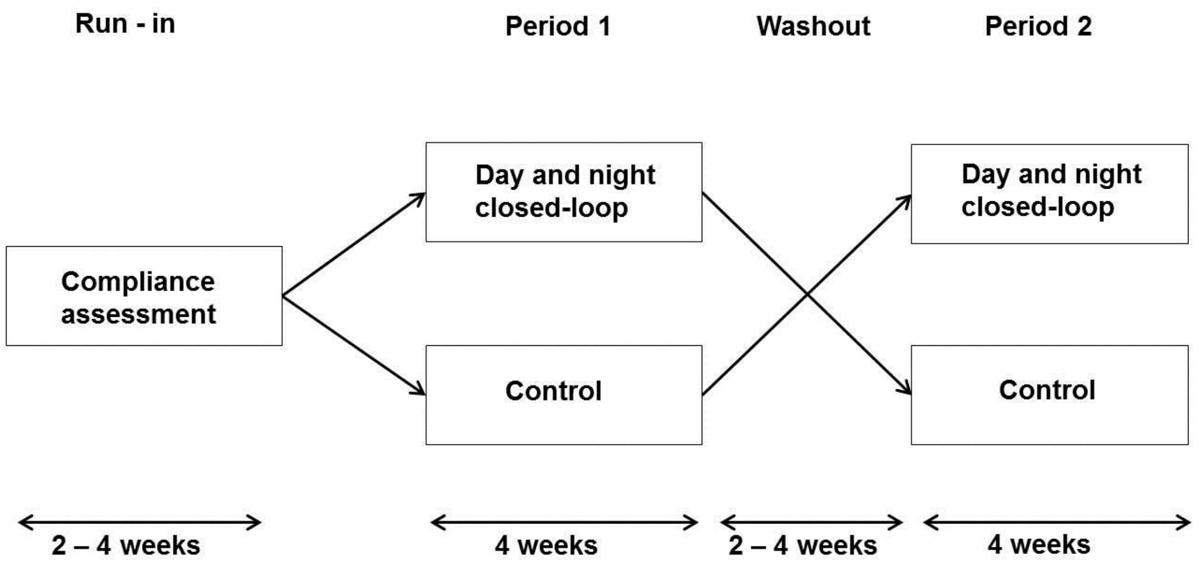

Figure S2. FlorenceD2A closed-loop system used in the study.

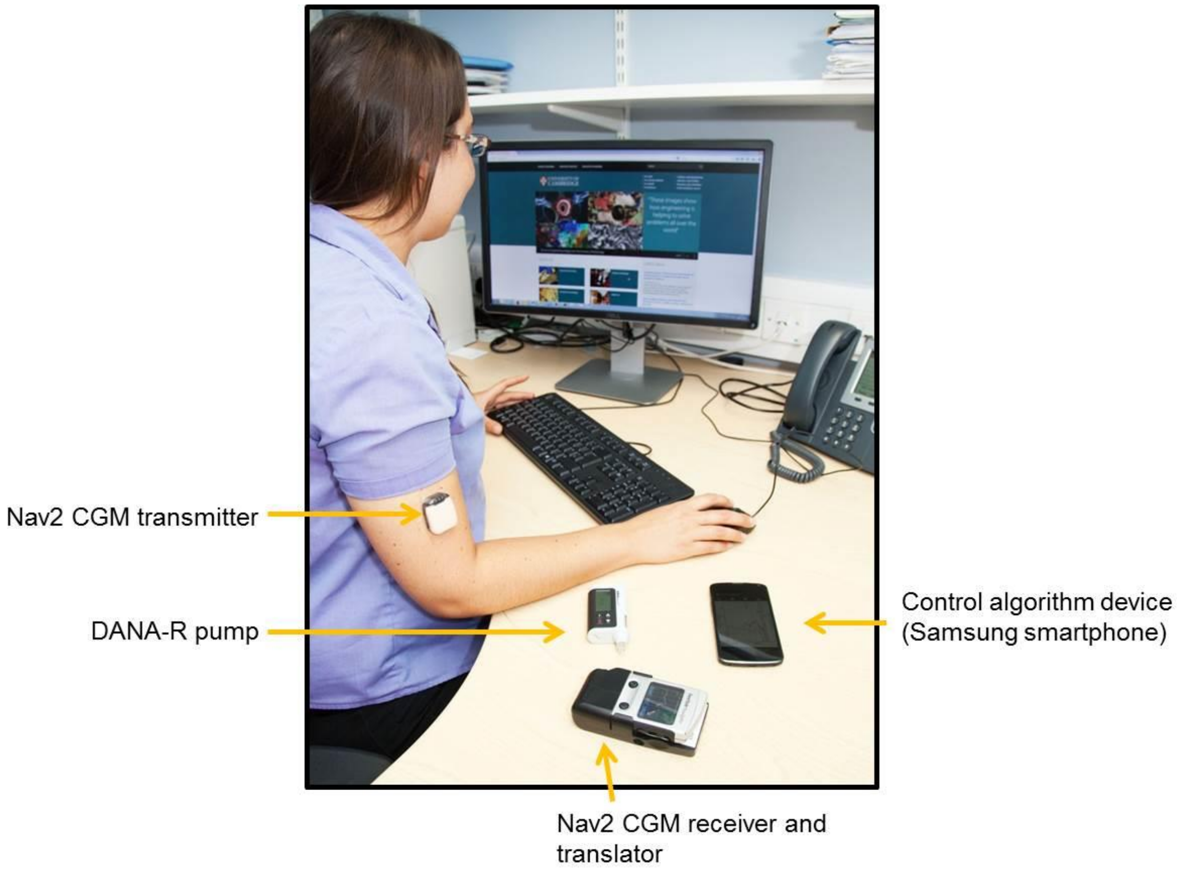

Figure S3. Weekly trend of the proportion of time when sensor glucose was in target range between 3.9 and 10mmol/l (top) and total daily insulin delivered (bottom) during closed-loop (red shaded boxes) and control (grey shaded boxes). Mean (SD) is shown.

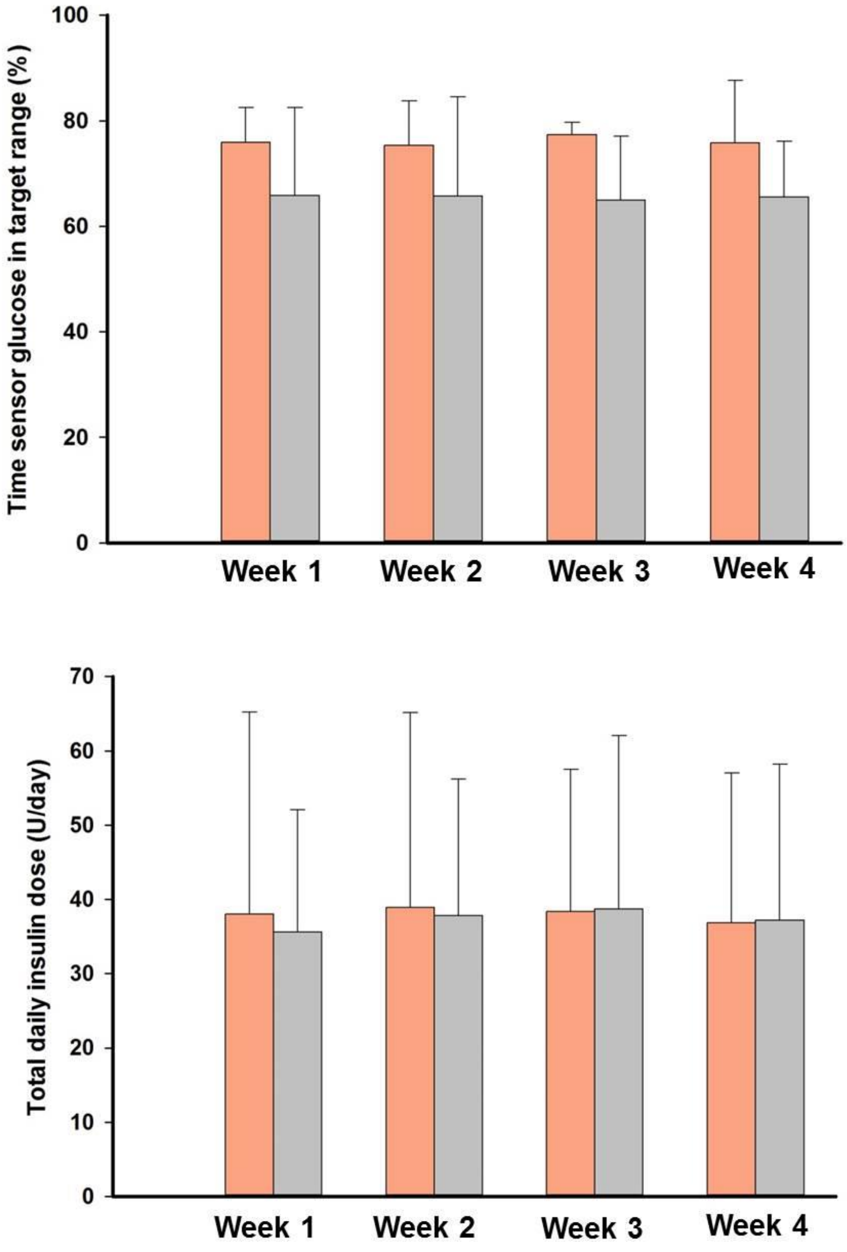

Figure S4. Participants' responses to feedback questionnaire.

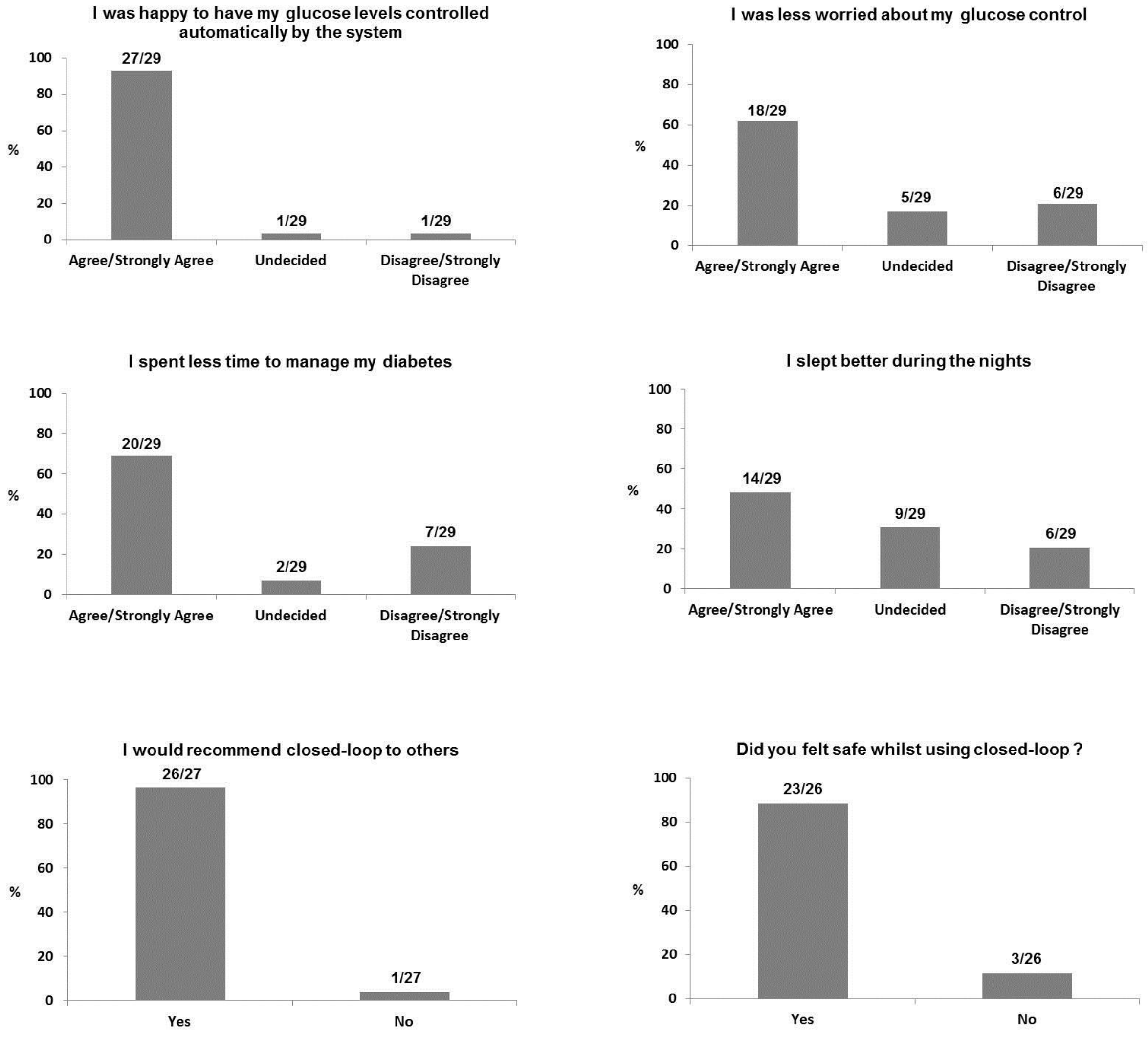

Table S1. Glycaemic endpoints based on pre-study sensor usage.

	Sensor-naïve		Real time continuous glucose monitoring		Flash glucose monitoring	
	**Closed-loop (n=18)**	**Control (n=17)**	**Closed-loop (n=5)**	**Control (n=5)**	**Closed-loop (n=6)**	**Control (n=6)**
**Time spent at glucose level 3.9 to 10 mmol/l (%)**	76.0 (6.4)	64.2 (6.5)	73.2 (8.2)	67.3 (12.5)	79.4 (4.3)	68.4 (8.8)
**Mean glucose (mmol/l)**	7.9 (0.6)	8.3 (0.9)	8.2 (0.5)	8.4 (0.8)	7.7 (0.4)	8.0 (1.2)
**Time spent at glucose level<3.9 mmol/l (%)**	3.5 (2.3 to 5.0)	5.9 (4.0 to 9.6)	2.3 (1.9 to 2.5)	3.9 (3.1 to 5.8)	2.7 (2.5 to 3.2)	5.1 (3.1 to 13.0)
**AUC_{day} < 3.5 mmol/l (mmol/l x min)***	9.2 (3.7 to 20.1)	27.5 (17.0 to 76.9)	7.6 (3.6 to 19.5)	17.6 (13.3 to 32.7)	9.3 (6.5 to 10.0)	32.4 (9.9 to 62.7)
**SD of glucose (mmol/l)**	2.8 (0.4)	3.4 (0.4)	2.9 (0.4)	3.2 (0.7)	2.7 (0.2)	3.2 (0.6)
**CV of glucose (%)**	35.3 (3.1)	41.4 (4.0)	35.8 (4.7)	37.6 (6.1)	34.9 (1.8)	39.8 (6.7)

Data are presented as mean (SD), or median (interquartile range)
*AUC_{day}, Glucose area under curve below 3.5mmol/l per day

Metabolic Research Laboratories

Clinical Study Protocol

Study Title: **An open-label, multi-centre, randomised, two-period, crossover study to assess the efficacy, safety and utility of day and night automated closed-loop glucose control under free living conditions compared to conventional insulin pump therapy combined with continuous glucose monitoring in adults with type 1 diabetes and HbA1c between 7.5% and 10% (phase 1) and HbA1c below 7.5% (phase 2)**

Short Title: **Closing the loop in adults with type 1 diabetes under free living conditions**

Protocol Version: **3.0 12 November 2015**
Protocol ID: **AP@home04**

Chief Investigator	Dr Roman Hovorka<br>Wellcome Trust-MRC Institute of Metabolic Science<br>University of Cambridge<br>Box 289, Level 4<br>Addenbrooke's Hospital<br>Hills Road, Cambridge<br>CB2 0QQ<br>UK<br>Phone: +44 1223 762 862<br>Fax: +44 1223 336 996<br>rh347@cam.ac.uk

This protocol has been written in accordance with current ISO 14155:2011 standard

Principal Clinical Investigators	Dr Mark Evans<br>Wellcome Trust-MRC Institute of Metabolic Science<br>Box 289, Level 4<br>Addenbrooke's Hospital<br>Hills Road<br>Cambridge CB2 0QQ<br>UK<br>Phone: +44 1223 336 994<br>Fax: +44 1223 336 996<br>mle24@cam.ac.uk<br><br>Dr Sabine Arnolds<br>Profil Institut für Stoffwechselforschung GmbH<br>Hellersbergstr. 9<br>D-41460 Neuss<br>Germany<br>Phone: + 49 2131 4018 401<br>Fax: +49 2131 4018 501<br>sabine.arnolds@profil.com<br><br>Dr Thomas Pieber<br>Medical University of Graz,<br>Dept. of Internal Medicine<br>Division of Endocrinology and Metabolism<br>Auenbruggerplatz 15<br>A- 8036 Graz<br>Austria<br>Phone: +43 3163 8512 383<br>Fax: +43 3163 8513 428<br>thomas.pieber@medunigraz.at
Clinical Investigators	Dr Hood Thabit<br>Wellcome Trust-MRC Institute of Metabolic Science<br>Box 289, Level 4<br>Addenbrooke's Hospital<br>Hills Road, Cambridge<br>CB2 0QQ<br>UK<br>Tel: +44 (0)1223 769 074<br>Fax: +44 (0)1223 336 996<br>ht312@medschl.cam.ac.uk

	Dr Lalantha Leelarathna<br>Wellcome Trust-MRC Institute of Metabolic Science<br>Box 289, Level 4<br>Addenbrooke's Hospital<br>Hills Road, Cambridge<br>CB2 0QQ<br>UK<br>Tel: +44 (0)1223 769 077<br>Fax: +44 (0)1223 336 996<br>lhl24@medschl.cam.ac.uk<br><br>Dr Julia Mader<br>Medical University of Graz,<br>Dept. of Internal Medicine<br>Division of Endocrinology and Metabolism<br>Auenbruggerplatz 15<br>A- 8036 Graz<br>Austria<br>Phone: +43 316 385 80254Fax: +43 316 385 13428<br>julia.mader@medunigraz.at<br><br>Dr Sibylle Dellweg<br>Profil Institut für Stoffwechselforschung GmbH<br>Hellersbergstr. 9<br>D-41460 Neuss<br>Germany<br>Phone: + 49 (0) 2131 4018 428<br>Fax: +49 (0) 2131 4018 545<br>sibylle.dellweg@profil.com
Other Investigators	Dr Malgorzata E Wilinska<br>Wellcome Trust-MRC Institute of Metabolic Science<br>University of Cambridge<br>Box 289, Level 4<br>Addenbrooke's Hospital<br>Hills Road, Cambridge<br>CB2 0QQ<br>UK<br>Phone: +44 1223 769 065<br>Fax: +44 1223 336 996<br>mew37@cam.ac.uk

Study Psychologists	Dr Katharine Barnard<br>Health Psychologist and Senior Research Fellow,<br>Human Development and Health<br>Faculty of Medicine, University of Southampton<br>IDS Building, Southampton General Hospital<br>Tremona Road, Southampton, SO16 6YD<br>UK<br>Tel: +44 (0)2380 595 000 / +44 (0)7590532866<br>E-mail: k.barnard@soton.ac.uk<br><br>Dr Tim Wysocki<br>Centre for Pediatric Psychology Research<br>Nemours Children's Clinic<br>807 Children's Way<br>Jacksonville, FL 32207-8426<br>USA<br>Tel: +1 (904) 697-4034 (Research); +1 (904) 390-3415 (IRB)<br>Fax: +1 (904) 390-3425<br>E-mail: twysocki@nemours.org	
Clinical Laboratories	Ms Karen Whitehead<br>Chief Research Technician<br>University of Cambridge<br>Department of Paediatrics<br>Level 8 Box 116<br>Addenbrooke's Hospital<br>Cambridge CB2 0QQ<br>Tel: + 44 (0) 1223 762945<br>Fax:+ 44 (0) 1223 336996<br>Email kw240@cam.ac.uk	
Study Sponsor	Cambridge University Hospitals NHS Foundation Trust, jointly with University of Cambridge	
	Jo Martindale<br>Research Operations Office<br>University of Cambridge<br>16 Mill Lane,<br>Cambridge, CB2 1SB,<br>UK<br>Phone: +44 (0)1223333358<br>Fax: +44 (0)1223 332988<br>jo.martindale@admin.cam.ac.uk	Stephen Kelleher<br>Cambridge University Hospitals NHS Foundation Trust<br>Box 277, Addenbrooke's Hospital<br>Hills Road, Cambridge, CB2 0QQ,<br>UK<br>Phone: +44 (0) 1223 217418<br>Fax: +44 (0) 1223 348494<br>r&denquiries@addenbrookes.nhs.uk

Funder	European Commission within the 7th Framework Programme

Data Monitoring and Ethics Committee

**<u>Chair</u>**<br><br>**Prof John Pickup**	Metabolic Unit<br>5th Floor, Southwark Wing<br>Guy's Hospital<br>London, SE1 9RT<br>UK<br>Tel: +44 (0)20 7188 3859<br>Fax: +44 (0)20 7188 0146<br>E-mail: john.pickup@kcl.ac.uk
**<u>Medical Expert</u>**<br><br>**Prof Irl Hirsch**	Division of Metabolism, Endocrinology and Nutrition<br>University of Washington School of Medicine<br>1959 NE Pacific<br>UW Mailbox 354691<br>Seattle, WA 98195-4691<br>USA<br>Tel: +1 (206) 598-4882<br>Fax: +1 (206) 598-4976<br>E-mail: ihirsch@u.washington.edu
**<u>Medical Expert</u>**<br><br>**Dr Howard Wolpert**	Joslin Diabetes Center<br>One Joslin Place,<br>Boston, MA 02215<br>USA<br>Tel: +1 (617) 732-2400<br>E-mail: howard.wolpert@joslin.harvard.edu

1 PROTOCOL SIGNATURE PAGE

The signature below documents the approval of protocol Version …. Dated …………. and provides the necessary assurances that this study will be conducted according to all stipulations of the protocol, the principles of GCP and the appropriate reporting requirements.

Signature ……………………………………. Date……………………….

Dr Roman Hovorka

Chief Investigator /Sponsor representative

1.1 Site signature (Cambridge, UK)

I have read the attached protocol entitled "**An open-label, multi-centre, randomised, two-period, crossover study to assess the efficacy, safety and utility of day and night automated closed-loop glucose control under free living conditions compared to conventional insulin pump therapy combined with continuous glucose monitoring in adults with type 1 diabetes and HbAlc between 7.5% and 10% (phase 1) and HbA1c below 7.5% (phase 2)"** Version ..... dated ……….., and agree to abide by all provisions set forth therein.

I agree to comply with the International Conference on Harmonisation Tripartite Guideline on Good Clinical Practice: The European Clinical Trials Directives 2001/20/EC and 2005/28/EC.

I agree to ensure that the confidential information contained in this document will not be used for any other purpose other than the evaluation or conduct of the clinical investigation without the prior written consent of the Sponsor.

Signature ……………………………………. Date……………………….

Dr Mark Evans

Principal Clinical Investigator

1.2 Site signature (Neuss, Germany)

I have read the attached protocol entitled "**An open-label, multi-centre, randomised, two-period, crossover study to assess the efficacy, safety and utility of day and night automated closed-loop glucose control under free living conditions compared to conventional insulin pump therapy combined with continuous glucose monitoring in adults with type 1 diabetes and HbAlc between 7.5% and 10% (phase 1) and HbA1c below 7.5% (phase 2)"** Version ..... dated ……….., and agree to abide by all provisions set forth therein.

I agree to comply with the International Conference on Harmonisation Tripartite Guideline on Good Clinical Practice: The applicable German (e.g. MPG, MPKPV, DIN EN ISO 14155) and European legislation (e.g. 93/42/EC).

I agree to ensure that the confidential information contained in this document will not be used for any other purpose other than the evaluation or conduct of the clinical investigation without the prior written consent of the Sponsor.

Signature ……………………………………. Date……………………….

Dr Sabine Arnolds
Principal Clinical Investigator

1.3 Site signatures (Graz, Austria)

I have read the attached protocol entitled "**An open-label, multi-centre, randomised, two-period, crossover study to assess the efficacy, safety and utility of day and night automated closed-loop glucose control under free living conditions compared to conventional insulin pump therapy combined with continuous glucose monitoring in adults with type 1 diabetes and HbAlc between 7.5% and 10% (phase 1) and HbA1c below 7.5% (phase 2)"** Version ..... dated ……….., and agree to abide by all provisions set forth therein.

I agree to comply with the International Conference on Harmonisation Tripartite Guideline on Good Clinical Practice: The European Clinical Trials Directives 2001/20/EC and 2005/28/EC.

I agree to ensure that the confidential information contained in this document will not be used for any other purpose other than the evaluation or conduct of the clinical investigation without the prior written consent of the Sponsor.

Signature ……………………………………. Date……………………….

Dr Thomas Pieber
Principal Clinical Investigator

2 List of Abbreviations and Relevant Definitions

**ADE**	**Adverse device / method effect**
**AE**	**Adverse Event**
**AR**	**Adverse Reaction**
**ASADE**	**Anticipated Serious Adverse Device Effect**
**CE**	**Conformité Européenne (CE-mark)**
**CGM**	**Continuous Glucose Monitoring**
**CL**	**Closed-loop**
**CRC**	**Clinical Research Centre**
**CRF**	**Case Report Form**
**MHRA**	**Medicines & Healthcare products Regulatory Agency (UK)**
**MPC**	**Model predictive control algorithm**
**MPG**	**Medical Devices Act (Medizinproduktegesetz)**
**MPKPV**	**Ordinance on Clinical Investigations of Medical Devices (Verordnung über klinische Prüfungen von Medizinprodukten)**
**REC**	**Research Ethics Committee**
**OL**	**Open-loop (usual treatment)**
**s.c.**	**Subcutaneous**
**SADE**	**Serious adverse device / method effect**
**SAE**	**Serious Adverse Event**
**scCGM**	**Subcutaneous Continuous Glucose Monitoring**
**SMBG**	**Self Monitored Blood Glucose**
**SUSAR**	**Suspected Unexpected Serious Adverse Reaction**
**T1D**	**Type 1 Diabetes Mellitus**
**USADE**	**Unanticipated Serious Adverse Device Effect**

3 Study Synopsis

**Title of clinical trial**	**An open-label, multi-centre, randomised, two-period, crossover study to assess the efficacy, safety and utility of automated closed-loop glucose control under free living conditions compared to conventional insulin pump therapy combined with continuous glucose monitoring in adults with type 1 diabetes and HbAlc between 7.5% and 10% (phase 1) and HbA1c below 7.5% (phase 2)**
**Short Title**	Closing the loop in adults with type 1 diabetes under free living conditions
**Sponsor name**	University of Cambridge and Cambridge University Hospitals NHS Foundation Trust
**Medical condition or disease under investigation**	Type 1 diabetes
**Purpose of clinical trial**	To determine whether day and night closed-loop insulin delivery under free living conditions is superior to addition of real-time continuous glucose monitoring in adults with type 1 diabetes on insulin pump therapy.
**Study objectives**	The study objective is to compare day and night automated closed-loop glucose control with conventional insulin pump therapy combined with continuous glucose monitoring (CGM) under free living conditions.<br><br>**1. EFFICACY:** The objective is to assess the efficacy of day and night automated closed-loop glucose control in maintaining CGM glucose levels within the target range from 3.9 to 10.0 mmol/l, as compared to conventional insulin pump therapy combined with CGM.<br><br>**2. SAFETY:** The objective is to evaluate the safety of day and night automated closed-loop glucose control in terms of episodes of severe hypoglycaemia, hyperglycaemia and other adverse events and adverse device effects.<br><br>**3. UTILITY:** The objective is to determine the percentage of time when closed-loop was operational, and usability and acceptance of the closed-loop system.
**Study Design**	An open-label, multi-centre, randomised, two-period

	crossover study, contrasting day and night automated closed-loop glucose control with conventional insulin pump therapy combined with CGM
**Study Efficacy Endpoints**	The primary outcome is the time spent in the target glucose range from 3.9 to 10.0 mmol/l based on CGM glucose levels during the free living phase.<br><br>Secondary outcomes include time spent above and below the target glucose range, based on CGM levels.
**Safety Evaluation**	Frequency of severe hypoglycaemic episodes as defined by American Diabetes Association, frequency of severe hyperglycaemia (>20 mmol/l) and / or significant ketosis (plasma ketones >3mmol/l) and nature and severity of other adverse events.
**Utility Evaluation**	Percentage of time spent in closed-loop. Usability and acceptance of the closed-loop system will be assessed during the 12 week (phase 1 only) free living phase using Diabetes Technology Questionnaire, quality of life-style change, daily diabetes management and fear of hypoglycaemia. In phase 2, a patient experience questionnaire will be given at the end of the closed-loop glucose control intervention period.
**Sample Size**	30 adults completing phase 1 and 24 adults completing phase 2 of the study. Up to 42 (phase 1) and 34 (phase 2) subjects will be recruited to allow for dropouts.
**Summary of eligibility criteria**	Key inclusion criteria:<br>1. Type 1 diabetes diagnosed for at least 1 year<br>2. Aged 18 years and older<br>3. Insulin pump treatment for at least 6 months<br>4. HbA1c ≥7.5% (58mmol/mol) and ≤ 10% (86 mmol/mol) for phase 1 and <7.5% (58 mmol/mol) for phase 2, based on analysis from central laboratory or equivalent<br><br>Key exclusion criteria:<br>1. Physical or psychological disease likely to interfere with the normal conduct of the study<br>2. Current treatment with drugs known to have significant interference with glucose metabolism<br>3. Total daily insulin dose ≥ 2 IU/kg/day<br>4. Subject is using real-time CGM on regular

	basis (phase 1 only)<br>5. Pregnancy, planned pregnancy, or breast feeding<br>6. Significantly reduced hypoglycaemia awareness (Gold score > 4)<br>7. Severe visual impairment<br>8. Severe hearing impairment<br>9. More than one episode of severe hypoglycaemia as defined by American Diabetes Association in preceding 12 months<br>10. Random C-peptide > 100pmol/l with concomitant plasma glucose >4 mM(72 mg/dl)
**Maximum duration of study for a subject**	40 weeks (10 months) for phase 1 and 16 weeks (4 months) for phase 2
**Recruitment**	The subjects will be recruited through the adult diabetes outpatient clinics or other established methods at participating centres.
**Consent**	Participants will be asked to provide written informed consent.
**Baseline Assessment**	Eligible subjects will undergo a baseline evaluation including a blood sample for the measurement of HbA1c, renal, liver functions, full blood count, thyroid functions and coeliac antibody screen (if not done in the previous 3 months). Urine pregnancy test will be done in females. Additional centre specific assessments will also be undertaken.
**Study Training and Run-in Period**	Training sessions on the use of study CGM, insulin pump and closed-loop system will be provided by the research team. During the 6-8 weeks (phase 1) and 2-4 weeks (phase 2) run-in period, subjects will use study CGM and insulin pump and will have regular contact with the research team. At the end of the run-in period, for compliance and to assess the ability of the subject to use the CGM and study pump safely, before the start of the first home study phase, at least 7 days of CGM data need to be recorded and safe use of study insulin pump demonstrated. CGM and insulin pump data during the run-in period will be used to assess baseline glucose control and optimise treatment before the start of the first home study phase.
**Competency Assessment**	Competency on the use of study insulin pump, study CGM and closed-loop system will be evaluated using a competency assessment tool developed by the research team. Further training may be delivered as required.

**Randomisation**	Eligible subjects will be randomised using randomisation software to the use of automated closed-loop glucose control or to conventional pump therapy with CGM (blinded in phase 2), with a washout period of 4-6 weeks (phase 1) and 2-4 weeks (phase 2) in between the two interventions.
**1. Automated closed-loop**	Subjects will be admitted to the Clinical Research Facility on Day 1. Training on the use of closed-loop will be provided by the research team. Automated closed-loop control will be commenced and during the next 4-6 hours patient will operate the system under the supervision of the clinical team. Competency on the use of closed-loop system will be evaluated. Subjects will be advised to use automated closed-loop system for next 12 weeks(phase 1) or 4 weeks (phase 2).
**Cross-over Assessment**	At the end of the first intervention, a blood sample for the measurement of HbA1c will be taken. In the phase 1 study, usability and acceptance of the closed-loop system will be assessed using Diabetes Technology Questionnaire as well as other questionnaires to assess quality of life change, daily diabetes management and fear of hypoglycaemia. In phase 2, a patient experience questionnaire will be given at the end of the closed-loop glucose control intervention period.
**Wash-out period**	At the end of the first intervention, subjects will undergo a wash-out period of 4 to 6 weeks in phase 1 and 2 to 4 weeks in phase 2, during which they may continue to wear the continuous glucose monitor and the study insulin pump and their standard pump settings will be applied.
**2. Conventional insulin pump therapy with CGM**	In phase 1, a training session on the use of real-time CGM and written guidelines on how to interpret real-time and retrospective stored data will be provided to all subjects/carers. Subjects will use conventional insulin pump therapy and real-time CGM for 12 weeks. In phase 2, subjects will use conventional insulin pump therapy and blinded (masked) CGM for 4 weeks. Subjects will perform CGM calibration according to manufacturer's instructions.
**End of study assessments**	A blood sample will be taken for measurement of HbA1c. In phase 1, usability and acceptance of the conventional insulin pump therapy and real-time CGM will be assessed using Diabetes Technology Questionnaire as well as other questionnaires to assess quality of life change, daily diabetes

	management and fear of hypoglycaemia. In phase 2, a patient experience questionnaire will be given at the end of the closed-loop glucose control intervention period.
**Procedures for safety monitoring during trial**	Standard operating procedures for monitoring and reporting of all adverse events and adverse device events will be in place, including serious adverse events (SAE), serious adverse device effects (SADE) and specific adverse events (AE) such as severe hypoglycaemia.<br><br>Subjects will be asked to test and record blood ketones if their finger-stick glucose is above 14 mmol/l as part of the safety assessment for hyperglycaemia.<br><br>A data monitoring and ethics committee (DMEC) will be informed of all serious adverse events and any unanticipated adverse device/method effects that occur during the study and will review compiled adverse event data at periodic intervals.
**Criteria for withdrawal of patients on safety grounds**	A subject may terminate participation in the study at any time without necessarily giving a reason and without any personal disadvantage. An investigator can stop the participation of a subject after consideration of the benefit/risk ratio. Possible reasons are:<br>• Serious adverse events<br>• Significant protocol violation or non-compliance<br>• Failure to satisfy competency assessment<br>• Decision by the investigator, or the sponsor, that termination is in the subject's best medical interest<br>• Pregnancy, planned pregnancy, or breast feeding<br>• Allergic reaction to insulin<br>• Technical grounds (e.g. subject relocates)

Table of Contents

1 **PROTOCOL SIGNATURE PAGE** 7
1.1 SITE SIGNATURE (CAMBRIDGE, UK) 8
1.2 SITE SIGNATURE (NEUSS, GERMANY) 9
1.3 SITE SIGNATURES (GRAZ, AUSTRIA) 10
2 **LIST OF ABBREVIATIONS AND RELEVANT DEFINITIONS** 11
3 **STUDY SYNOPSIS** 12
4 **SUMMARY** 20
5 **BACKGROUND** 21
5.1 CLOSED-LOOP RESEARCH IN CAMBRIDGE, UK 22
5.2 AP@HOME CONSORTIUM 23
5.3 MANUAL OPERATIONAL MODE 23
5.4 AUTOMATED CLOSED-LOOP MODE 23
5.5 MOVING FROM CLINICAL RESEARCH FACILITY TO HOME ENVIRONMENT: CAMBRIDGE HOME STUDIES 24
5.6 DAY AND NIGHT CLOSED LOOP INSULIN DELIVERY UNDER FREE LIVING CONDITIONS USING FLORENCED SYSTEM 24
5.7 AUTOMATED CLOSED-LOOP SYSTEM TO BE USED IN THE PRESENT STUDY 25
6 **OBJECTIVES** 27
6.1 EFFICACY 27
6.2 SAFETY 27
6.3 UTILITY 27
7 **STUDY DESIGN** 27
8 **STUDY SUBJECTS** 27
8.1 STUDY POPULATION 27
8.2 RECRUITMENT AND INFORMED CONSENT 27
8.2.1 Inclusion criteria 28
8.2.2 Exclusion criteria for all countries 29
8.2.3 Additional exclusion criteria specific for Austria and Germany 29
8.2.4 Additional exclusion criteria specific for Germany only 30
8.3 RANDOMISATION 30
9 **METHODS UNDER INVESTIGATION** 32
9.1 NAME AND DESCRIPTION OF THE METHOD OF INVESTIGATION 32
9.2 INTENDED PURPOSE 32
9.3 METHOD OF ADMINISTRATION 32
9.4 REQUIRED TRAINING 32
9.5 PRECAUTIONS 32
9.6 ACCOUNTABILITY OF THE METHOD UNDER INVESTIGATION 33
10 **STUDY SCHEDULE** 33
10.1 OVERVIEW 33
10.2 RECRUITMENT VISIT (VISIT 1) 38
10.3 VISIT 2 - TRAINING SESSION ON THE USE OF THE STUDY INSULIN PUMP 39
10.4 VISIT 3 - TRAINING SESSION ON THE CONTINUOUS GLUCOSE MONITORING (CGM) AND STUDY PUMP 39
10.5 VISIT 4 – FURTHER DEVICE TRAINING, REVIEW OF DATA AND START OF OPTIMISATION PERIOD 40
10.6 VISITS 5, 6 AND 7 – TREATMENT OPTIMISATION PERIOD 40
10.7 VISIT 8 (CLOSED-LOOP VISIT; WITHIN 1 WEEK OF VISIT 7 AND AT LEAST 4 WEEKS AFTER VISIT 3) 41
10.8 VISIT 9 – 1 WEEK AFTER STARTING CL TREATMENT 42
10.9 VISIT 10 – 2 WEEKS AFTER STARTING CL TREATMENT 42
10.10 VISIT 11 – END OF FIRST MONTH ON CL TREATMENT 43
10.11 VISIT 12 – END OF SECOND MONTH ON CL TREATMENT 43
10.12 VISIT 13 – END OF CL TREATMENT 43
10.13 VISIT 14 (OPTIONAL) 43
10.14 WASHOUT 43
10.15 VISIT 15 (CONTROL VISIT) 44
10.16 VISIT 16 – 1 WEEK AFTER STARTING OL TREATMENT 44
10.17 VISIT 17 – 2 WEEKS AFTER STARTING OL TREATMENT 44
10.18 VISIT 18 – END OF FIRST MONTH ON OL TREATMENT 44
10.19 VISIT 19 – END OF SECOND MONTH ON OL TREATMENT 45
10.20 VISIT 20 – END OF OL TREATMENT 45
10.21 PARTICIPANT WITHDRAWAL CRITERIA 53
10.22 STUDY STOPPING CRITERIA 54
10.23 SUPPORT TELEPHONE LINE 54
10.24 SUBJECT REIMBURSEMENT 54
11 **ENDPOINTS** 54
11.1 EFFICACY ENDPOINTS 54

11.1.1 *Primary efficacy endpoint* ... 54
11.1.2 *Secondary efficacy endpoints* ... 55
11.2 SAFETY EVALUATION ... 55
11.3 UTILITY EVALUATION ... 56
12 ASSESSING AND REPORTING OF ADVERSE EVENTS ... 57
12.1 DEFINITIONS ... 57
12.1.1 *Reportable Adverse Events* ... 57
12.1.2 *Adverse Events* ... 57
12.1.3 *Adverse Device Effect* ... 57
12.1.4 *Serious Adverse Event* ... 57
12.1.5 *Serious Adverse Device Effect* ... 58
12.1.6 *Unanticipated Serious Adverse Device Effect* ... 58
12.1.7 *Device Deficiencies* ... 58
12.1.8 *Adverse Event Intensity* ... 59
12.1.9 *Adverse Event Causality* ... 59
12.2 RECORDING AND REPORTING OF ADVERSE EVENTS, SERIOUS ADVERSE EVENTS AND DEVICE DEFICIENCIES ... 60
12.2.1 *Monitoring Period of Adverse Events* ... 60
12.2.2 *Recording and reporting of Adverse Events* ... 60
12.2.3 *Severe Hypoglycaemia* ... 61
12.2.4 *Reporting of Serious Adverse Events and Serious Adverse Device Effects* ... 61
12.2.5 *Recording and Reporting of Device Deficiencies* ... 63
12.2.6 *Healthcare Arrangements and Compensation for Adverse Events* ... 63
12.2.7 *Country specific requirements:* ... 63
12.3 RISKS AND DISCOMFORTS AND POTENTIAL / ANTICIPATED ADVERSE EVENTS AND ADVERSE DEVICE EVENTS/EFFECTS ... 64
12.3.1 *Hypoglycaemia and hyperglycaemia* ... 64
12.3.2 *Finger-prick blood glucose measurements* ... 65
12.3.3 *Insulin pump therapy* ... 65
12.3.4 *Venepuncture* ... 65
12.3.5 *Continuous glucose monitoring* ... 65
12.3.6 *Questionnaires* ... 66
12.3.7 *Burdens* ... 66
12.3.8 *Risk analysis and residual risk associated with the investigational device* ... 66
12.4 BENEFITS ... 67
12.5 DATA MONITORING AND ETHICS COMMITTEE ... 67
13 METHODS AND ASSESSMENTS ... 68
13.1 PROCEDURES ... 68
13.1.1 *Height and weight* ... 68
13.1.2 *Continuous subcutaneous glucose monitoring* ... 68
13.1.3 *Insulin pump data* ... 68
13.2 QUESTIONNAIRES AND INTERVIEWS ... 68
13.2.1 *Questionnaires* ... 68
13.2.2 *Qualitative Interview* ... 68
13.3 LABORATORY METHODS ... 69
13.3.1 *Baseline Sample* ... 69
13.3.2 *Total Blood Loss* ... 69
13.4 ASSESSMENT OF SAFETY ... 69
13.5 ASSESSMENT OF EFFICACY ... 69
14 STUDY MATERIALS ... 70
14.1 STUDY PRODUCTS ... **ERROR! BOOKMARK NOT DEFINED.**
14.1.1 *Insulin* ... 70
14.1.2 *Study insulin pump* ... 70
14.1.3 *Study continuous subcutaneous glucose monitor* ... 70
14.1.4 *Computer-based algorithm* ... 70
14.1.5 *Conventional insulin pump treatment* ... 70
14.1.6 *Glucometer* ... 70
15 DATA ANALYSIS ... 70
15.1 PRIMARY ANALYSIS ... 70
15.2 SECONDARY ANALYSIS ... 70
15.3 INTERIM ANALYSIS ... 70
15.4 STATISTICAL METHODS ... 70
15.5 EXPLORATORY ANALYSIS ... 71
15.6 SAMPLE SIZE AND POWER CALCULATIONS ... 72
15.7 DEVIATIONS FROM THE STATISTICAL PLAN ... 72
16 CASE REPORT FORMS ... 73
17 DATA MANAGEMENT ... 73
18 STUDY MANAGEMENT ... 74
18.1 DATA MONITORING AND ETHICS COMMITTEE ... 74

18.2 STUDY MANAGEMENT COMMITTEE 74
18.3 STUDY MONITORING 74
19 RESPONSIBILITIES 75
19.1 CHIEF INVESTIGATOR 75
19.2 PRINCIPAL CLINICAL INVESTIGATORS 75
19.3 STUDY COORDINATOR 75
20 ETHICS 75
20.1 INDEPENDENT ETHICS COMMITTEES 75
20.2 INFORMED CONSENT OF STUDY SUBJECTS 76
21 TIMETABLE 76
22 DEVIATIONS FROM PROTOCOL 76
23 REPORTS AND PUBLICATIONS 76
24 RETENTION OF STUDY DOCUMENTATION 77
25 INDEMNITY STATEMENTS 77
26 APPENDICES AND TABLES 78
26.1 APPENDIX 1 78
26.2 APPENDIX 2 79
26.3 APPENDIX 3 81
26.4 APPENDIX 4 84
26.5 APPENDIX 5 85
26.6 APPENDIX 6 87
26.7 APPENDIX 7 – ADDQOL QUESTIONNAIRE 99
26.8 APPENDIX 8 – COMPETENCY ASSESSMENT FORMS 105
26.8.1 CGM Competency Assessment Form 105
26.8.2 Study pump Competency Assessment Form 106
26.8.3 Closed-loop Competency Assessment Form 108
27 REFERENCES 110

4 Summary

The main objective of this study is to determine whether day and night closed-loop insulin delivery under free living conditions is superior to addition of continuous glucose monitoring in adults with type 1 diabetes on insulin pump therapy with HbA1c between 7.5% and 10% (phase 1) and below 7.5% (phase 2).

This is an open-label, multi centre, randomised, crossover design study, involving a run-in period followed by two study periods during which glucose levels will be controlled either by an automated closed-loop system or by subjects usual insulin pump therapy with continuous glucose monitoring in random order. A total of up to 42 (phase 1) and 34 (phase 2) adults (aiming for 30 and 24 completed subjects in phases 1 and 2 respectively) aged 18 years and older with T1D on insulin pump therapy will be recruited through diabetes clinics and other established methods in participating centres. Subjects who drop out of the study within the first 6 weeks (phase 1) and 4 weeks (phase 2) of the first intervention arm will be replaced.

Subjects will receive appropriate training in the safe use of closed-loop insulin delivery system. Subjects will have regular contact with the study team during the home study phase including 24/7 telephone support. Subjects will be discouraged from international travel during the first two weeks of closed-loop use.

The primary outcome is time spent in target range between 3.9 and 10.0 mmol/L as recorded by CGM during home stay. Secondary outcomes are the HbA1c, time spent with glucose levels above and below target, as recorded by CGM, and other CGM-based metrics.

5 Background

Type 1 diabetes mellitus (T1D) is characterised by an absolute deficiency of insulin caused by immunologically-mediated damage to the beta cells in the pancreas and raised blood glucose levels. It is one of the commonest endocrine and metabolic conditions in both children and adults. It is estimated that approximately 285 million adults (5-15% type 1 diabetes) and 480,000 children (95% type 1 diabetes) worldwide suffer from diabetes. (1) Recent reports suggest that incidence and prevalence of T1D is increasing in many countries, at least in the under 15 year age group with the predicted number of new cases of childhood diabetes in Europe increasing to 24 400 in 2020 from 15 000 in 2005 (2; 3). This younger age at onset means that complications appear at a younger age, and dependence on lifelong insulin imposes a heavy burden on patients as well as health services. A survey conducted by Royal College of Paediatrics & Child Health in UK established that there were 22,783 children and young people 0-17 years with diabetes in England on 1st January 2009 out of which 97% had type 1 diabetes. (4)

Until the introduction of insulin replacement therapy in early 1920s T1D was a uniformly fatal condition. While significant advancements have been made in insulin therapy since, major limitations still exist, hypoglycaemia (low blood glucose) being the most significant. Despite the availability of therapeutic options such as self-monitoring of blood glucose, structured patient education, rapid-acting insulin analogues and insulin pump therapy, glycaemic control in the majority of patients with type 1 diabetes remains suboptimal and they are prone to get complications associated with poor control such as kidney failure and blindness. Risk of these complications can be reduced by intensive insulin therapy (5) but for most patients this is associated with hypoglycaemia (too low blood sugar) limiting the intensification of treatment. (6; 7) The average patient with T1D suffers two symptomatic episodes of hypoglycaemia per week, and one episode of severe hypoglycaemia, defined as an event requiring assistance of another person to administer rescue treatment in the form of carbohydrate and/or glucagon, per year. (8) Even in patients with good control, as judged by average HbA1c, significant glucose excursions occur with periods of silent hyper- and hypoglycaemia.(9; 10). Further patients with T1D have impaired defence mechanisms (counter-regulatory responses) to low glucose, thus impairing recovery and increasing the threat of future episodes.(11) Recurrent episodes may lead to hypoglycaemic unawareness, a condition which increases the risk of severe hypoglycaemia. (12) In addition to the physical morbidity,

hypoglycaemia also has significant psychological consequences including fear of future episodes with resulting maladaptive coping behaviours such as excessive eating or under-insulinising that may negatively impact glycaemic control.(13)

Despite the rapid advancements in insulin pump technology and the ongoing development of more physiological insulin preparations, the currently available therapeutic regimens are still unable to achieve optimal glycaemic control. The emergence of continuous glucose monitoring (CGM) over the last decade, which enables users to view real-time interstitial glucose readings and receive alarms for impending hypo- or hyperglycaemia, thus facilitating appropriate changes in insulin therapy, is a major step towards improved diabetes monitoring. Several recent studies have shown a clinical benefit of CGM on reduction in HbA1c, in those patients that are compliant with using the device.(14-17)

The development of a closed-loop system that combines glucose monitoring with computer-based algorithm dictated insulin delivery, may provide further improvements in glycaemic control while reducing hypoglycaemia and ultimately represent a realistic treatment option for people with T1D. The vital component of such a system, also known as an artificial pancreas (AP), is a computer-based algorithm. The role of the control algorithm is to translate, in real-time, the information it receives from the CGM and to compute the amount of insulin to be delivered by the pump. The other components include a real-time continuous glucose monitor and an infusion pump to titrate and deliver insulin.(18)

5.1 Closed-Loop Research in Cambridge, UK

Researchers at University of Cambridge, UK, led by Dr Hovorka, have been involved in developing state of the art Model Predictive Controller (MPC) computer algorithms and research into closed-loop insulin delivery since late 1990s. (19-27) The MPC approach can handle delays associated with insulin absorption and take into account meal intake and prandial boluses delivered manually by the patient. The model representation enables simulation of 'what if' scenarios, in particular the prediction of future glucose excursions resulting from projected and past insulin infusion rates.

Overnight closed-loop research conducted in Cambridge, UK has shown significantly better performance with closed-loop insulin delivery compared to usual pump therapy in both children and adults.(19; 20) Meta-analysis of these studies in 17 children and adolescents [13.4 ± 3.6 years] and 24 adults (37.5 ± 9.1 years) on 45 closed-loop (intervention) and 45 usual treatment (insulin pump therapy) visits, show closed-loop

increased the time in target plasma glucose in both young (from 40% to 60%, p = .002) and adults (from 50% to 76%, p < .001) compared with conventional insulin pump therapy.(22) Additionally, closed-loop reduced the time spent below 3.91 mmol/L and above 8.0 mmol/L, from 4.1% to 2.1% (p = .01) and 33% to 20% (p = .03), respectively. Glycaemic variability, as measured by the SD of plasma glucose, was lower during closed-loop compared with CSII (1.5 versus 2.1 mmol/liter, p = .007).

Further CL research in adolescence, involving 36 hours of day and night CL has resulted in significantly better time in target (84% vs. 49%) compared to usual pump therapy.(28)

5.2 AP@home consortium

In February 2010, the European Union granted funding for the AP@home project (Artificial Pancreas at Home), a consortium of European academic medical centres, biotechnology companies and industrial partners, to carry out closed-loop glucose control research. The ultimate goal of the AP@home project is to develop a closed-loop system for use outside clinical research centres. The first major clinical study under AP@home consortium was conducted in 6 European academic centres in 2011 (Appendix 1) and involved testing of closed-loop insulin delivery using two different algorithms in adults for 24 hours in the clinical research facility. Results of this study show significant improvements in hypoglycaemia rates (29) but there was no difference in the time spent target range between algorithm visits and standard treatment signifying the challenges of day time closed-loop control and need for further studies (Appendix 2).

5.3 Manual operational mode

The majority of the previous studies conducted in Cambridge, UK were conducted in the manual operational mode. (19; 20) This involved manual input of sensor glucose values into the computer-based algorithm, and manual adjustment of the insulin pump following algorithm generated insulin infusion rate advice.

5.4 Automated closed-loop mode

The next step was progression from a manual operational mode to an automated closed-loop insulin delivery system with wireless data transmission. Such a system (consisting of Cozmo insulin pump, Freestyle Navigator and Navigator Cradle – Appendix 3 and reference (21) was first evaluated in 8 young children, demonstrating

feasibility and similar efficacy with initiation of automated closed-loop operation at 18:00 versus 21:00. On both study nights, MPC algorithm communicated with the Navigator CGM device (via the Navigator Cradle) in order to acquire real-time glucose concentration values at one minute intervals. The advised insulin infusion rate was then sent to the Cozmo pump via wireless link and the pump infusion rate adjusted automatically. (21) This study was conducted after receiving notice of no objection from the United Kingdom, Medicines & Healthcare Products Regulatory Agency (MHRA) (Ref: CI/2008/0036).

Subsequently, in collaboration with Abbott Diabetes Care (CA, USA), further developments were undertaken, and a system known as “Florence” and “FlorenceD” (Figure 4) was developed in Cambridge, UK. These systems also received notice of no objection from the United Kingdom, Medicines & Healthcare products Regulatory Agency (MHRA Ref - CI/2008/0036). A further study was undertaken to assess the safety and reliability of this Florence closed-loop system and results showed good glucose control and system reliability (30).

5.5 Moving from Clinical research facility to home environment: Cambridge Home studies

Following successful demonstration of safety and efficacy of closed-loop insulin delivery in the research facility, overnight closed-loop studies under free living conditions were commenced in July 2012. The first study involving 16 adolescents using overnight closed-loop at home for 21 days has been completed recently (**REC Ref 10/H0304/87**). Preliminary results from this study show significantly better overnight glucose control using closed-loop insulin delivery. (Appendix 5). Further multi centre studies in adults comparing overnight (Cambridge, London and Sheffield, REC Ref **12/EE/0034)** closed loop glucose control are under-way.

5.6 Day and night closed loop insulin delivery under free living conditions using FlorenceD system

In January 2013, for the first time, we started evaluating the safety and efficacy of day and night closed loop glucose control under free living conditions in adults with type 1 diabetes (REC Ref **12/EE/0424)**. This feasibility study of 7 days duration adopted a classical cross over design and is currently in progress in 3 European countries (UK, Germany and Austria). By May 2013 9 subjects (50%) have completed the study and early results show improved glucose control. FlorenceD system received notice of no objection from regulatory authorities in UK, Germany and Austria for this study.

5.7 Automated closed-loop system to be used in the present study

In order to increase the portability and usability and to perform longer duration home studies further improvements have been made to the closed loop system. Figure 1 shows a prototype of the next generation FlorenceD2A automated closed-loop system to be used in the present study. FlorenceD2A is a purpose-built system, including a smartphone or similar mobile computer device containing the algorithm and communicating wirelessly with the CGM device and insulin pump. The system comprises of FreeStyle Navigator® II (Abbott Diabetes Care, Alameda, CA, USA), or similar Continuous Glucose Monitoring (CGM) System, Dana R Diabecare (Sooil Corp. Seoul, South Korea) or similar subcutaneous insulin infusion pump, and MPC-based glucose control algorithm running on a mobile computer device or smartphone.

The use of FlorenceD2A system in the current study will be subject to notice of no objection from the Medicine and Healthcare products Regulatory Agency (MHRA) in UK and relevant authorities in Germany and Austria.

Figure 1: Design of FlorenceD2A automated closed-loop system

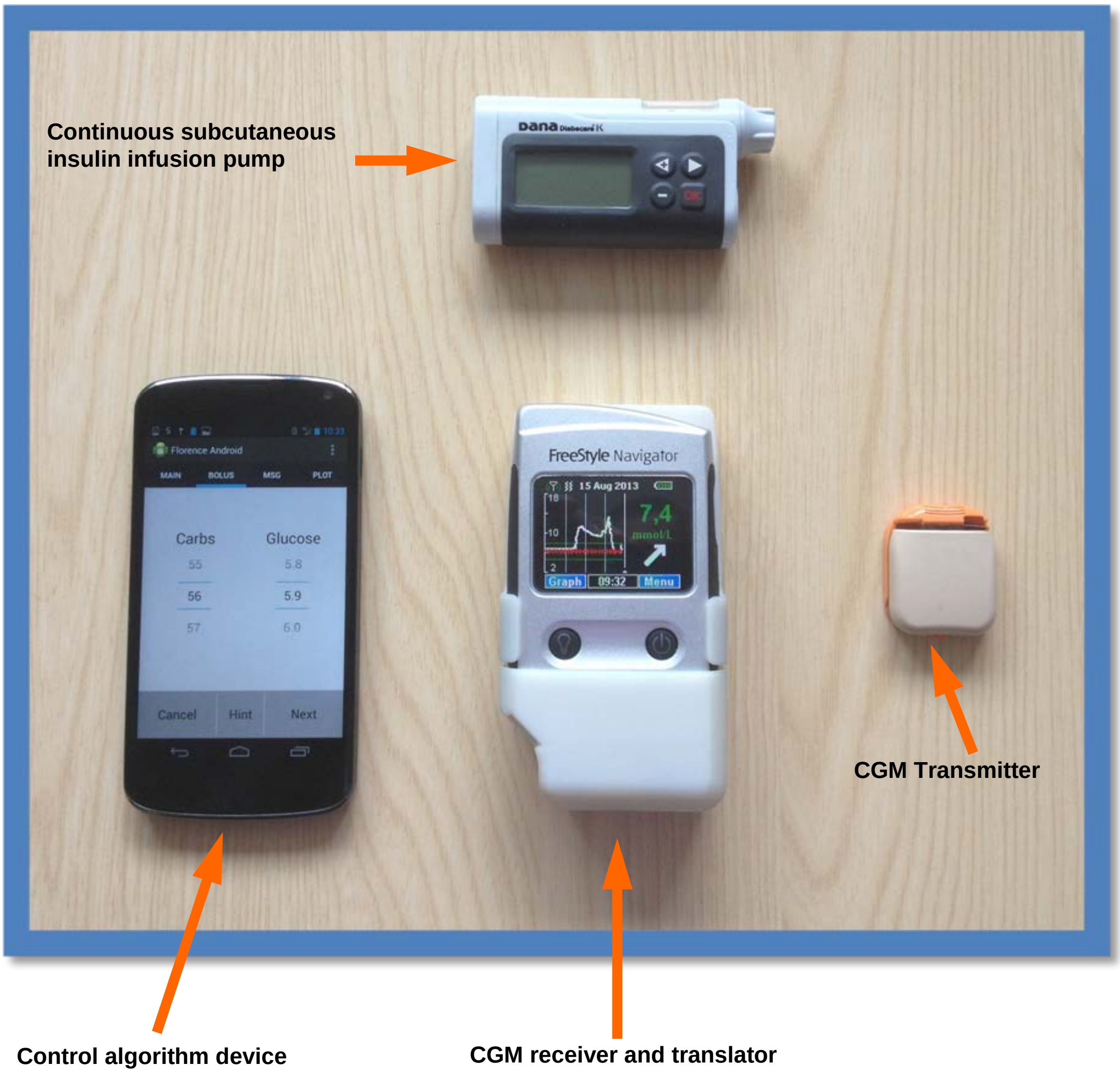

6 Objectives

6.1 Efficacy

To assess efficacy of automated closed-loop insulin delivery at home in maintaining glucose levels within the target range from 3.9 to 10.0 mmol/l based on subcutaneous continuous glucose monitoring (CGM) as compared to subcutaneous insulin infusion pump therapy combined with CGM.

6.2 Safety

To evaluate the safety of automated closed-loop glucose control in terms of episodes and severity of hypoglycaemia and nature and severity of other adverse events.

6.3 Utility

To determine the percentage of time when closed-loop was operational, and usability and acceptance of the closed-loop system.

7 Study Design

An open-label, multi-centre randomised, two-period crossover study comparing automated closed-loop glucose control with subcutaneous insulin infusion pump therapy combined with CGM.

8 Study Subjects

8.1 Study population

Adults aged 18 years or over with type 1 diabetes on insulin pump therapy will be recruited.

8.2 Recruitment and informed consent

The study will aim for 30 completed subjects (10 subjects per centre) for phase 1 and 24 completed subjects for phase 2. Recruitment will target up to 42 and 34 subjects for phase 1 and phase 2 respectively to allow for drop-outs. Methods of patient recruitment will follow well established practice at each centre.

1. UK - Participant recruitment will take place at the Wolfson Diabetes and Endocrine Clinic, Cambridge University Hospitals NHS Foundation Trust, Addenbrooke's Hospital, Cambridge, UK. Potential participants will be sent the study information leaflets and an invitation to join the study by the research team at least one week before the recruitment visit.
2. Germany – The recruitment of study participants is carried out by using a subject database. Subjects will be contacted by telephone and invited to attend the CRC for further explanation of the study including provision of the participant information sheet
3. Austria – Subject will be contacted by telephone and invited to attend the CRC for further explanation of the study including provision of the participant information sheet.

Written informed consent will be obtained from all participants before any study related activities.

8.2.1 Inclusion criteria

1. The subject has type 1 diabetes as defined by WHO
2. The subject is 18 years of age or older
3. The subject will have been on an insulin pump for at least 6 months with good knowledge of insulin self-adjustment including carbohydrate counting
4. The subject is treated with one of the rapid acting insulin analogues (Insulin Aspart, Insulin Lispro or Insulin Glulisine)
5. HbA1c 7.5-10% (59-86 mmol/mol) for phase 1 and <7.5% (59 mmol/mol) for phase 2, based on analysis from central laboratory or equivalent
6. The subject is willing to perform regular finger-prick blood glucose monitoring, with at least 6 measurements per day
7. The subject is willing to wear closed-loop system at home and at work place
8. The subject is willing to follow study specific instructions
9. The subject is willing to upload pump and CGM data at regular intervals
10. The subject is willing to restrict alcohol consumption to ≤ 2 units per day throughout the study period
11. Female subjects of child bearing age should be on effective contraception and must have a negative urine-HCG pregnancy test at screening. In addition in Germany, women of childbearing potential must use a highly effective method of birth control, which is defined as those which result in a low failure rate (i.e. less than 1% per year) and must use two independent methods of contraception, e.g. diaphragm and spermicide-coated condom.

12. The subject has access to WiFi (phase 2 only)

8.2.2 Exclusion criteria for all countries

1. Non-type 1 diabetes mellitus
2. Subjects who are living alone
3. Any other physical or psychological disease or condition likely to interfere with the normal conduct of the study and interpretation of the study results
4. Current treatment with drugs known to have significant interference with glucose metabolism, such as systemic corticosteroids, as judged by the investigator
5. Known or suspected allergy against insulin
6. Subjects with clinically significant nephropathy (eGFR < 45ml/min), neuropathy or active retinopathy (defined as presence of maculopathy or more than background diabetic retinopathy changes) as judged by the investigator
7. Significantly reduced hypoglycaemia awareness (Gold score ≥ 4 according to Geddes J et al, Diabetes Care 2007)
8. More than one episode of severe hypoglycaemia as defined by American Diabetes Association (31) in preceding 12 months (Severe hypoglycaemia is defined as an event requiring assistance of another person to actively administer carbohydrates, glucagon, or take other corrective actions including episodes of hypoglycaemia severe enough to cause unconsciousness, seizures or attendance at hospital.)
9. Random C-peptide > 100pmol/l with concomitant plasma glucose >4 mM(72 mg/dl)
10. Total daily insulin dose ≥ 2 IU/kg/day
11. Subject is pregnant or breast feeding or planning pregnancy within next 10 months
12. Severe visual impairment
13. Severe hearing impairment
14. Subjects using implanted internal pacemaker
15. Lack of reliable telephone facility for contact
16. Subject not proficient in English (UK) or German (Germany and Austria)

8.2.3 Additional exclusion criteria specific for Austria and Germany

1. Positive results on urine drug screen (amphetamines/metamphetamines, barbiturates, benzodiazepines, cannabinoids, cocaine, opiates).
2. Positive alcohol breath test.

3. Positive reaction to any of the following tests: hepatitis B surface (HBs) antigen, anti-hepatitis C virus (anti-HCV) antibodies, anti-human immunodeficiency virus (HIV) 1 antibodies, anti-HIV2 antibodies.

8.2.4 Additional exclusion criteria specific for Germany only

1. Serious macro- and microangiopathy
2. Serious anomalies of the skin
3. Serious skin diseases (e.g. psoriasis vulgaris, bacterial skin diseases) located at places of the body, which potentially are possible to be used for localisation of the glucose sensor)
4. Renal insufficiency
5. Epilepsy
6. Eating disorders (like bulimia or anorexia nervosa)
7. Disorders of the lipid metabolism
8. Blood transfusion requiring patients
9. Psychiatric diseases and related conditions
10. Patients with frequent catheter abscesses having occurred in connection with the pump therapy
11. Patients with medically documented allergy towards the adhesive (glue) of plasters
12. Abnormal blood values for:
    - the creatinine clearance,
    - erythropoietin,
    - TSH.
13. Patients with the following concomitant medications or misuse of substances:
    - steroids,
    - anticoagulant therapies.
14. Patients with a planned intervention under general anaesthesia.
15. Patients who do shift work

8.3 Randomisation

The order or closed-loop and usual insulin pump therapy will be randomly allocated using a centrally administered randomisation programme. Randomisation will take place 24 to 48 hours before the start of first home study phase.

Figure 2. Study flow chart

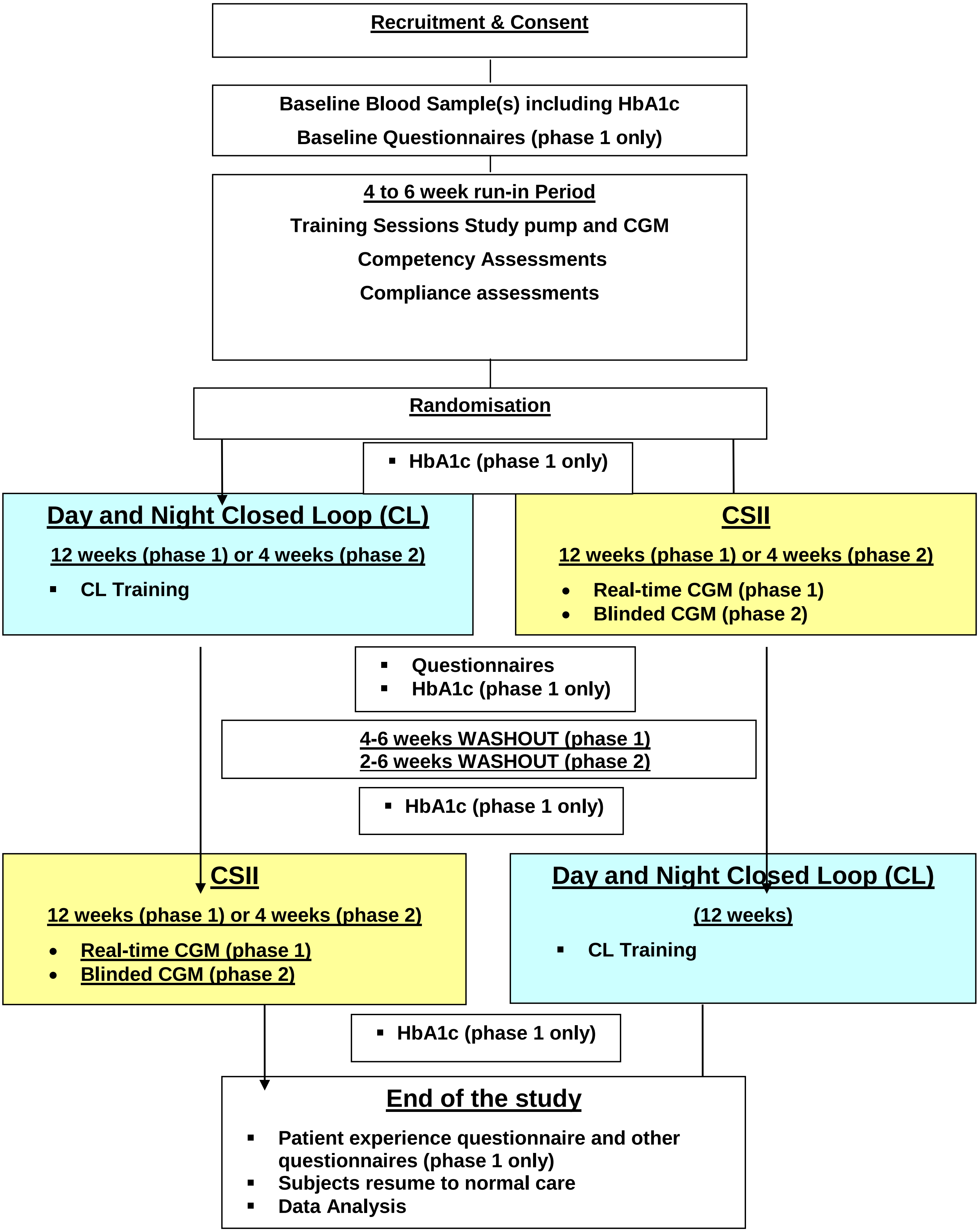

9 Methods under Investigation

9.1 Name and description of the method of investigation

The investigational treatment is the automated closed-loop insulin delivery system as described in section 5.7 manufactured by the Cambridge University Hospitals NHS Foundation Trust. Component versions will be identified during regulatory submission to the national regulatory bodies.

9.2 Intended purpose

The intended purpose of the investigational treatment is closed-loop insulin delivery during day-time and night-time.

9.3 Method of administration

The closed-loop system consists of components directly attached to the subject, which are the CGM transmitter and the insulin pump. The components not directly attached to the subject comprise (i) mobile computer device or smartphone that contains the closed-loop algorithm and (ii) CGM receiver (Figure 1).

9.4 Required training

All participants will be trained to use the study CGM device and insulin pump at the start of run-in period. During the first 4-6 hours of the study period for closed-loop glucose control in the clinical research facility, subjects will be provided with required training to operate the closed-loop system independently at home. Particular attention will be paid to starting and stopping the system and reinstating subjects' usual insulin therapy if any problems occur at home. Competency assessment will be undertaken before subject is allowed home.

9.5 Precautions

During treatment with insulin there is a risk of hypoglycaemia and hyperglycaemia. In-hospital testing and hazard analysis, have documented reduced risk of hypoglycaemia and hyperglycaemia during closed-loop compared to conventional treatment.

9.6 Accountability of the method under investigation

The investigator will provide training for the study participants and will make every effort, through regular contact, to ascertain that the closed-loop system is used for the study purposes only.

10Study Schedule (phase 1)

10.1 Overview

The study will be coordinated from the Institute of Metabolic Science, University of Cambridge, UK. The study will consist of up to 20 visits or pre-planned telephone/email contacts including two study periods (closed-loop vs. conventional pump therapy + CGM). The study periods will last 12 weeks each. The order of the two interventions will be in random order.

The first 4-6 hours of the closed loop period will be conducted at the CRC of the respective centre. Two 12 week study periods will be performed within four to six weeks of each other.

Table 1 outlines study activities in phase 1 when CL intervention precedes conventional pump therapy with CGM.
Table 2 outlines study activities in phase 1 when conventional pump therapy with CGM precedes CL intervention.

Table 1. Schedule of study visits in phase 1 when closed-loop intervention precedes conventional pump therapy.

	Visit/ contact	Description	Start relative to previous / next Visit / Activity	Duration
	Visit 1	Recruitment visit: Consent<br>HbA1c, baseline bloods<br>Urine pregnancy test	-	1-3 hours
Training	Visit 2	Insulin pump training and the initiation study pump<br>Competency assessment	Within 1 to 3 weeks of Visit 1	3-4 hours
	Visit 3	CGM training<br>Initiation of study CGM<br>Competency assessment	Within 3 to 7 days of Visit 2	2-3 hours
Optimisation (4 weeks)	Visit 4	Review pump and CGM data & optimisation of treatment. Further device training as required	After 1 week of Visit 3	1 hour
	Visit 5	Review pump and CGM data & optimisation of treatment	After 1 week of Visit 4	1 hour
	Visit 6	Review pump and CGM data & optimisation of treatment	After 1 week of Visit 5	1 hour
	Visit 7	Review pump and CGM data & optimisation of treatment	After 1 week of Visit 6	1 hour
CL Intervention (3 months)	Visit 8	CL initiation at CRC<br>- urine pregnancy test<br>- CL training<br>- competency assessment<br>- HbA1c	Within 1 week of Visit 7	6 hours
	*Visit 9	Review use of study devices; further optimisation as required	After 1 week of Visit 8	<1 hour
	*Visit 10	Review pump and CGM data; further optimisation as required	After 1 week of visit 9	<1 hour
	*Visit 11	End of first month; Review pump and CGM data; further optimisation as required	After 2 weeks of visit 10	<1 hour
	*Visit 12	End of second month; Review pump and CGM data; further optimisation as required	After 4 weeks of visit 11	<1 hour

	Visit 13	End of closed-loop treatment arm (3 months) HbA1c. Complete questionnaires and optional interviews.<br>Collect algorithm device (Optional return of CGM and study pump)	After 4 weeks of visit 12	1-2 hours
	-	Washout period (patient may use study pump and CGM)	Immediately after Visit 13	4 to 6 weeks
	Visit 14	Optional - Collection of CGM and study pump if handed back during visit 13	At least 1 week before visit 16	< 1 hour
**OL Intervention (3 months)**	Visit 15	OL initiation at CRC<br>- urine pregnancy test<br>- OL training<br>- competency assessment<br>- HbA1c	Within 4 to 6 week of end of Visit 13	Up to 6 hours
	*Visit 16	Review use of study devices; further optimisation as required	After 1 week of Visit 15	<1 hour
	*Visit 17	Review pump and CGM data; further optimisation as required	After 1 week of visit 16	<1 hour
	*Visit 18	End of first month; Review pump and CGM data; further optimisation as required	After 2 weeks of visit 17	<1 hour
	*Visit 19	End of second month; Review pump and CGM data; further optimisation as required	After 4 weeks of visit 18	<1 hour
	Visit 20	End of open-loop treatment arm (3 months) HbA1c. Complete questionnaires and optional interviews.<br>Collect all study devices	After 4 weeks of visit 19	1-2 hours

***Could be done via phone / e-mail**

Table 2. **Schedule of study visits in phase 1 when conventional pump therapy precedes closed-loop intervention.**

	Visit/ contact	Description	Start relative to previous / next Visit / Activity	Duration
	Visit 1	Recruitment visit: Consent HbA1c, baseline bloods Urine pregnancy test	-	1-3 hours
**Training**	Visit 2	Insulin pump training and the initiation study pump Competency assessment	Within 1 to 3 weeks of Visit 1	3-4 hours
	Visit 3	CGM training Initiation of study CGM Competency assessment	Within 3 to 7 days of Visit 2	2-3 hours
**Optimisation (4 weeks)**	Visit 4	Review pump and CGM data & optimisation of treatment. Further device training as required	After 1 week of Visit 3	1 hour
	Visit 5	Review pump and CGM data & optimisation of treatment	After 1 week of Visit 4	1 hour
	Visit 6	Review pump and CGM data & optimisation of treatment	After 1 week of Visit 5	1 hour
	Visit 7	Review pump and CGM data & optimisation of treatment	After 1 week of Visit 6	1 hour
**OL Intervention (3 months)**	Visit 15	OL initiation at CRC<br>- urine pregnancy test<br>- OL training<br>- competency assessment<br>- HbA1c	Within 1 week of Visit 7	Up to 6 hours
	*Visit 16	Review use of study devices; further optimisation as required	After 1 week of Visit 15	<1 hour
	*Visit 17	Review pump and CGM data; further optimisation as required (Could be done via phone / e-mail)	After 1 week of visit 16	<1 hour
	*Visit 18	End of first month; Review pump and CGM data; further optimisation as required	After 2 weeks of visit 17	<1 hour
	*Visit 19	End of second month; Review pump and CGM data; further optimisation as required	After 4 weeks of visit 18	<1 hour

	Visit 20	End of open-loop treatment arm (3 months) HbA1c. Complete questionnaires and optional interviews. Optional return of CGM and study pump	After 4 weeks of visit 19	1-2 hours
	-	Washout period (patient may use study pump and CGM)	Immediately after Visit 20	4 to 6 weeks
	Visit 14	Optional - Collection of devices if handed back during visit 20 (CGM, study pump)	At least 1 week before visit 8	< 1 hour
**CL Intervention (3 months)**	Visit 8	CL initiation at CRC<br>- urine pregnancy test<br>- CL training<br>- competency assessment<br>- HbA1c	Within 4 to 6 week of end of Visit 20	6 hours
	*Visit 9	Review use of study devices; further optimisation as required	After 1 week of Visit 8	<1 hour
	*Visit 10	Review pump and CGM data; further optimisation as required (Could be done via phone / e-mail)	After 1 week of visit 9	<1 hour
	*Visit 11	End of first month; Review pump and CGM data; further optimisation as required	After 2 weeks of visit 10	<1 hour
	*Visit 12	End of second month; Review pump and CGM data; further optimisation as required	After 4 weeks of visit 11	<1 hour
	Visit 13	End of closed-loop treatment arm (3 months) HbA1c. Complete questionnaires and optional interviews	After 4 weeks of visit 12	1-2 hours

<u>*Could be done via phone / e-mail</u>

10.2 Recruitment Visit (Visit 1)

The recruitment visit will be tailored to meet local requirements

- UK - At this visit, participants will be given a verbal explanation of the study, and will have the opportunity to ask any questions. If they are interested in proceeding with the study, and providing they meet the inclusion/exclusion criteria, written informed consent will be obtained by a competent member of the research team. Those who would prefer to have more time to consider their decision to participate will have the option of returning to the clinic at a later date to complete the screening visit and give consent. At the screening visit, subject's height, weight, demographics, medical and diabetes history, concomitant medications and their previous day's self-monitored glucose levels and insulin therapy will be recorded. During this visit subjects will have a blood test to determine their C-Peptide, glucose, full blood count, HbA1c, renal function, thyroid functions, coeliac screen and liver functions. A urine pregnancy test will be done for all females of child bearing age.
- Germany - At the recruitment visit, participants will be given written information and a verbal explanation of the study, and they will have the opportunity to ask any questions. If they are interested in proceeding with the study, written informed consent will be obtained by a research physician. At the screening visit, subject's height, weight, demographics, medical and diabetes history, concomitant medications and their previous day's self-monitored glucose levels and insulin therapy will be recorded. During this visit the subject will have a breath alcohol test, a urine drug screen, and blood tests to determine their C-Peptide, glucose full blood count, HbA1c, renal function (serum creatinine, creatinine clearance), liver function (ALT, AST), urea, sodium, potassium, coagulation, serology (HIV/Hepatitis B, C) TSH, and erythropoietin. A urine pregnancy test will be done for all females. Based on the anamnestic data and the test results inclusion and exclusion criteria are checked.
- Austria - At the recruitment visit, participants will be given written information and a verbal explanation of the study, and they will have the opportunity to ask any questions. If they are interested in proceeding with the study, written informed consent will be obtained by a physician. At the screening visit, subject's height, weight, demographics, medical and diabetes history, concomitant medications and their previous day's self-monitored glucose levels and insulin therapy will be recorded. During this visit the subject will

have a breath alcohol test, a urine drug screen, and blood tests to determine their C-Peptide, glucose full blood count, HbA1c, renal function (serum creatinine), liver function (ALT, AST), urea, sodium, potassium, coagulation, serology (HIV/Hepatitis B, C). A urine pregnancy test will be done for all females. Based on the anamnestic data and the test results inclusion and exclusion criteria are checked.

10.3 Visit 2 - Training session on the use of the study insulin pump

This session will cover key aspects of insulin pump use and particular attention will be paid to the following areas. Written easy to use guidelines for the operation of insulin pump will be provided. This session will be conducted by a professional pump educator ± member of the study team following a written curriculum. Competency on the use of study pump will be made.

- Importance of carbohydrate counting and refresher on carbohydrate counting skills
- Understanding insulin to carb ratios and correction factor
- Correct use of bolus calculator - subjects will be required to use this bolus calculator for all insulin boluses during the study period.
- Insulin cartridge and Infusion set changes and correct priming procedure
- Sick day rules
- Dealing with hypo and hyperglycaemia
- Uploading pump data using software

10.4 Visit 3 - Training session on the continuous glucose monitoring (CGM) and study pump

This session will cover key aspects of the study CGM device and particular attention will be paid to the following areas. Written easy to use guidelines for the operation of CGM device will be provided. This session will be conducted by a professional pump educator ± member of the study team following a written curriculum. Competency on the use of CGM will be made.

- Insertion and initiation of sensor session
- Use of handheld CGM receiver & sensor calibrations

- Use of software to analyse CGM data
- Use of CGM data to optimise treatment
- Uploading CGM data using software

10.5 Visit 4 – Further device training, review of data and start of optimisation period

The subject will be invited to attend the research centre approximately 1 week after visit 3. The purpose of this visit will be to provide any additional device training required and to start treatment optimisation. Insulin pump and CGM device will be downloaded and the data will be utilised for optimisation of treatment. Particular attention will be paid to avoiding hypoglycaemia in the post prandial period by optimising meal bolus.

10.6 Visits 5, 6 and 7 – Treatment Optimisation period

The subject will be invited to attend the research centre at weekly intervals during this period. Study insulin pump and CGM device will be downloaded and data will be used for treatment optimisations. There should be a minimum of 4 weeks optimisation period for all subjects. (End of visit 3 to end of visit 7). Optimisation period will be conducted by a professional pump educator ± member of the study team following a written curriculum.

During visit 7, subject's compliance of using the study CGM and study pump over preceding 14 days will be assessed. To proceed with the study subject needs to demonstrate correct use of study insulin pump including use of bolus calculator over 90% of meal boluses and at least 10 days' worth of CGM data during last 14 days of optimisation period. In addition, subject should carry out upload of CGM and pump data at weekly intervals. If subject fails to demonstrate compliance, the study will be terminated and subject will be removed from the study.

Subject randomisation for the order of closed-loop intervention and conventional pump therapy will take place during the week following Visit 7.

Maximum period allowed between Visit 3 and Visits 8 or 15 (Training and optimisation) is six weeks with a minimum of four weeks. Between visit 3 and visit 8, subject will be invited for a qualitative interview (face to face or telephone interview) at a mutually convenient time as described in section 18.2.2.

The next section describes the situation when closed-loop precedes the conventional pump therapy.

10.7 Visit 8 (Closed-loop visit; within 1 week of visit 7 and at least 4 weeks after visit 3)

Subject will arrive at the clinical research facility at 09.00.

Urine pregnancy test will be performed on arrival.

Between 09:00 and 10:00 am subjects will be provided with a training session on all aspects of the closed-loop system relevant for safe and effective use. This will include training on connection and disconnection of the closed-loop system and switching between closed-loop and usual pump therapy. Particular attention will be paid to meal bolus procedure and use of closed-loop treatment during exercise. Written step by step guidance will also be provided. Competency on the use of closed-loop system will be assessed by the study team. Only the subjects who demonstrate competency on use of the system will be allowed to continue to the home study phase.

Closed-loop glucose control will start from 10:00 hours.

Subjects will be provided with a snack containing 20g carbohydrate accompanied by meal bolus delivered from closed-loop system at 11:00 hours. Subjects will be given lunch (60g CHO) at 13:00 accompanied by meal bolus delivered from closed-loop system.

During this visit subject will have a blood test for HbA1c and will be asked to complete questionnaires as outlined in section 18.2.1

Subjects will be allowed to go home at approximately 15:00 hours and they may walk, use public transport, or will be provided with transport according to local practice in each centre. They will be advised to continue closed-loop at home over the next 3 months.

During the three months home treatment period, the subject will be asked to upload /download the study insulin pump and CGM device at 5 day to three week intervals (after each sensor insertion during closed loop to three weeks during open loop as

given by data storage capacity of Freestyle Navigator Receiver) for storage of data. In addition, subjects can also use software provided by the manufacturer of CGM device (Abbott Diabetes Care – CoPilot Software) for visualization of CGM data during home study period.

During the three months home closed-loop treatment, the subject is allowed to drive while adhering to usual precautions and country specific rules and regulations.
Subjects will be advised to discontinue automated closed-loop insulin delivery and follow their usual insulin pump therapy for periods of exercise in the first 2 weeks of closed-loop use. After first 2 weeks, in participants who usually undertake exercise, closed-loop system may be used during moderate exercise. Participants will be warned about hypoglycemia if they start regular exercise during study period. During the first 2 weeks of each study period subject will be advised against international travel. During rest of study period travel inside European Economic Area is allowed.

Subjects will be provided with 24 hour telephone helpline. Subjects will also be given written instructions about dealing with low and high glucose at home and when to contact study team.

Subjects will be asked to monitor finger stick glucose levels every 4 - 6 hours during waking hours. They will be required to contact the study team if they encounter any problems with closed-loop system.

10.8 Visit 9 – 1 week after starting CL treatment

The subject will be contacted via telephone/e-mail or invited to attend the research centre approximately 1 week after Visit 8. The purpose of this visit will be to review the use of closed-loop system and to provide any additional training required. Data downloaded will be utilised for optimisation of treatment. Particular attention will be paid to avoiding hypoglycaemia in the post prandial period by optimising meal bolus.

10.9 Visit 10 – 2 weeks after starting CL treatment

The subject will either be contacted via telephone/e-mail or seen in the clinic 1 week after visit 9. The purpose of this visit would be to troubleshoot any problems. The subject is free to optimise further treatment but no active treatment optimisation will be undertaken by the study team.

10.10 Visit 11 – End of first month on CL treatment

The subject will be contacted via telephone/e-mail or invited to attend the research centre approximately 1 month after start of CL treatment to review the use of study devices. Any additional device training required will be provided. The subject is free to optimise further treatment but no active treatment optimisation will be undertaken by the study team.

10.11 Visit 12 – End of second month on CL treatment

The subject will be contacted via telephone/e-mail or invited to attend the research centre approximately 1 month after visit 11 to review the use of study devices. The subject is free to optimise further treatment but no active treatment optimisation will be undertaken by the study team.

10.12 Visit 13 – End of CL treatment

The subject will be invited to attend the research centre approximately 1 month after visit 12. This would be the end of three months home CL use. Insulin pump and CGM device data will be downloaded. The subject will have a blood test for the HbA1c. Subject will be asked to complete questionnaires / interview as outlined in section 18.2.1 and 18.2.2. The control algorithm device will be collected. The subject may use the study CGM and study pump during the washout period.

10.13 Visit 14 (Optional)

The purpose of Visit 14 is to return study insulin pump and CGM (if collected during visit 13) to patient. Individual centres may use locally accepted practices (visiting subject's home / arranging a courier without having to ask the subject to come to CRC). This will minimise the burden and loss of work time for the subject.

10.14 Washout

Minimum washout period of four weeks (maximum 6 weeks) must be ensured between treatment arms. Duration of the wash out period is chosen to minimise carry

over effect between the two interventions. This duration was decided pragmatically based on experience of the study team as there are no published studies investigating an optimal washout period for closed-loop studies. Subject may continue to use the study CGM and study insulin pump during the washout period if he or she wishes. Otherwise subject will use usual insulin pump therapy (using own insulin pump) during this period. Then subjects will cross over to alternative intervention. The subject is required to use the study insulin pump and CGM for at least 1 week before the Visit 16.

10.15 Visit 15 (Control visit)

The order of Visit 8 and Visit 15 will be in random order. Visit 15 is similar to Visit 8 except that in Visit 15 there is no closed-loop insulin delivery and subjects will continue their usual insulin pump therapy with CGM with self-management as appropriate. Subjects will follow usual treatment guidelines for driving, exercise, hyperglycaemia and hypoglycaemia.

10.16 Visit 16 – 1 week after starting OL treatment

The subject will be contacted via telephone/e-mail or invited to attend the research centre approximately 1 week after Visit 15. The purpose of this visit will be to review the use of the study CGM and study insulin pump and to provide any additional training required. Data downloaded will be utilised for optimisation of treatment. Particular attention will be paid to avoiding hypoglycaemia in the post prandial period by optimising meal bolus.

10.17 Visit 17 – 2 weeks after starting OL treatment

The subject will either be contacted via telephone/e-mail or seen in the clinic 1 week after visit 16. The purpose of this visit would be to troubleshoot any problems. The subject is free to optimise further treatment but no active treatment optimisation will be undertaken by the study team.

10.18 Visit 18 – End of first month on OL treatment

The subject will be contacted via telephone/e-mail or invited to attend the research centre approximately 1 month after start of open loop treatment to review the use of

study devices. Any additional device training required will be provided. The subject is free to optimise further treatment but no active treatment optimisation will be undertaken by the study team.

10.19 Visit 19 – End of second month on OL treatment

The subject will be contacted via telephone/e-mail or invited to attend the research centre approximately 1 month after visit 18 to review the use of study devices. Any additional device training required will be provided. The subject is free to optimise further treatment but no active treatment optimisation will be undertaken by the study team.

10.20 Visit 20 – End of OL treatment

The subject will be invited to attend the research centre approximately 1 month after visit 19. This would be the end of three months OL treatment. Insulin pump and CGM device data will be downloaded. The subject will have a blood test for the HbA1c . Subject will be asked to complete questionnaires / interviews as outlined in section 18.2.1 and 18.2.2.

11 Study Schedule (phase 2)

11.1 Overview

The study will be coordinated from the Institute of Metabolic Science, University of Cambridge, UK. The study will consist of up to 10 visits or pre-planned telephone/email contacts including two study periods (closed-loop vs. conventional pump therapy + blinded CGM). The study periods will last 4 weeks each. The order of the two interventions will be in random order.

The first 4-6 hours of the closed loop period will be conducted at the CRC of the respective centre. Two 4 week study periods will be performed within two to four weeks of each other.

Table 3 outlines study activities in phase 2 when CL intervention precedes conventional pump therapy with CGM.
Table 4 outlines study activities in phase 2 when conventional pump therapy with CGM precedes CL intervention.

Table 3. Schedule of study visits in phase 2 when closed-loop intervention precedes conventional pump therapy.

	Visit/ Contact	Description	Start relative to previous / next Visit / Activity	Duration
	Visit 1	Recruitment visit: Consent HbA1c, baseline bloods Urine pregnancy test	-	1-3 hours
Training	Visit 2	Insulin pump training and the initiation study pump Competency assessment	Within 1 to 3 weeks of Visit 1	3-4 hours
	Visit 3	CGM training Initiation of study CGM Competency assessment	Within 3 to 7 days of Visit 2	2-3 hours
Run-in (2-4 weeks)	Visit 4	Review pump and CGM data & optimisation & compliance assessment, randomisation (24-48 hours prior to V5)	After 2-4 week of Visit 3	1 hour
CL Intervention (1 month)	Visit 5	CL initiation at CRC<br>- urine pregnancy test<br>- CL training<br>- competency assessment	Within 1 week of Visit 4	Up to 6 hours
	-	CL at home	Immediately after Visit 5	1 month
	Visit 6	End of closed-loop treatment arm (1 month). Collect algorithm device (Optional return of CGM and study pump). Complete participant experience questionnaire.	After 4 weeks of Visit 5	<1 hour
	-	Washout period (patient may use study pump and CGM)	Immediately after Visit 6	2-4 weeks
Phase 2 OL Intervention (1 month)	Visit 7	Optional - Collection of CGM and study pump if handed back during visit 6	2-4 weeks after Visit 6	< 1 hour
	Visit 8	OL initiation at CRC<br>- urine pregnancy test<br>- OL training<br>- competency assessment	2-4 weeks after Visit 6	Up to 6 hours
		OL at home	Immediately after Visit 8	1 month

	Visit 9	End of open-loop treatment arm (1 months)<br>Collect all study devices	After 4 weeks of Visit 8	1-2 hours

Table 4. Schedule of study visits in phase 2 when conventional pump therapy precedes closed-loop intervention

	Visit/ contact	Description	Start relative to previous / next Visit / Activity	Duration
	Visit 1	Recruitment visit: Consent HbA1c, baseline bloods Urine pregnancy test	-	1-3 hours
Training	Visit 2	Insulin pump training and the initiation study pump Competency assessment	Within 1 to 3 weeks of Visit 1	3-4 hours
	Visit 3	CGM training Initiation of study CGM Competency assessment	Within 3 to 7 days of Visit 2	2-3 hours
Run-in, (2-4 weeks)	Visit 4	Review pump and CGM data & optimisation & compliance assessment, randomisation (24-48 hours prior to V5).	After 2-4 week of Visit 3	1 hour
OL Intervention (1 month)	Visit 5	OL initiation at CRC<br>- urine pregnancy test<br>- OL training<br>- competency assessment	Within 1 week of Visit 4	Up to 6 hours
	-	OL at home	Immediately after Visit 5	1 month
	Visit 6	End of open-loop treatment arm (1 months)<br>Optional return of CGM and study pump.	4 weeks after Visit 5	1-2 hours
	-	Washout period (patient may use study pump and CGM)	Immediately after Visit 6	2-4 weeks
Phase 2 CL Intervention (1 month)	Visit 7	Optional - Collection of CGM and study pump if handed back during visit 6	2-4 weeks after Visit 6	< 1 hour
	Visit 8	CL initiation at CRC<br>- urine pregnancy test<br>- CL training<br>- competency assessment	2-4 weeks after Visit 6	6 hours
	-	CL at home	Immediately after Visit 8	1 month
	Visit 9	End of closed-loop treatment arm (1 month).<br>Collect all study devices.<br>Complete participant experience questionnaire.	After 4 weeks of Visit 8	<1 hour

11.2 Recruitment Visit (Visit 1)

The recruitment visit will be tailored to meet local requirements

- UK - At this visit, participants will be given a verbal explanation of the study, and will have the opportunity to ask any questions. If they are interested in proceeding with the study, and providing they meet the inclusion/exclusion criteria, written informed consent will be obtained by a competent member of the research team. Those who would prefer to have more time to consider their decision to participate will have the option of returning to the clinic at a later date to complete the screening visit and give consent. At the screening visit, subject's height, weight, demographics, medical and diabetes history, concomitant medications and their previous day's self-monitored glucose levels and insulin therapy will be recorded. During this visit subjects will have a blood test to determine their C-Peptide, glucose, full blood count, HbA1c, renal function, thyroid functions, coeliac screen and liver functions. A urine pregnancy test will be done for all females of child bearing age.
- Germany - At the recruitment visit, participants will be given written information and a verbal explanation of the study, and they will have the opportunity to ask any questions. If they are interested in proceeding with the study, written informed consent will be obtained by a research physician. At the screening visit, subject's height, weight, demographics, medical and diabetes history, concomitant medications and their previous day's self-monitored glucose levels and insulin therapy will be recorded. During this visit the subject will have a breath alcohol test, a urine drug screen, and blood tests to determine their C-Peptide, glucose full blood count, HbA1c, renal function (serum creatinine, creatinine clearance), liver function (ALT, AST), urea, sodium, potassium, coagulation, serology (HIV/Hepatitis B, C) TSH, and erythropoietin. A urine pregnancy test will be done for all females. Based on the anamnestic data and the test results inclusion and exclusion criteria are checked.
- Austria - At the recruitment visit, participants will be given written information and a verbal explanation of the study, and they will have the opportunity to ask any questions. If they are interested in proceeding with the study, written informed consent will be obtained by a physician. At the screening visit, subject's height, weight, demographics, medical and diabetes history, concomitant medications and their previous day's self-monitored glucose levels and insulin therapy will be recorded. During this visit the subject will have a breath alcohol test, a urine drug screen, and blood tests to determine

their C-Peptide, glucose full blood count, HbA1c, renal function (serum creatinine), liver function (ALT, AST), urea, sodium, potassium, coagulation, serology (HIV/Hepatitis B, C). A urine pregnancy test will be done for all females. Based on the anamnestic data and the test results inclusion and exclusion criteria are checked.

11.3 Visit 2 - Training session on the use of the study insulin pump

This session will cover key aspects of insulin pump use and particular attention will be paid to the following areas. Written easy to use guidelines for the operation of insulin pump will be provided. This session will be conducted by a professional pump educator ± member of the study team following a written curriculum. Competency on the use of study pump will be made.

- Importance of carbohydrate counting and refresher on carbohydrate counting skills
- Understanding insulin to carb ratios and correction factor
- Correct use of bolus calculator - subjects will be required to use this bolus calculator for all insulin boluses during the study period.
- Insulin cartridge and Infusion set changes and correct priming procedure
- Sick day rules
- Dealing with hypo and hyperglycaemia
- Uploading pump data using software

11.4 Visit 3 - Training session on the continuous glucose monitoring (CGM) and study pump

This session will cover key aspects of the study CGM device and particular attention will be paid to the following areas. Written easy to use guidelines for the operation of CGM device will be provided. This session will be conducted by a professional pump educator ± member of the study team following a written curriculum. Competency on the use of CGM will be made.

- Insertion and initiation of sensor session
- Use of handheld CGM receiver & sensor calibrations
- Use of software to analyse CGM data

- Use of CGM data to optimise treatment
- Uploading CGM data using software

11.5 Visit 4 –Review of pump and CGM data during run-in period & optimisation & compliance assessment & randomisation

Subjects will use study insulin pump and CGM during run-in period. There will be a minimum of two weeks run-in period for all subjects (end of Visit 3 to end of Visit 4). Subjects will be contacted once weekly during the run-in period via phone/email to troubleshoot any problems. Subjects will also be able to contact the research team in between these weekly contacts for support or to provide any additional training on the devices as required.

At the end of the run-in period, subjects will be invited to attend the research centre where the study insulin pump and CGM device will be downloaded and the data used for treatment optimisations.

11.6 Randomisation

The order of closed-loop and usual insulin pump therapy will be randomly allocated using a centrally administered web-based randomisation programme. Randomisation will take place 24 to 48 hours before the start of the first intervention arm.

The next section describes the situation when closed-loop precedes the conventional pump therapy.

11.7 Visit 5 (Closed-loop visit; within 1 week of visit 4)

Subject will arrive at the clinical research facility at an agreed time with the study team.

Urine pregnancy test will be performed on arrival.

Subjects will be provided with a training session on all aspects of the closed-loop system relevant for safe and effective use. This will include training on connection and disconnection of the closed-loop system and switching between closed-loop and usual pump therapy. Particular attention will be paid to meal bolus procedure and use of closed-loop treatment during exercise. Written step by step guidance will also be provided. Competency on the use of closed-loop system will be assessed by the

study team. Only the subjects who demonstrate competency on use of the system will be allowed to continue to the home study phase.

Closed-loop glucose control will start once the training session is completed.

Subjects will be provided with a meal at the CRC. The meal will be accompanied by meal bolus delivered from closed-loop system.

Subjects will be allowed to go home on the same day and they may walk, use public transport, or will be provided with transport according to local practice in each centre. They will be advised to continue closed-loop at home over the next one month.

During the one month home treatment period, the subject will be asked to upload /download the study insulin pump and CGM device at regular intervals (after each sensor insertion during closed loop to three weeks during open loop as given by data storage capacity of Freestyle Navigator Receiver) for storage of data. In addition, subjects can also use software provided by the manufacturer of CGM device (Abbott Diabetes Care – CoPilot Software) for visualization of CGM data during home study period.

During the one month home closed-loop treatment, the subject is allowed to drive while adhering to usual precautions and country specific rules and regulations.
Participants will be warned about hypoglycemia if they start regular exercise during study period. During the first 2 weeks of each study period subject will be advised against international travel. During rest of study period travel inside European Economic Area is allowed.

Subjects will be provided with 24 hour telephone helpline. Subjects will also be given written instructions about dealing with low and high glucose at home and when to contact study team.

Subjects will be asked to monitor finger stick glucose levels every 4 - 6 hours during waking hours. They will be required to contact the study team if they encounter any problems with closed-loop system.

11.8 Visit 6 – End of CL treatment

The subject will be invited to attend the research centre approximately 1 month after visit 5. This would be the end of one month home CL use. Insulin pump and CGM device data will be downloaded. Subject will be asked to complete a closed-loop user

evaluation questionnaires. The control algorithm device will be collected. The subject may use the study CGM and study pump during the washout period.

11.9 Washout

Minimum washout period of 2 weeks (maximum 4 weeks) must be ensured between treatment arms. Duration of the wash out period is chosen to minimise carry over effect between the two interventions. This duration was decided pragmatically based on experience of the study team as there are no published studies investigating an optimal washout period for closed-loop studies. Subject may continue to use the study CGM and study insulin pump during the washout period if he or she wishes. Otherwise subject will use usual insulin pump therapy (using own insulin pump) during this period. Then subjects will cross over to alternative intervention.

11.10 Visit 7

Subjects will collect CGM and study pump if handed back during visit 6.

11.11 Visit 8 (Control visit)

The order of Visit 5 and Visit 8 will be in random order. Visit 8 is similar to Visit 5 except that in Visit 8 there is no closed-loop insulin delivery and subjects will continue their usual insulin pump therapy with blinded CGM with self-management as appropriate. Subjects will follow usual treatment guidelines for driving, exercise, hyperglycaemia and hypoglycaemia.

11.12 Visit 9 – End of OL treatment

The subject will be invited to attend the research centre approximately 1 month after visit 8. This would be the end of one month OL treatment. Insulin pump and CGM device data will be downloaded..

12 Participant withdrawal criteria

The following withdrawal criteria will apply:

1. Subject is unable to demonstrate safe use of study insulin pump and / or CGM during run-in period as judged by the investigator
2. Subject fails to demonstrate compliance as mentioned in section 10.6 with study insulin pump and / or CGM during run-in period.
3. Subjects may terminate participation in the study at any time without necessarily giving a reason and without any personal disadvantage
4. Significant protocol violation or non-compliance

5. Decision by the investigator or the sponsor that termination is in the subject's best medical interest
6. Subject becomes pregnant during the study period
7. Allergic reaction to insulin
8. If patient cannot be contacted in 4 weeks subject will be considered lost to follow up

13Study Stopping Criteria

The study may be stopped if three consecutive participants withdraw on safety grounds.

14Support telephone line

There will be a 24-hour telephone helpline to the research team for subjects in case of any technical device or problems related to diabetes management such as hypo- or hyperglycaemia.

15Subject reimbursement

The study will provide the CGM device, insulin pump, closed loop components, related consumables, and glucose test strips. A study fee will be paid to reflect local practice. The amount paid will be specified in the participant information sheet and local ethics application form. Reasonable travel expenses will also be reimbursed. After completing the study, subjects will not keep the study devices. They will revert to their conventional insulin pump therapy.

16 Endpoints

16.1 Efficacy endpoints

16.1.1 Primary efficacy endpoint

The primary outcome is the time spent in the target glucose range from 3.9 to 10.0 mmol/l based on subcutaneous glucose monitoring (CGM).

16.1.2 Secondary efficacy endpoints

Secondary outcomes include:

1. HbA1c (phase 1 only)
2. Time spent above and below the target glucose (3.9 to 10.0 mmol/l) based on continuous subcutaneous glucose monitoring (CGM)
3. Average and standard deviation glucose levels based on continuous subcutaneous glucose monitoring
4. The time with glucose levels < 3.5 mmol/l and <2.8 mmol/l based on continuous subcutaneous glucose monitoring
5. The time with glucose levels in the significant hyperglycaemia, as based on continuous subcutaneous glucose monitoring (glucose levels > 16.7 mmol/l)
6. Low Blood Glucose Index (LBGI) based on continuous subcutaneous glucose monitoring
7. Total, basal and bolus insulin dose
8. Weekly trend in glucose control and insulin delivery
9. Number of nights when sensor glucose was below 3.5mmol/l for at least 20 minutes
10. Duration of periods when sensor glucose values was below 3.5mmol/l for at least 20 minutes
11. Time spent in the target glucose range from 3.9 to 10.0 mmol/l based on subcutaneous glucose monitoring (CGM) adjusted for sensor error during the entire home stay

CGM based endpoints will utilise unmodified recorded CGM. Additionally CGM corrected for the measurement error will be used (32). Endpoints will be calculated for 24 hour period, waking hours, and overnight.

16.2 Safety evaluation

Safety evaluation will comprise the number of episodes of hypoglycaemia, significant ketonemia (> 3.0mmol/l) as well as nature and severity of any other adverse events including SADEs and SAEs.

All subjects including those who withdraw will be included in the safety evaluation.

16.3 Utility evaluation

Utility evaluation is the frequency and duration of use of the closed-loop system at home.

17 Assessing and Reporting of Adverse Events

17.1 Definitions

17.1.1 Reportable Adverse Events

A reportable Adverse Event is any untoward medical occurrence that meets criteria for a serious adverse event or any unanticipated medical occurrence in a study subject that is study or device-related. Device deficiencies that could have led to a serious adverse device effect will also be reported.

17.1.2 Adverse Events

An adverse event (AE) is any untoward medical occurrence, unintended disease or injury, or untoward clinical signs (including abnormal laboratory findings) in a subject who has received an investigational device, whether or not related to the investigational medical device. This definition includes events related to the device under investigation or the comparator or to the study procedures. For users or other persons, this definition is restricted to events related to the investigational device.

17.1.3 Adverse Device Effect

An Adverse Device Effect (ADE) is an adverse event related to the use of an investigational medical device. This includes adverse events resulting from insufficient or inadequate instructions for use, deployment, implantation, installation, or operation, or any malfunction of the investigational medical device. This definition also includes any event resulting from use error or from intentional misuse of the device under investigation.

17.1.4 Serious Adverse Event

A serious adverse event (SAE) is an adverse event that:

- Led to a death
- Led to a serious deterioration in the health of the subject, that either resulted in:
    - a life threatening illness or injury
    - a permanent impairment of a body structure or function
    - in-patient hospitalisation or prolonged hospitalization
    - medical or surgical intervention to prevent life-threatening illness or injury or permanent impairment to a body structure or a body function
- Led to foetal distress, foetal death or a congenital abnormality or birth defect

A planned hospitalization for pre-existing condition, or a procedure required by the study protocol, without a serious deterioration in health, is not considered to be a serious adverse event.

More than one of the above criteria can be applicable to one event. Life-threatening in the definition of a serious adverse event or serious adverse reaction refers to an event in which the subject was at risk of death at the time of the event; it does not refer to an event which hypothetically might have caused death if it were more severe. Medical judgement should be exercised in deciding whether an adverse event or reaction is serious in other situations.

Important adverse events or reactions that are not immediately life-threatening or do not result in death or hospitalisation but may jeopardise the subject or may require intervention to prevent one of the other outcomes listed in the definition above, should also be considered serious.

17.1.5 Serious Adverse Device Effect

A Serious Adverse Device Effect (SADE) is an adverse device effect that has resulted in any of the consequences characteristic of a serious adverse event.

17.1.6 Unanticipated Serious Adverse Device Effect

An Unanticipated Serious Adverse Device Effect (USADE) is a serious adverse device effect which by its nature, incidence, severity or outcome has not been identified in the current version of the protocol.

This includes unanticipated procedure related serious adverse events; that is, serious adverse events occurring during the study procedure that are unrelated to any malfunction or misuse of the investigational medical device.

An Anticipated Serious Adverse Device Effect (ASADE) is a serious adverse device effect which by its nature, incidence, severity or outcome has been identified in the current protocol.

17.1.7 Device Deficiencies

A device deficiency is an inadequacy of a medical device with respect to its identity, quality, durability, reliability, safety or performance. Device deficiencies include malfunctions, user errors and inadequate labelling. A device deficiency may lead to an Adverse Device Effect or Serious Adverse Device Effect.

17.1.8 Adverse Event Intensity

Intensity	Definition
Mild	Patient is aware of signs and symptoms but they are easily tolerated
Moderate	Signs / symptoms cause sufficient discomfort to interfere with usual activities
Severe	Patient is incapable to work or perform usual activities

NB. The term “severe” is often used to describe the intensity (severity) of a specific event. This is not the same as ‘serious’, which is based on patient/event outcome or action criteria (see definition 12.1.4). For example, itching for several days may be rated as severe, but may not be clinically serious.

17.1.9 Adverse Event Causality

Intensity	Definition
Not assessable	A report suggesting an adverse event, which cannot be judged because information is insufficient or contradictory, and which cannot be supplemented or verified.
Unlikely	A clinical event, including laboratory test abnormality, with a temporal relationship, which makes a causal relationship improbable, and in which other drugs/treatments, chemicals or underlying disease(s) provide plausible explanations.
Possible	A clinical event, including laboratory test abnormality, with a reasonable time sequence to administration of the treatment/use of investigational treatment/device, but which also could be explained by concomitant diseases or other drugs/treatments or chemicals.
Probable	A clinical event, including laboratory test abnormality, with a reasonable time sequence to administration of the treatment/use of medical method/device, unlikely to be attributable to concomitant

	disease(s) or other drugs/treatments or chemicals, and which follows a clinically reasonable response on withdrawal (dechallenge). Rechallenge information is not required to fulfil this definition.
Certain	A clinical event, including laboratory test abnormality, occurring in a plausible time relationship to study treatment/use of medical method/device and which cannot be explained by concomitant disease(s), other drugs/treatments or chemicals. The response to withdrawal of the treatment (dechallenge) should be clinically plausible. The event must be unambiguous, either pharmacologically or as phenomenon, using satisfactory rechallenge procedures if necessary.

(Reference: WHO-UMC Causality Categories)

17.2 Recording and Reporting of Adverse Events, Serious Adverse Events and Device Deficiencies

17.2.1 Monitoring Period of Adverse Events

The period during which adverse events will be reported is defined as the period from the beginning of the study (obtaining informed consent) until 3 weeks after the end of the second intervention. Adverse events that continue after the subject's discontinuation or completion of the study will be followed until their medical outcome is determined or until no further change in the condition is expected. The follow up of AEs may therefore extend after the end of the clinical investigation; however no new AEs will be reported after the trial reporting period.

17.2.2 Recording and reporting of Adverse Events

Throughout the course of the study, all efforts will be made to remain alert to possible adverse events or untoward findings. The first concern will be the safety of the subject, and appropriate medical intervention will be taken. The investigator will elicit reports of adverse events from the subject at each visit and complete adverse event forms. All AEs, including those the subject reports spontaneously, those the investigators observe, and those the subject reports in response to questions will be recorded on paper or electronic AE forms at each site within seven days of discovering the event.

The study investigator will assess the relationship of any adverse event to be device-related or unrelated by determining if there is a reasonable possibility that the

adverse event may have been caused by the study device or study procedures. The individual investigator at each site will be responsible for managing all adverse events according to local protocols, and decide if reporting is required.

17.2.3 Severe Hypoglycaemia

Severe hypoglycaemia will be defined as an event requiring assistance of another person to actively administer carbohydrate, glucagon, or other resuscitative actions. These episodes may be associated with sufficient neuroglycopaenia to induce seizure or coma. If plasma glucose measurements are not available during such an event, neurological recovery attributable to the restoration of plasma glucose to normal is considered sufficient evidence that the event was induced by a low plasma glucose concentration.

Severe hypoglycaemia will be regarded as a foreseeable adverse event and an adverse event form will be completed. Severe hypoglycaemia is not necessarily a serious adverse event and hence may not require immediate reporting to the Sponsor. Non-severe hypoglycaemia will not be reported or considered an adverse event.

17.2.4 Reporting of Serious Adverse Events and Serious Adverse Device Effects

When reporting adverse events, all pertinent data protection legislation must be adhered to.

The serious adverse event report should contain the following information*:

1. Study identifier (EudraCT number if applicable)
2. Participant's unique study number
3. Date of birth
4. Event description
5. Start date of event
6. Laboratory tests used and medical interventions used to treat the SAE
7. Planned actions relating to the event, including whether the study device was discontinued
8. Statement on the patient's current state of health
9. Reason for seriousness (i.e. death, life threatening, hospitalisation, disability/incapacity or other)
10. Evaluation of causality (including grade of relatedness) with the following (more than one may apply):
    a. the investigational treatment/medical device

b. the clinical study/a study specific procedure
c. other: e. g. concomitant treatment, underlying disease

11. Reporter's name, date and signature

*In the case of incomplete information at the time of initial reporting, all appropriate information should be provided as soon as this becomes available.

The relationship of the SAE to the investigational treatment / medical device should be assessed by the investigator at site, as should the anticipated or unanticipated nature of any SAEs and SADEs.

All SAEs whether or not deemed investigational method/device related and whether anticipated or unanticipated must be reported to the Sponsor by email or fax within 24 hours (one working day) of the Investigator learning of its occurrence.

Stephen Kelleher
Cambridge University Hospitals
NHS Foundation Trust
Box 277, Addenbrooke's Hospital
Hills Road, Cambridge, CB2 0QQ, UK
Phone: +44 (0) 1223 217418
Fax: +44 (0) 1223 348494
E-mail: r&denquiries@addenbrookes.nhs.uk

A written report must follow within five working days and is to include a full description of the event and sequelae, in the format detailed on the Serious Adverse Event reporting form. If applicable, the Sponsor will notify the competent authority of all Serious Adverse Events in line with pertinent legal requirements.

The Investigator will notify the Research Ethics Committee (REC) of all Serious Adverse Events in line with pertinent legal requirements. The Investigator will inform the Sponsor about all reports sent to the REC including follow-up information and answers by the REC. The local investigator is responsible for informing other site principal investigators and the CI of all SAEs.

The regulatory authority (MHRA) will be notified of all SAEs as soon as possible within ten days of the event occurring during the study. The main REC will be notified of all unexpected and related SAEs within 15 days of the occurrence of the event.

17.2.5 Recording and Reporting of Device Deficiencies

All device deficiencies will be documented throughout the study. The investigator at each site will be responsible for managing all device deficiencies and determine and document in writing whether they could have led to a serious adverse device effect.

All device deficiencies that might have led to a serious adverse device effect(s) if: suitable action had not been taken; intervention had not been made; or if circumstances had been less fortunate, must be reported to the Sponsor as for SAEs/SADEs.

17.2.6 Healthcare Arrangements and Compensation for Adverse Events

Healthcare arrangements for subjects who suffer an adverse event as a result of participating in the study may include advice from clinical members of the study team or the patient's treating diabetes team, or use of emergency health services.

If an adverse event occurs, there are no special compensation arrangements unless this was due to the negligence of one of the clinical investigators or due to harm resulting from study protocol design. In this case subjects may have grounds for legal action for compensation. The normal national complaints mechanism will be available. In addition, any harm arising due to study design (both negligent and non-negligent) will be covered under Sponsor's insurance policy as applicable.

17.2.7 Country specific requirements:

1. UK - The Investigator will notify the ethics committee of all Serious Adverse Events in line with pertinent legal requirements. The Investigator will inform the Sponsor about all reports sent to the ethics committee including follow-up information and answers by the ethics committee. The MHRA and REC will be notified of all SUSARs occurring during the study according to the following timelines: fatal and life-threatening within 7 days of notification and non-life threatening within 15 days.
2. Germany - Any event caused by a medical device and classified as 'Serious' must be immediately reported electronically to the Competent Authority (BfArM) for further investigation according to the Ordinance on Medical Devices Vigilance (MPSV). All SAEs must be reported by using the templates and coding provided by the BfArM (BfArM.de/Medizinprodukte/Klinische Prüfung/ klinische Prüfung / Information zur Stellung eines Antrags).

A serious adverse event is every event occurring in a clinical trial subject to approval or performance evaluation subject to approval which led, might have led or might lead directly or indirectly to death or a serious deterioration in state of health of a trial subject, user or other person without considering whether the event was caused by the medical device; this also applies to serious adverse events occurring in clinical trials or performance evaluations for which an exemption from the authorisation requirement according to § 20 (1) 2 of the German Medical Device Law was granted.

The responsible person for pharmacovigilance is:
Judith Beer
ProfilInsitutfürStoffwechselforschung GmbH
Hellersbergstr. 9, D-41460 Neuss
Germany
Tel.: +49 (0) 2131 4018 145
Fax: +49 (0) 2131 4018 517
email: Judith.beer@profil.com.

3. Austria - All SAEs have to be documented by the sponsor and immediately reported according to § 42 (8) of the Austrian Medical Device Directive (StF: BGBl. Nr. 657/1996, BGBl. I Nr. 143/2009) to the competent authority (AGES) and the competent authorities of other countries within the European Union where the study is conducted. All SAEs must be reported using the templates provided by AGES.

17.3 Risks and discomforts and potential / anticipated adverse events and adverse device events/effects

17.3.1 Hypoglycaemia and hyperglycaemia

Subjects with type 1 diabetes have a pre-existing risk for hypoglycaemia and hyperglycaemia. Potential risks are:

- Risk of mild to moderate hypoglycaemia and associated symptoms such as sweating, trembling, difficulty thinking and dizziness. There is also a rare risk of severe hypoglycaemia when conscious level is altered, needing help from a third party to correct the hypoglycaemia. These risks are pre-existent in any patient with type 1 diabetes and the study objective is to develop systems to minimise these risks
- Risk of possible mild to moderate hyperglycaemia similar to the risk that a subject with type 1 diabetes experiences on a daily basis

- Risk of hyperglycaemia leading to diabetic ketoacidosis (DKA). This risk is pre-existent in any patient with type 1 diabetes.

17.3.2 Finger-prick blood glucose measurements

Finger-prick tests may produce pain and/or bruising at the site.

17.3.3 Insulin pump therapy

Subjects participating in this study are already using an insulin pump. Potential risks and adverse device effects associated with insulin pump therapy include:

- Slight discomfort at the time of insertion of the insulin delivery cannula (common)
- Slight bruising at the site of insertion (common)
- Infusion set failure leading to high glucose and need for replacement infusion set (common)
- Bleeding at insertion site (rare)
- Infection at the site of insertion (rare)
- Allergy to insulin Aspart (very rare)
- Priming mechanism failure
- Pump screen damage
- Fast battery drainage

17.3.4 Venepuncture

Potential risks of venepuncture include:

- Localised infection – an infection in the tissue around the site
- Phlebitis – inflammation of the wall of the vein
- Haematoma – an accumulation of blood within the tissues that clots to form a solid swelling

17.3.5 Continuous glucose monitoring

Potential risks and adverse device effects associated with CGM:

- Slight discomfort at the time of insertion of CGM (common)
- Slight bruising at the site of insertion (unlikely)
- Bleeding at insertion site (rare)
- Infection at the site of insertion (very rare)
- Intermittent CGM failure (common)

If a skin reaction is classified as severe (the observation is noticeable and bothersome to the subject and may indicate infection or risk of infection or potentially life-threatening allergic reaction); an adverse event form will be completed.

No serious adverse events are expected during the study.

18 Questionnaires

As part of phase 1, subjects will complete semi-structured interviews and / or questionnaires which include questions about their private attitudes, feelings and behaviour related to diabetes. It is possible that some people may find these questionnaires to be mildly upsetting. Similar questionnaires have been used in previous research and these reactions are uncommon. As part of phase 2, subjects will complete the participant experience questionnaire to document their experience using closed-loop.

19 Burdens

The study will involve up to 20 visits (phase 1) or 9 visits (phase 2), but several of these visits could be done via e-mail/ telephone. The study also includes two 6 hour visits to the clinical research facility and three months (phase 1) and one month (phase 2) home study periods.

At home, subjects will wear the closed-loop system for either 3 months period in phase 1 or 1 month in phase 2. The subject is required to carry the closed-loop system for work and when going outside home. During the night, the computer will be located on a bedside table.
The subjects will be required to perform regular finger stick measurements (4 - 6 hourly) during day time.

20 Risk analysis and residual risk associated with the investigational device

A detailed risk analysis for the closed-loop system was conducted, according to Cambridge University Hospitals NHS Foundation Trust's standard Risk Assessment Tool. The risk analysis is presented in a separate risk analysis report.

The hazard analysis has led to two hazardous situations in which the residual risk identified exceed a predefined score after all possible control measures have been applied. Risk/benefit analyses concerning these hazardous situations have been conducted by experienced and knowledgeable multidisciplinary members of the research team.

The risks/benefit analysis concluded that closed-loop is expected to reduce substantially but not to eliminate the risk of hypoglycaemia. This is supported by clinical data recorded over more than 100 nights at the clinical research facility, by data collected during home studies comprising over 900 nights and 100 days, by simulations, and is further enhanced by the requirement for a calibration check to be performed each night before closed-loop is initiated.

21Benefits

During the run-in and optimisation period, the subject will have close contact with the study team and professional pump educator and usual pump therapy settings will be optimised. This together with further education provided during the study will benefit the patient beyond the study period. CGM data provided during the study may also help patients to optimise their usual pump therapy

22Data Monitoring and Ethics Committee

An independent Data Monitoring and Ethics Committee (DMEC) will be informed of all serious adverse events and any unanticipated adverse device effects that occur during the study and will review compiled adverse event data at periodic intervals.

23 Methods and Assessments

23.1 Procedures

23.1.1 Height and weight

These will be recorded at the study initiation visit at baseline. Height will be measured in centimetres using calibrated measuring devices. Weight will be measured in kilograms using a calibrated electronic scale.

23.1.2 Continuous subcutaneous glucose monitoring

At least 10 days of CGM data will be collected before visit 8 (phase 1) and at least 7 days before visit 4 (phase 2). During the study subject may be required to upload the CGM data via internet and home computer using secure website.

23.1.3 Insulin pump data

During the study subject is required to upload insulin pump data via internet and home computer using secure website.

23.2 Questionnaires and Interviews

23.2.1 Questionnaires

During phase 1, psychosocial data will be collected using a mixed methods approach. Quantitative data will be collected using the Diabetes Technology Questionnaire and ADDQoL questionnaire. During phase 2, participants' experience using closed-loop will be documented using the participant experience questionnaire.

23.2.2 Qualitative Interview

During phase 1, qualitative data will be collected from semi-structured interviews with all participants at baseline, mid-point and study end. Interviews will explore participants' experiences, their expectations and feelings immediately prior to starting the intervention, then at the end of each phase i.e. closed loop and open loop to determine whether the technologies matched expectations, whether any difficulties occurred, what were the benefits and downsides of living with the technologies for the duration of the trial and any another aspects that have arisen as a consequence of participation. This mixed methods approach will provide a cohesive, holistic psychosocial assessment whilst minimising participant burden.

23.3 Laboratory methods

23.3.1 Baseline Sample

Blood sample will be obtained at baseline to perform various tests as described in section 10.2 of the protocol.

Blood samples for the measurement of HbA1c levels will be taken at five different time points: baseline, beginning and end of each intervention arm.

HbA1c will be measured at a local laboratory compliant with IFCC reference HbA1c method. In Cambridge HbA1c will be measured at the pathology department, Addenbrooke's Hospital using the TOSOH A1c G7 analyser (inter-assay CV 3.71% at HbA1c 5.41%; 1.7% at HbA1c 10.6%). HbA1c is will be reported in IFCC units. DCCT to IFCC conversions for key levels are shown below:

DCCT %	IFCC mmol/mol
6.0	42
6.5	48
7.0	53
7.5	59
8.0	64

23.3.2 Total Blood Loss

The total blood loss will be approximately 50 to 100 ml.

23.4 Assessment of safety

Subjects will be advised to monitor finger stick capillary blood glucose every 4-6 hours when awake. Subjects with type 1 diabetes receive structured education about treating hypo- and hyperglycaemia and these treatment principles will be re-emphasised. They will be advised to treat any finger stick capillary glucose level below 4 mmol/l with quick acting carbohydrate. Written guidelines in keeping with subjects' usual treatment guidelines will be provided for dealing with hypo- and hyperglycaemia.

23.5 Assessment of efficacy

This will be based on continuous glucose monitoring data.

24 Study Materials

24.1 Insulin

Rapid acting insulin analogues (Insulin Aspart, Insulin Lispro or Insulin Glulisine) 100 IU/ml will be used in the insulin pumps.

24.2 Study insulin pump

The subcutaneous insulin infusion pump as described in section 5.7 will be used.

24.3 Study continuous subcutaneous glucose monitor

The CGM System as described in section 5.7 will be used in this study.

24.4 Computer-based algorithm

The computer-based controller will be used. The controller has been used safely and effectively in the closed-loop studies in both children and adults with T1D **(study REC Ref. 06/Q0108/350, REC Ref. 07/H0306/116, REC Ref. 08/H0304/75, REC Ref. 08/H0308/297 and REC Ref. 09/H0306/44).**

24.5 Conventional insulin pump treatment

The conventional insulin pump treatment will be subject’s usual insulin pump therapy.

24.6 Glucometer

This is the built-in glucometer in the CGM system as described in section 5.6.

25 Data Analysis

25.1 Primary Analysis

The primary analysis will evaluate the change in the time spent in the target glucose range defined as CGM between 3.9 and 10 mmol/l.

25.2 Secondary Analysis

Secondary endpoints are listed in section 16.1.2.

25.3 Interim analysis

No interim analysis will be performed.

25.4 Statistical Methods

All analysis will be conducted on an intention to treat basis. Data from participants with or without protocol violation including dropouts and withdrawals will be included in the analysis.

The respective values obtained during the 3 months (phase 1) or 1 month (phase 2) randomised interventions contrasting the closed-loop system against the standard pump therapy combined with continuous glucose monitoring will be compared using a repeated measures regression model that accounts for period effect. Residual values from the regression model will be examined for an approximate normal distribution. If values are highly skewed a transformation or nonparametric analyses will be used. The statistical analysis will include the assessment of the period effect.

Primary analysis will be a single comparison and no attempt will be formally made to control the overall type I error rate for the secondary outcomes. A 5% significance level will be used to declare statistical significance for the primary comparison.

Severe hypoglycaemic events and ketone-positive hyperglycaemia will be tabulated in each treatment group, which will be compared using repeated measures logistic regression (generalised estimator equation). For purposes of analysis, a severe hypoglycaemic event will be defined as an event requiring assistance of another person actively to administer carbohydrate, glucagon, or other resuscitative actions. These episodes may be associated with sufficient neuroglycopaenia to induce seizure or coma. If plasma glucose measurements are not available during such an event, neurological recovery attributable to the restoration of plasma glucose to normal is considered sufficient evidence that the event was induced by a low plasma glucose concentration.

Safety data including severe hypoglycaemia events and ketone-positive hyperglycaemia will be tabulated for all subjects, including drop-outs and withdrawals, irrespective of whether CGM data are available and irrespective of whether closed-loop was operational.

25.5 Exploratory Analysis

Per protocol analysis will be conducted to explore the relationship between usage of study treatments and study outcomes. Additional analysis will include assessment of study outcomes on monthly basis, and contrasting night vs. waking hour outcomes.

25.6 Sample Size and Power Calculations

Power calculation is based on improvements in time in target. Assuming a standard deviation of 18% at baseline and average improvement of time in target of 10% - 31 subjects are required at the desired 80% power and an alpha level of 0.05 (two-tailed). If the improvement is 12% - required sample size will come down to 20. (33)

For the phase 1 study, up to 42 subjects could be recruited aiming for 30 completed subjects to allow for drop-outs. Subjects who drop out of the study during optimisation period and within the first 4 weeks of the first intervention arm will be replaced.

For the phase 2 study, up to 34 subjects could be recruited aiming for 24 completed subjects to allow for drop-outs. Subjects who drop out of the study during optimisation period and within the first 2 weeks of the first intervention arm will be replaced.

25.7 Deviations from the statistical plan

Any deviations from the original statistical plan will be recorded and agreed by the Investigators

26 Case Report Forms

The Case Report Form (CRF) is the printed, optical, or electronic document designed to record all the protocol required information to be reported to the Chief Investigator for each study participant.

CRFs will be completed in accordance with GCP and ISO 14155:2011 Guidelines. Corrections to the CRF will be performed by striking through the incorrect entry and by writing the correct value next to the data that has been crossed out; each correction will be initialled and explained (if necessary) by the Investigator or the Investigator’s authorised staff.

The electronic CRF system provides an edit feature that records the identity of the person making the change and retains a record of the before and after values of the data field(s) in question. In addition, all eCRF changes require electronic review and signoff by the investigator associated with the visit.

If any amendments to the protocol or other study documents are made, CRFs will be reviewed to determine if an amendment to these forms is also necessary.

27 Data Management

Confidentiality of subject data shall be observed at all times during the study. Personal details for each subject taking part in the research study and linking them to a unique identification number will be held locally on a study screening log in the Trial Master File at each of the investigation centres. These details will not be revealed at any other stage during the study, and all results will remain anonymous. The study identification number will be used on the case report forms and on all the blood and serum samples that are collected throughout the study. Names and addresses will not be used. Collected samples will be stored securely and locked away. Only researchers directly involved in the study will have access to the samples.

Electronic data will be stored on password-protected computers. All paper records will be kept in locked filing cabinets, in a secure office at each of the investigation centres. Only members of the research team and collaborating institutions will have

password access to the anonymised electronic data. Only members of the research teams will have access to the filing cabinet. Paper copies of the data will be stored for 15 years in line with the Data Protection Act 1998.

Direct access to the source data will be provided for monitoring, audits, REC review and regulatory authority inspections during and after the study. The fully anonymised data may be shared with third parties (EU or non-EU based) for the purposes of advancing management and treatment of diabetes.

Appropriate procedures agreed by the Chief Investigator and Clinical Principal Investigators will be put in place for data review, database cleaning and issuing and resolving data queries.

28 Study Management

28.1 Data Monitoring and Ethics Committee

An independent Data Monitoring and Ethics Committee (DMEC) will comprise a chairperson and two experts. The DMEC will be informed of all serious adverse events and any unanticipated adverse device effects/events that occur during the study. The DMEC will review compiled adverse event data at periodic intervals. The DMEC will report to the Study Management Committee any safety concerns and recommendations for suspension or early termination of the investigation.

28.2 Study Management Committee

A study management committee consisting of the Chief Investigator, Study Coordinator, and Study Data Manager will meet quarterly to discuss the operational aspects of the study. The Principal Clinical Investigators may also participate.

28.3 Study Monitoring

The Study Coordinator will ensure that the study is conducted in accordance with ICH GCP standards through site monitoring visits. A monitoring plan will be written and agreed prior to randomisation.

29 Responsibilities

29.1 Chief Investigator

The Chief Investigator (CI) is the person with overall responsibility for the research and all ethical applications will be submitted by the CI. The CI is accountable for the conduct of the study and will ensure that all study personnel are adequately qualified and informed about the protocol, any amendments to the protocol, the study treatments and procedures and their study related duties. The CI should maintain a list of appropriately qualified persons to whom he/she has delegated specified significant study-related duties.

29.2 Principal Clinical Investigators

The Principal Clinical Investigators at each investigation centre will be responsible for the day-to-day conduct of the clinical aspects of the study.

29.3 Study Coordinator

The Study Coordinator will provide day-to-day support for the sites and provide training through Principal Investigator meetings, site initiation and routine monitoring visits.

30 Ethics

The study will be conducted in accordance with the Declaration of Helsinki Ethical Principles for Medical Research involving Human Subjects (October 2000).

30.1 Independent Ethics Committees

Prior to commencement of the study, the protocol, any amendments, subject information/informed consent form, any other written information to be provided to the subject, subject recruitment procedures, current investigator CVs, and any other documents as required by the Ethics Committee will be submitted. Written approval will be obtained from relevant Ethics Committee prior to the commencement of the study.

30.2 Informed consent of study subjects

In obtaining and documenting informed consent, the investigator will comply with the applicable regulatory requirements and will adhere to Good Clinical Practice Guidelines and to the ethical principles that have their origin in the Declaration of Helsinki. Prior to the start of the study, the Investigator will obtain favourable ethical opinion of the written informed consent form and any other written information to be provided to subjects.

Subjects will be given full verbal and written information regarding the objective and procedures of the study and the possible risks involved. They will be informed about their right to withdraw from the study at any time. The signed informed consent forms will be photocopied, originals filed in the Investigator’s Site File, and a copy placed in the patient’s notes and a copy given to the subjects.

31 Timetable

In phase 1, inclusion of the first subject is planned to take place in September 2013. The expected completion of the last subject is Q3 2014 and the planned completion of the Clinical Study Report is November 2014.

In phase 2, inclusion of the first subject is planned to take place in March 2016. The expected completion of the last subject is Q4 2016 and the planned completion of the Clinical Study Report is March 2017.

32 Deviations from Protocol

Deviations from the protocol should not occur. If deviations do occur, they will be documented, stating the reason and the date, the action taken, and the impact for the subject and for the study. The documentation will be kept in the Investigator’s Site File.

33 Reports and Publications

Data will be published in internationally peer-reviewed scientific journals; members of the investigator group will all be co-authors.

34 Retention of Study Documentation

Subject notes must be kept for the maximum of time period as permitted by relevant institution. Other source documents and the Investigator's Site File must be retained for at least 15 years. The Principal Investigator will archive the documentation pertaining to the study in an archive after completion or discontinuation of the file.

35 Indemnity Statements

Indemnity for any harm arising from the conduct of research will be provided according to local arrangements in respective centre.

12. Cambridge, UK - National Health Service indemnity cover will apply for any claims arising from management and conduct of research. Any liability arising from study design will be covered by the clinical trial insurance policy organised by the University of Cambridge.
13. Neuss, Germany – Profil Institute for Metabolic Research, Neuss, Germany, will provide insurance for the subjects according to local requirements.
14. Graz, Austria – Subjects will be insured according to Medical Device Law § 47 (StF: BGBl. Nr. 657/1996, BGBl. I Nr. 143/2009)

36 Appendices and Tables

36.1 Appendix 1

Participating Centres in conducting AP@home clinical studies:

1. University of Cambridge and Cambridge University Hospitals NHS Foundation Trust
2. Academic Medical Centre, Amsterdam, Netherlands
3. Profil Institute for Metabolic Research, Neuss, Germany
4. Medical University,of Graz, Graz Austria
5. University of Padova, Padova, Italy
6. Centre Hospitalier Regional Universitaire, Montpellier, France

36.2 Appendix 2

Reduced time in hypoglycaemia when insulin infusion is driven by two closed-loop algorithms in patients with Type 1 diabetes; a randomized clinical trial

E.M. Renard[1], J.H. DeVries[2], R. Hovorka[3], W. Doll[4], L. Heinemann[5], C. Cobelli[6], L. Magni[7], A. Farret[1], Y.M. Luijf[2], L. Leelarathna[3], J. Mader[4], C. Benesch[5], D. Bruttomesso[6], F. Di Palma[7], M. Nodale[3];

[1]Endocrinology, Diabetes, Nutrition, Montpellier University Hospital, Montpellier Cedex 5, France, [2]Department of internal medicine, Academic Medical Center at the University of Amsterdam, Amsterdam, Netherlands, [3]Institute of Metabolic Science, University of Cambridge, Cambridge, UK, [4]University of Graz, Graz, Austria, [5]Profil Institute, Neuss, Germany, [6]University of Padova, Padova, Italy, [7]University of Pavia, Pavia, Italy.

BACKGROUND AND AIMS: Closed loop algorithms built for model predictive control (MPC) aim to keep blood glucose close to normal in patients with Type 1 diabetes by the tuning of insulin delivery based on continuous glucose monitoring (CGM) and prediction of glucose levels.

MATERIALS AND METHODS:Blood glucose was controlled for 23 h in 47 patients in six centers by one of two MPC algorithms developed either at the University of Pavia with a Safety Supervision Module from UVA & UCSB (iAP) or at Cambridge University (CAM), or by the patients themselves in Open Loop (OL) mode, in a randomized three-way cross-over design during three clinical research center admissions including three meals and an exercise bout. CGM data were provided by the Dexcom Seven Plus (Dexcom Inc, San Diego, CA, USA) and fed to the algorithm while insulin was administered by Omnipod Insulin Pump (Insulet Corp, Bedford, MA, USA), either automatically with the Artificial Pancreas System (UCSB/Sansum, CA, USA) (three centers) or manually (three centers). For glucose reference measurements the YSI glucose analyzer was used. Intention-to-treat (ITT) and per-protocol (PP) analyses were done using a general linear model for repeated measures. For the PP analysis, 0.4% (OL), 13% (iAP) and 17% (CAM) of time was discarded, because of CGM or insulin pump malfunction or operator mistakes.

RESULTS: Percentage of time spent in euglycaemia (3.9-8, postmeal 10 mmol/L) was similar in closed and open loop modes, both in ITT and PP analyses: 62.6 and 62.8 for OL, 59.2 and 59.3 for iAP, 58.3 and 59.6 for CAM. While mean glucose level (mmol/L) was significantly lower in open loop mode, both in ITT and PP analyses: 7.19 and 7.18 for OL, 8.15 and 8.27 for iAP, 8.26 and 8.15 for CAM (overall P=0.001), % time spent in hypoglycaemia (<3.9 mmol/L) was almost threefold reduced during closed loop, both in ITT and PP analyses: 6.4 and 6.3 for OL, 2.1 and 0.9 for iAP, 2.0 and 0.0 for CAM (overall P=0.001) with less % time ≤2.8 mmol/L in ITT and PP analyses (overall P= 0.038 and 0.017). There were no significant differences in any of these outcomes between the two closed loop algorithms. The feasibility

of automated management of the closed loop systems was documented in the 3 investigation centers that tested it.

CONCLUSION: From the largest and first head-to-head closed loop study performed so far, we conclude that both CAM and iAP MPC algorithms allow safer control of blood glucose than patient self management, even during meal and exercise challenges, with less hypoglycaemia at the expense of a higher mean glucose. In future trials, tuning of the algorithms will aim at increased % time in target and lower mean glucose level while keeping the reduction of hypoglycaemia.

36.3 Appendix 3

Automated overnight closed-loop glucose control in young children with type 1 diabetes

Elleri D, Allen JM, Nodale M, Wilinska ME, Mangat JS, Larsen AM, Acerini CL, Dunger DB, Hovorka R.

Metabolic Research Laboratories, Institute of Metabolic Science, University of Cambridge, Cambridge, United Kingdom.

BACKGROUND:
We evaluated the effectiveness of automated overnight closed-loop (AOCL) insulin delivery and the influence of timing of initiation on glucose control overnight in young children with type 1 diabetes (T1D).

METHODS:
Eight children with T1D (four boys, four girls) (mean ± SD: 9.4 ± 2.7 years old; body mass index, 18.3 ± 2.3 kg/m(2); duration of diabetes, 3.9 ± 2.5 years; total daily insulin dose, 0.7 ± 0.1 U/kg/day; glycosylated hemoglobin, 7.9 ± 0.9%) were studied in a clinical research facility on two separate occasions. Subjects had a meal at 18:00 (77 ± 8 g of carbohydrate [CHO]) and snack at 21:00 (21 ± 6 g of CHO), both accompanied by a prandial insulin bolus. In random order, AOCL was started at 18:00 or 21:00 h and ran until 08:00 h the next day. Subcutaneous continuous glucose monitoring data were fed automatically into the model predictive control algorithm. Calculated subcutaneous insulin infusion rates were sent wirelessly to an insulin pump. Plasma glucose was measured to assess closed-loop performance.

RESULTS:
No rescue CHOs were administered. Time spent with plasma glucose in the target range from 3.9 to 8.0 mmol/L was 50.7% (29.0%, 72.2%), and it did not differ on the two occasions: median (interquartile range), 42% (18%, 64%) versus 58% (32%, 79%) (P = 0.161). Time when plasma glucose was above 8.0 mmol/L (42% [25%, 82%] vs. 29% [14%, 64%], P = 0.093), time below 3.9 mmol/L (0% [0%, 11%] vs. 8% [0%, 17%], P = 0.500), low blood glucose index (0.1 [0.0, 2.5] vs. 1.7 [0.4, 3.3], P = 0.380), plasma glucose at the start of AOCL (12.5 ± 2.7 vs. 11.6 ± 4.2 mmol/L, P = 0.562), and mean overnight plasma glucose (8.3 ± 2.1 vs. 7.5 ± 2.2 mmol/L, P = 0.246) were also similar.

CONCLUSIONS:
AOCL is feasible in young children with T1D. Comparable results were obtained when closed-loop was initiated at 18:00 or 21:00 h.

Schematic representation of automated closed-loop system used in this study is shown in Figure 3.

Figure 3: Schematic representation of the automated closed-loop system used in reference (21) and above appendix 3.

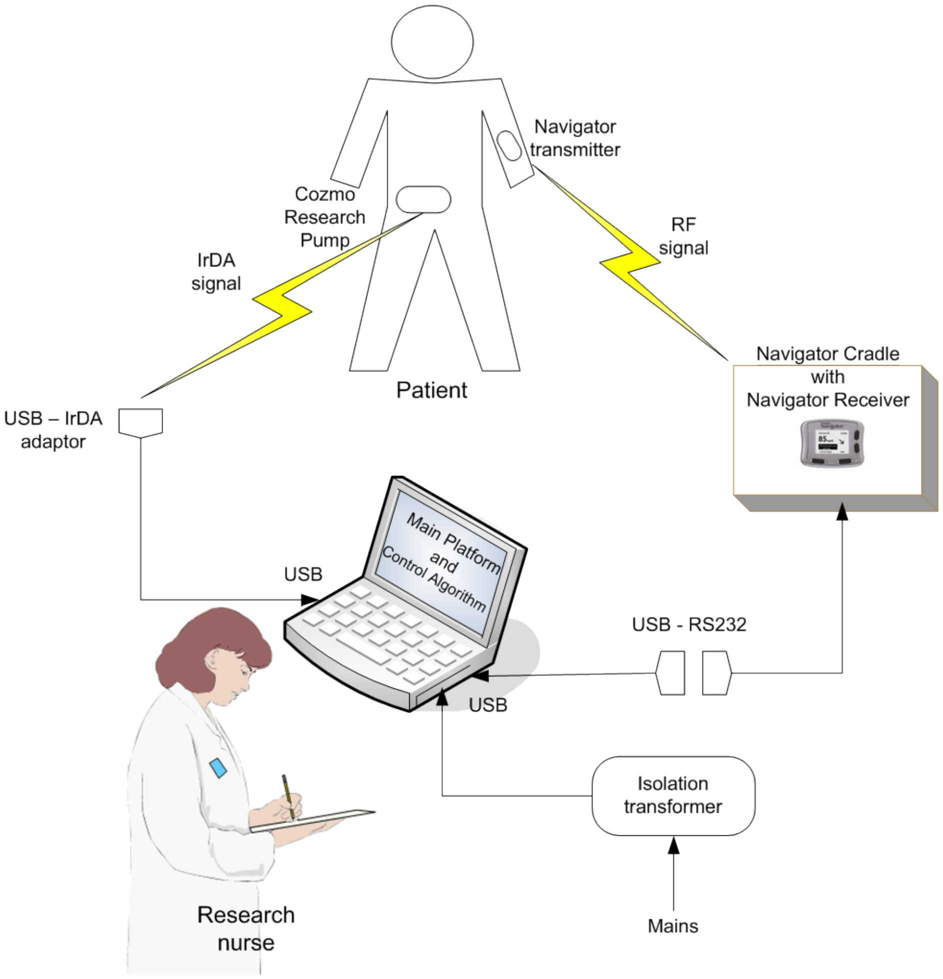

Figure 4: FlorenceD automated closed-loop system

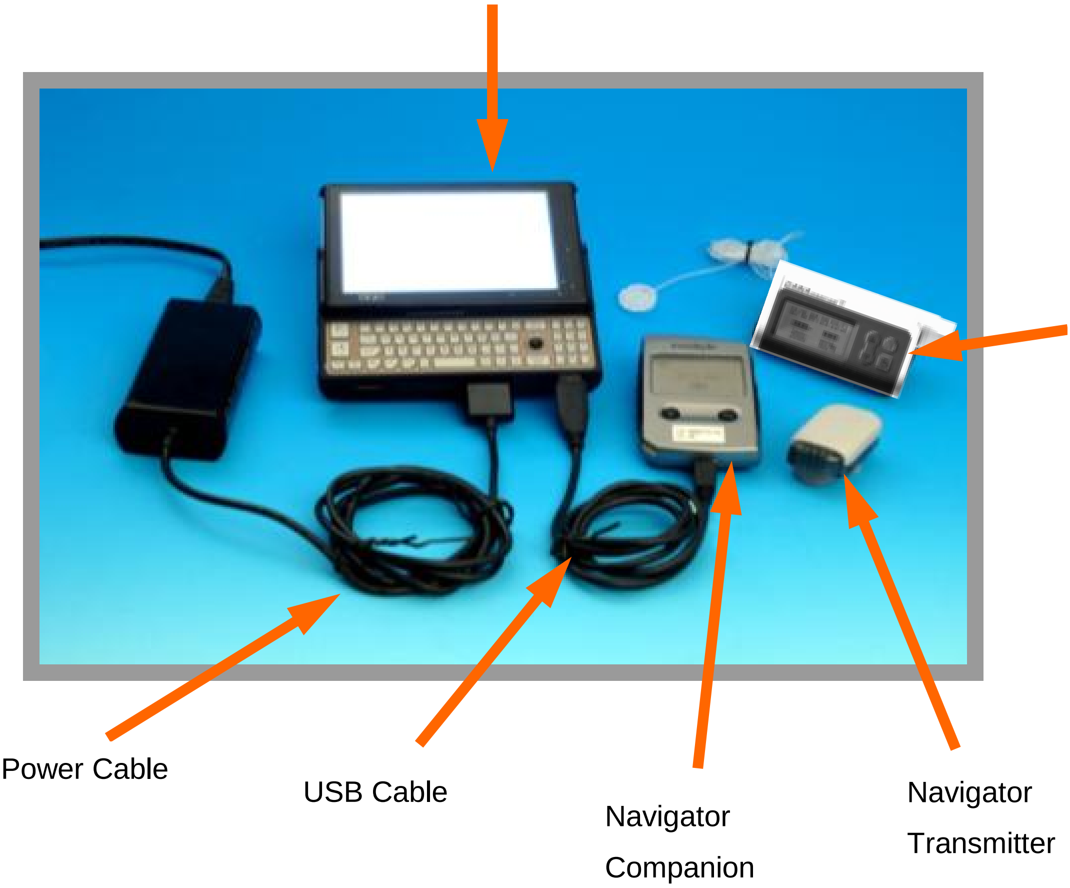

36.4 Appendix 4

Automated Overnight Closed-loop Insulin Delivery in Young People with Type 1 Diabetes: Evaluating Portable Ambulatory Prototype

D Elleri, JM Allen, M Biagioni, K Kumareswaran, L Leelarathna, K Caldwell, M Nodale, ME Wilinska, CL Acerini; DB Dunger; R Hovorka

Metabolic Research Laboratories, Institute of Metabolic Science, Cambridge, UK
Department of Paediatrics, University of Cambridge, Cambridge, UK

AIMS:
We evaluated ambulatory portable prototype overnight automated closed-loop (CL) system after early and later closed-loop initiation.

METHODS:
A randomised cross-over study compared automated overnight glucose control started at the time of an evening-meal or at bed-time. Eight young people with type 1 diabetes (T1D) on insulin pump therapy (M 4; age 14.3±1.7years; A1C 8.2±1.3%; mean±SD) were studied on two occasions at a clinical research facility. Standardised self-selected evening meal (70±11gCHO) and snack (22±4gCHO) accompanied by prandial insulin boluses were given at 18:00 at 21:00, respectively. In random order, automated CL started at 18:00 or 21:00 and run until 8:00 the next day. Basal insulin delivery was automatically adjusted by a model predictive control algorithm based on real-time continuous glucose monitor readings.

RESULTS:
Overnight plasma glucose levels (between 21:00 and 08:00) were within the target range (3.9-8.0mmol/L) for 82 (59,98)% of time when CL started at 18:00 and 64 (48,70)% when started at 21:00 [median (IQR), p=0.04]. Time spent within 3.9-10mmol/L [87(77,100) vs 81(74,88)%, p=NS], above 10mmol/l [8(0,17) vs 13(3,23)%, p=NS] or below 3.9mmol/L [0(0,7) vs 0(0,8)%, p=NS] did not differ between the two occasions. Mean overnight glucose (6.7±0.8 vs 7.6±0.7mmol/L) was also similar. Insulin infusion rates were higher when CL started at 21:00 (0.9±0.4 vs 0.8±0.4U/h, p=0.01, paired t-test). No interruptions to closed-loop delivery were observed.

CONCLUSIONS:
Automated closed-loop delivery can be applied reliably and safely to control glucose levels overnight in young people with T1D. Tighter glucose levels may be achieved with an earlier time of CL initiation.

`

36.5 Appendix 5

Home overnight closed-loop insulin delivery over three weeks: Randomised controlled trial in adolescents with type 1 diabetes

R. Hovorka[1,2], D. Elleri[1,2], J.M. Allen[1,2], M. Nodale[1], H. Thabit, K. Kumareswaran[1],L. Leelarathna[1], K. Caldwell[1], E. O'Sullivan[1], H.R. Murphy[1], A. Gulati, J. Mangat, C. Kollman[3], P. Calhoun[3], M.E. Wilinska[1,2], C.L. Acerini[2], D.B. Dunger[1,2]

[1]Institute of Metabolic Science, Metabolic Research Laboratories, University of Cambridge, Cambridge, UK

[2]Department of Paediatrics, University of Cambridge, Cambridge, UK
[3]The Jaeb Center for Health Research, Tampa, FL, USA

Free-living studies are needed to assess benefits of closed-loop (CL) insulin delivery to build on encouraging observations taken during controlled clinical centre conditions. We evaluated home use of overnight CL, initially in seven pump-treated adolescents with type 1 diabetes [M 4; age 15.5(2.1)yrs; A1C 7.9%(0.8); BMI 23.3(2.2)kg/m^2, duration of diabetes 6.1(3.8)yrs; total daily dose 0.9(0.2)U/kg/day; mean(SD)] who, after training on study devices and passing competency assessment, underwent two 3-week periods of sensor augmented pump therapy (FreeStyle Navigator Transmitter and Combined Controller, Abbott Diabetes Care; Dana R Diabecare pump, Sooil) separated by 1 to 3 week washout. During one 3-week period, randomly assigned, overnight insulin delivery was automatically modulated by adaptive model predictive controller running on an ultraportable laptop. Apart from overnight CL, diabetes management was identical during the two study periods. Overall, CL was operational for at least 4 hrs on 112 (76%) nights; sensor data were available for at least 4 hrs on 107 (73%) non-CL nights (night defined between 11pm and 7am). CL increased the time glucose was in target range between 3.9 and 8.0mmol/L at night [58% (40, 77) vs. 42% (13, 69), CL vs. non-CL; P<0.001, median (IQR)]. Mean overnight glucose was reduced during CL [7.9 (2.1) vs. 8.7 (3.1)mmol/L; P = 0.006, mean(SD)] as was time > 8.0mmol/L [35.5% (17.7, 57.8) vs. 47.1% (17.7, 87.2), P = 0.006]. Time < 3.9mmmol/L was comparable [1.4% (0.3, 4.4) vs. 0.5% (0.0, 9.7), P = 0.24] but the percentage of nights with glucose < 3.5mmol/l for at least 20 min was halved (7% vs. 19%, P = 0.01). No serious adverse event or severe hypoglycaemia was observed. We conclude that overnight closed-loop insulin delivery is feasible in home setting providing benefits similar to those observed under controlled conditions in adolescents with type 1 diabetes.

36.6 Appendix 6

Diabetes Technology Questionnaire

Part 1: Impact and Satisfaction

Thank you for giving us your time and effort in taking part in this study. Your opinions about using diabetes technology are very valuable to us and we hope that you can now help us learn how this has affected your daily life with diabetes. Below you will see some statements about different kinds of diabetes treatments that include using different diabetes devices such as a blood glucose meter, insulin pump, continuous glucose monitor or closed loop insulin delivery system.

Please tick the box below that lists the diabetes devices you are using now as part of treatment. If you aren't sure, ask the diabetes nurse to help you.

☐ Glucose Meter(s) and daily injections of insulin

☐ Glucose Meter(s) and daily use of an insulin pump

☐ Glucose Meter(s) and a Continuous Glucose Sensor (Companion, Navigator, DexCom, Paradigm or Guardian-RT) and daily injections of insulin

☐ Glucose Meter(s) and a Continuous Glucose Sensor (Companion, Navigator, DexCom, Paradigm or Guardian-RT) and daily use of an insulin pump

☐ Glucose Meter(s) and a Continuous Glucose Sensor (Companion, Navigator, DexCom, Paradigm or Guardian-RT) and night-time use of a "closed-loop" insulin delivery system that adjusts insulin doses automatically

☐ Glucose Meter(s) and a Continuous Glucose Sensor (Companion, Navigator, DexCom, Paradigm or Guardian-RT) and 24 hour use of a "closed loop" insulin delivery system that adjusts insulin doses automatically

Now we'd like to ask you some questions about the treatment approach that you selected above. We've listed below some parts of living with diabetes that might be made better or worse by your use of diabetes devices. For each of these, please circle the number that best

describes how much of a problem it is now and then circle the number that best describes how it has changed for you **compared to the treatment received before you entered this study**.

Before the study begins, please complete the pink columns only. On all other occasions, please complete the pink and blue columns.

	Is this a problem now?					How has it changed compared to your treatment before the study?				
	Very much	Quite a lot	A bit	Not much	Not at all	Much worse	A little worse	Same	A little better	Much better
1. Worry or fear about high blood sugar	1	2	3	4	5	1	2	3	4	5
2. Effort to keep low blood sugar from happening	1	2	3	4	5	1	2	3	4	5
3. Worry or fear about low blood sugar during sleep	1	2	3	4	5	1	2	3	4	5
4. Feeling different from others	1	2	3	4	5	1	2	3	4	5
5. Amount of time spent thinking about diabetes	1	2	3	4	5	1	2	3	4	5
6. Not knowing how eating affects blood sugar	1	2	3	4	5	1	2	3	4	5

	Is this a problem now?					How has it changed compared to your treatment before the study?				
	Very much	Quite a lot	A bit	Not much	Not at all	Much worse	A little worse	Same	A little better	Much better
7. Amount of time and effort needed for diabetes from my family or me	1	2	3	4	5	1	2	3	4	5
8. Worry or fear about long term health	1	2	3	4	5	1	2	3	4	5
9. Worry or fear about daytime low blood sugar	1	2	3	4	5	1	2	3	4	5
10. Effort to keep high blood sugar from happening	1	2	3	4	5	1	2	3	4	5
11. Pain or discomfort from finger sticks or sensors	1	2	3	4	5	1	2	3	4	5
12. Pain or discomfort from insulin injections or pump sets	1	2	3	4	5	1	2	3	4	5
13. Family arguments or worries about diabetes	1	2	3	4	5	1	2	3	4	5
14. Trouble sleeping well	1	2	3	4	5	1	2	3	4	5
15. Strictness of the meal plan	1	2	3	4	5	1	2	3	4	5
16. Coping with work or school along with diabetes	1	2	3	4	5	1	2	3	4	5

	Is this a problem now?					How has it changed compared to your treatment before the study?				
	Very much	Quite a lot	A bit	Not much	Not at all	Much worse	A little worse	Same	A little better	Much better
17. Taking part in sports, exercise, or playing despite diabetes	1	2	3	4	5	1	2	3	4	5
18. Knowing how much insulin to take	1	2	3	4	5	1	2	3	4	5
19. Keeping up with friends or peers who don't have diabetes	1	2	3	4	5	1	2	3	4	5
20. Reacting to all of the blood sugar results that I get	1	2	3	4	5	1	2	3	4	5
21. Dealing with others who ask about diabetes	1	2	3	4	5	1	2	3	4	5
22. My amount of responsibility for taking care of diabetes	1	2	3	4	5	1	2	3	4	5
23. Being sure that pre-meal insulin covers the amount of carbohydrates eaten	1	2	3	4	5	1	2	3	4	5
24. Getting the right amount of insulin when meals are skipped or delayed	1	2	3	4	5	1	2	3	4	5
25. Reacting to all of the alarms from diabetes devices	1	2	3	4	5	1	2	3	4	5
26. Getting the right amount of insulin on sick days	1	2	3	4	5	1	2	3	4	5

	Is this a problem now?					How has it changed compared to your treatment before the study?				
	Very much	Quite a lot	A bit	Not much	Not at all	Much worse	A little worse	Same	A little better	Much better
27. Feeling that diabetes devices run my life	1	2	3	4	5	1	2	3	4	5
28. Getting the right amount of insulin after exercising more than usual	1	2	3	4	5	1	2	3	4	5
29. Coping with carrying and using several devices	1	2	3	4	5	1	2	3	4	5
30. Looking different because of diabetes and using devices	1	2	3	4	5	1	2	3	4	5

Diabetes Technology Questionnaire

Part 2: User Friendliness

Next, we'd like to ask your opinions about the "user-friendliness" of the different diabetes devices that you have been using. For each type of device you have been using, write in the model of the device and then rate that device by circling the number that best matches your opinion about each aspect of using that device.

**Blood glucose meter (make/model):**						**Other comments about using this blood glucose meter**
	**Terrible**	**Poor**	**Fair**	**Good**	**Excellent**	
31. Size, weight, appearance and fashion issues	1	2	3	4	5	
32. Ease of start-up, calibration, etc.	1	2	3	4	5	
33. Battery life and ease of replacement	1	2	3	4	5	
34. Variety and flexibility of functions	1	2	3	4	5	
35. Instructions, manual and technical support	1	2	3	4	5	
36. Screen information and reports	1	2	3	4	5	

**Blood glucose meter (make/model):**						**Other comments about using this blood glucose meter**
	**Terrible**	**Poor**	**Fair**	**Good**	**Excellent**	
37. Alarm functions	1	2	3	4	5	
38. Use during sports, exercise, bathing	1	2	3	4	5	
39. Accuracy and reliability of performance	1	2	3	4	5	

**Insulin pump (make/model):**						**Other comments about using this insulin pump**
	**Terrible**	**Poor**	**Fair**	**Good**	**Excellent**	
40. Size, weight, appearance and fashion issues	1	2	3	4	5	
41. Ease of start-up, calibration, etc.	1	2	3	4	5	
42. Battery life and ease of replacement	1	2	3	4	5	

Insulin pump (make/model):						Other comments about using this insulin pump
	Terrible	Poor	Fair	Good	Excellent	
43. Variety and flexibility of functions	1	2	3	4	5	
44. Instructions, manual and technical support	1	2	3	4	5	
45. Screen information and reports	1	2	3	4	5	
46. Alarm functions	1	2	3	4	5	
47. Use during sports, exercise, bathing	1	2	3	4	5	
48. Accuracy and reliability of performance	1	2	3	4	5	

Continuous glucose monitor (make/model):						Other comments about using this continuous glucose monitor
	Terrible	Poor	Fair	Good	Excellent	
49. Size, weight, appearance and fashion issues	1	2	3	4	5	
50. Ease of start-up, calibration, etc.	1	2	3	4	5	
51. Battery life and ease of replacement	1	2	3	4	5	
52. Variety and flexibility of functions	1	2	3	4	5	
53. Instructions, manual and technical support	1	2	3	4	5	
54. Screen information and reports	1	2	3	4	5	
55. Alarm functions	1	2	3	4	5	
56. Use during sports, exercise, bathing	1	2	3	4	5	

Continuous glucose monitor (make/model):						Other comments about using this continuous glucose monitor
	Terrible	Poor	Fair	Good	Excellent	
57. Accuracy and reliability of performance	1	2	3	4	5	

"Closed-Loop" insulin delivery system:						Other comments about using this "closed-loop insulin delivery system
	Terrible	Poor	Fair	Good	Excellent	
58. Size, weight, appearance and fashion issues	1	2	3	4	5	
59. Ease of start-up, calibration, etc.	1	2	3	4	5	
60. Battery life and ease of replacement	1	2	3	4	5	

"Closed-Loop" insulin delivery system:						Other comments about using this "closed-loop insulin delivery system
	Terrible	Poor	Fair	Good	Excellent	
61. Variety and flexibility of functions	1	2	3	4	5	
62. Instructions, manual and technical support	1	2	3	4	5	
63. Screen information and reports	1	2	3	4	5	
64. Alarm functions	1	2	3	4	5	
65. Use during sports, exercise, bathing	1	2	3	4	5	
66. Accuracy and reliability of performance	1	2	3	4	5	

36.7 Appendix 7 – ADDQoL Questionnaire

ADDQoL

This questionnaire asks about your quality of life – in other words how good or bad you feel your life to be.

Please put an "X" in the box that best indicates your response for each item.

What we would like to know is how you feel about your life now.

I) In general, my present quality of life is:

☐	☐	☐	☐	☐	☐	☐
excellent	very good	good	neither good nor bad	bad	very bad	extremely bad

Now we would like to know how your quality of life is affected by your diabetes, its management and any complications you may have.

II) If I did *not* have diabetes, my quality of life would be:

☐	☐	☐	☐	☐
very much better	much better	a little better	the same	worse

For information only

NOT FOR USE: for review & study administration. Ref HPR 1398
ADDQoL-19 © Prof Clare Bradley: 24.2.94. Standard UK English (rev. 1.3.06)
Health Psychology Research, Dept of Psychology, Royal Holloway, University of London, Egham, Surrey, TW20 0EX, UK

Please respond to the more specific statements on the following pages. For each aspect of life described, you will find two parts:

For Part (a):	put an "X" in one box to show how diabetes affects this aspect of your life;
For Part (b):	put an "X" in one box to show how important this aspect of your life is to your quality of life.

For information only

1	(a)	**If I did *not* have diabetes, I would enjoy my leisure activities:**				
		☐ very much more	☐ much more	☐ a little more	☐ the same	☐ less
	(b)	**My leisure activities are:**				
		☐ very important	☐ important	☐ somewhat important	☐ not at all important	

2		**Are you currently working, looking for work or would you like to work?**				
		**Yes** ☐ If ***yes***, complete (a) and (b).				
		**No** ☐ If ***no***, go straight to 3a.				
	(a)	**If I did *not* have diabetes, my working life would be:**				
		☐ very much better	☐ much better	☐ a little better	☐ the same	☐ worse
	(b)	**For me, having a working life is:**				
		☐ very important	☐ important	☐ somewhat important	☐ not at all important	

3	(a)	**If I did *not* have diabetes, local or long distance journeys would be:**				
		☐ very much easier	☐ much easier	☐ a little easier	☐ the same	☐ more difficult
	(b)	**For me, local or long distance journeys are:**				
		☐ very important	☐ important	☐ somewhat important	☐ not at all important	

NOT FOR USE: for review & study administration. Ref HPR 1398
ADDQoL-19 © Prof Clare Bradley: 24.2.94. Standard UK English (rev. 1.3.06)
Health Psychology Research, Dept of Psychology, Royal Holloway, University of London, Egham, Surrey, TW20 0EX, UK

**4**	**Do you ever go on holiday or want to go on holiday?**<br>**Yes** ☐ If ***yes***, complete (a) and (b).<br>**No** ☐ If ***no***, go straight to 5a.
**(a)**	**If I did *not* have diabetes, my holidays would be:**<br>☐ very much better ☐ much better ☐ a little better ☐ the same ☐ worse
**(b)**	**For me, holidays are:**<br>☐ very important ☐ important ☐ somewhat important ☐ not at all important

**5 (a)**	**If I did *not* have diabetes, physically I could do:**<br>☐ very much more ☐ much more ☐ a little more ☐ the same ☐ less
**(b)**	**For me, how much I can do physically is:**<br>☐ very important ☐ important ☐ somewhat important ☐ not at all important

**6**	**Do you have any family / relatives?**<br>**Yes** ☐ If ***yes***, complete (a) and [illegible].<br>**No** ☐ If ***no***, go straight to 7[illegible]
**(a)**	**If I did *not* have diabetes, my family life would be**<br>☐ very much better ☐ much better ☐ a little better ☐ the same ☐ worse
**(b)**	**My family life is:**<br>☐ very important ☐ important ☐ somewhat important ☐ not at all important

**7 (a)**	**[illegible] did *not* have diabetes, my friendships and social life would be:**<br>☐ very much better ☐ much better ☐ a little better ☐ the same ☐ worse
**(b)**	**My friendships and social life are:**<br>☐ very important ☐ important ☐ somewhat important ☐ not at all important

For information only

NOT FOR USE: for review & study administration. Ref HPR 1398
ADDQoL-19 © Prof Clare Bradley: 24.2.94. Standard UK English (rev. 1.3.06)
Health Psychology Research, Dept of Psychology, Royal Holloway, University of London, Egham, Surrey, TW20 0EX, UK

8	Do you have or would you like to have a close personal relationship (e.g. husband / wife, partner)?<br>Yes ☐ If ***yes***, complete **(a)** and **(b)**.<br>No ☐ If ***no***, go straight to 9.
**(a)**	**If I did *not* have diabetes, my closest personal relationship would be:**<br>☐ very much better ☐ much better ☐ a little better ☐ the same ☐ worse
**(b)**	**For me, having a close personal relationship is:**<br>☐ very important ☐ important ☐ somewhat important ☐ not at all important

9	Do you have or would you like to have a sex life?<br>Yes ☐ If ***yes***, complete **(a)** and **(b)**.<br>No ☐ If ***no***, go straight to **10a**.
**(a)**	**If I did *not* have diabetes, my sex life would be:**<br>☐ very much better ☐ much better ☐ a little better ☐ the same ☐ worse
**(b)**	**For me, having a sex life is:**<br>☐ very important ☐ important ☐ somewhat important ☐ not at all important

**10 (a)**	**If I did *not* have diabetes, my physical appearance would be:**<br>☐ very much better ☐ much better ☐ a little better ☐ the same ☐ worse
**(b)**	**My physical appearance is:**<br>☐ very important ☐ important ☐ somewhat important ☐ not at all important

**11 (a)**	**If I did *not* have diabetes, my self-confidence would be:**<br>☐ very much better ☐ much better ☐ a little better ☐ the same ☐ worse
**(b)**	**My self-confidence is:**<br>☐ very important ☐ important ☐ somewhat important ☐ not at all important

For information only

NOT FOR USE: for review & study administration. Ref HPR 1398
ADDQoL-19 © Prof Clare Bradley: 24.2.94. Standard UK English (rev. 1.3.06)
Health Psychology Research, Dept of Psychology, Royal Holloway, University of London, Egham, Surrey, TW20 0EX, UK

12 (a) If I did _not_ have diabetes, my motivation would be:

☐ very much better ☐ much better ☐ a little better ☐ the same ☐ worse

(b) My motivation is:

☐ very important ☐ important ☐ somewhat important ☐ not at all important

13 (a) If I did _not_ have diabetes, the way people in general react to me would be:

☐ very much better ☐ much better ☐ a little better ☐ the same ☐ worse

(b) The way people in general react to me is:

☐ very important ☐ important ☐ somewhat important ☐ not at all important

14 (a) If I did _not_ have diabetes, my feelings about the future (e.g. worries, hopes) would be:

☐ very much better ☐ much better ☐ a little better ☐ the same ☐ worse

(b) My feelings about the future are:

☐ very important ☐ important ☐ somewhat important ☐ not at all important

15 (a) If I did _not_ have diabetes, my financial situation would be:

☐ very much better ☐ much better ☐ a little better ☐ the same ☐ worse

(b) My financial situation is:

☐ very important ☐ important ☐ somewhat important ☐ not at all important

16 (a) If I did _not_ have diabetes, my living conditions would be:

☐ very much better ☐ much better ☐ a little better ☐ the same ☐ worse

(b) My living conditions are:

☐ very important ☐ important ☐ somewhat important ☐ not at all important

For information only

NOT FOR USE: for review & study administration. Ref HPR 1398
ADDQoL-19 © Prof Clare Bradley: 24.2.94. Standard UK English (rev. 1.3.06)
Health Psychology Research, Dept of Psychology, Royal Holloway, University of London, Egham, Surrey, TW20 0EX, UK

17 (a)	**If I did *not* have diabetes, I would have to depend on others when I do not want to:**				
	☐ very much less	☐ much less	☐ a little less	☐ the same	☐ more
(b)	**For me, not having to depend on others is:**				
	☐ very important	☐ important	☐ somewhat important	☐ not at all important	

18 (a)	**If I did *not* have diabetes, my freedom to eat as I wish would be:**				
	☐ very much greater	☐ much greater	☐ a little greater	☐ the same	☐ less
(b)	**My freedom to eat as I wish is:**				
	☐ very important	☐ important	☐ somewhat important	☐ not at all important	

19 (a)	**If I did *not* have diabetes, my freedom to drink as I wish (e.g. fruit juice, alcohol, sweetened hot and cold drinks) would be:**				
	☐ very much greater	☐ much greater	☐ a little greater	☐ the same	☐ less
(b)	**My freedom to drink as I wish is:**				
	☐ very important	☐ important	☐ somewhat important	☐ not at all important	

If there are any other ways in which diabetes, its management and any complications affect your quality of life, please say what they are below:

Thank you for completing this questionnaire.

NOT FOR USE: for review & study administration. Ref HPR 1398
ADDQoL-19 © Prof Clare Bradley: 24.2.94. Standard UK English (rev. 1.3.06)
Health Psychology Research, Dept of Psychology, Royal Holloway, University of London, Egham, Surrey, TW20 0EX, UK

For information only

36.8 Appendix 8 – Competency Assessment forms

36.8.1 CGM Competency Assessment Form

The following skills should be demonstrated by subjects at the end of the training session on the use of the CGM. Members of the research team will ascertain a subject's ability in each of the skills required. Further training will be provided if necessary.

		Skill mastered by participant	Skill understood but requires practice	Requires more training
1.	Inserting a new sensor	☐	☐	☐
2.	Performing a calibration	☐	☐	☐
3.	Awareness of calibration schedule	☐	☐	☐
4.	Checking the life of Navigator II Receiver on the screen	☐	☐	☐
5.	Reconnecting the Receiver and the Transmitter when needed	☐	☐	☐
6.	Remembering to recharge the Translator / Receiver overnight	☐	☐	☐
7.	Understanding of Navigator Receiver screen icons (e.g. calibration, loss of connection, arrows) and how to deal with them	☐	☐	☐
8.	Checking how much sensor life is left	☐	☐	☐
9.	Setting the alarms on the Navigator II	☐	☐	☐
10.	Day and night alarms & Muting alarms	☐	☐	☐
11.	Understanding the reason why it is important to keep “data loss” alarm and “system” alarm on	☐	☐	☐
12.	Use of CoPilot Software	☐	☐	☐
13.	Use of CGM to optimise treatment	☐	☐	☐

36.8.2 Study pump Competency Assessment Form

The following skills should be demonstrated by subjects at the end of the training session on the use of the DANA insulin pump. Members of the research team will ascertain a subject's ability in each of the skills required. Further training will be provided if necessary.

		Skill mastered by participant	Skill understood but requires practice	Requires more training
1.	Putting new batteries in the DANA-R insulin pump when battery indicator down to 2 bars	☐	☐	☐
2.	Setting date and time on the pump screen	☐	☐	☐
3.	Filling the reservoir (using DANA EASY Setter) and changing the infusion set	☐	☐	☐
4.	Priming the infusion set before you attach the pump's tubing to your body using PRIME menu only, and editing the PRIME screen appropriately	☐	☐	☐
5.	Setting the basal infusion rates pattern	☐	☐	☐
6.	Viewing, editing, adding or deleting your basal programs	☐	☐	☐
7.	Setting a temporary basal rate	☐	☐	☐
8.	Reviewing time elapsed and time remaining of temporary basal, and cancelling temporary basal	☐	☐	☐
9.	Understanding the difference between "bolus" and "bolus calculator" functions and when to use them	☐	☐	☐
10.	Using the bolus calculator and performing an insulin bolus	☐	☐	☐
11.	Understanding importance of giving all boluses via pump (<u>not syringes/pen</u>)	☐	☐	☐
12.	Programming the "Correction Factor" (CF), "Carbohydrate-to-Insulin Ratio" (CIR) and "Ideal Blood Glucose" (IDEAL) on your pump	☐	☐	☐
13.	Choosing and using different bolus types, ie step/extended/dual bolus	☐	☐	☐
14	Reviewing at Extended Bolus Status screen: time elapsed, time left, insulin amount to be delivered and amount of insulin delivered so far	☐	☐	☐
15.	Stopping a bolus delivery of insulin as it is occurring	☐	☐	☐
16.	Checking the insulin on board on the pump	☐	☐	☐

	Skill mastered by participant	Skill understood but requires practice	Requires more training
17. Setting alerts/alarms on the pump	☐	☐	☐
18. Changing the alarm and alert settings (how your pump communicates the alerts to you) on the pump	☐	☐	☐

36.8.3 Closed-loop Competency Assessment Form

The following skills should be demonstrated by subjects at the end of the training session on the use of the Closed-loop. Members of the research team will ascertain a subject's ability in each of the skills required. Further training will be provided if necessary.

		Skill mastered by participant	Skill understood but requires practice	Requires more training
1.	Understanding the concept of closed-loop delivery	☐	☐	☐
2.	Switching smartphone ON/OFF, passcodes & checking battery level	☐	☐	☐
3.	Awareness of importance of connecting smartphone and translator to mains power with leads provided overnight	☐	☐	☐
4.	Being aware of keeping the smartphone, Translator with Navigator II and DANA-R insulin pump as close together as possible to ensure connectivity	☐	☐	☐
5.	Starting & stopping closed-loop	☐	☐	☐
6.	Understanding of closed-loop delivering extended boluses with zero basal	☐	☐	☐
7.	Understanding information on DANA-R pump screen during closed-loop delivery [i.e. usual basal rate stopped (0.00U/h), extended bolus active	☐	☐	☐
8.	Understanding the process of delivering a meal bolus during closed-loop using smartphone (Bolus Tab)	☐	☐	☐
9.	Understanding the process of delivering a correction bolus during closed-loop intervention using smartphone (Bolus Tab)	☐	☐	☐
10.	Understanding the message log (Messages tab)	☐	☐	☐
11.	Understanding meaning of common error messages	☐	☐	☐
12.	Understanding the graph display on the smartphone	☐	☐	☐
13.	Understanding how to check alarms audio	☐	☐	☐
14.	Understanding that any on-going extended boluses will be stopped when active closed-loop starts	☐	☐	☐

15.	Understanding the importance of calibration checks	☐	☐	☐
16.	Understanding the importance of alarms being active on the Navigator II during closed-loop	☐	☐	☐
17.	Awareness of hypoglycaemia and hyperglycaemia treatment protocols	☐	☐	☐
18.	Awareness of sick day rules during usual treatment and during closed-loop treatment	☐	☐	☐
19.	Awareness of the risk of hypoglycaemia during and after exercise and strategies to minimise the risk	☐	☐	☐
20	Agree to limit alcohol intake ≤ 2 units a day	☐	☐	☐
21	Awareness of contact details of study team and 24 hour help-line	☐	☐	☐
22	Data uploading procedure	☐	☐	☐

37References

1. International Diabetes Federation, The Diabetes Atlas, Fourth Edition. Brussels:2009.
2. Group DP: Incidence and trends of childhood Type 1 diabetes worldwide 1990-1999. Diabet Med 2006;23:857-866
3. Patterson CC, Dahquist GG, Gyurus E, Green A, Soltesz G, Grp ES: Incidence trends for childhood type 1 diabetes in Europe during 1989-2003 and predicted new cases 2005-20: a multicentre prospective registration study. Lancet 2009;373:2027-2033
4. Royal College of Paediatrics and Child Health. Growing up with Diabetes: children and young people with diabetes in England. Available at http://www.rcpch.ac.uk/news/first-national-survey-finds-23000-children-diabetes-england-06-april-2009.
5. The effect of intensive treatment of diabetes on the development and progression of long-term complications in insulin-dependent diabetes mellitus. The Diabetes Control and Complications Trial Research Group. N Engl J Med 1993;329:977-986
6. Cryer PE: The barrier of hypoglycemia in diabetes. Diabetes 2008;57:3169-3176
7. Cryer PE: Hypoglycemia in type 1 diabetes mellitus. Endocrinol Metab Clin North Am 2010;39:641-654
8. Cryer PE, Axelrod L, Grossman AB, Heller SR, Montori VM, Seaquist ER, Service FJ: Evaluation and Management of Adult Hypoglycemic Disorders: An Endocrine Society Clinical Practice Guideline. Journal of Clinical Endocrinology & Metabolism 2009;94:709-728
9. Buckingham B, Beck R, Tamborlane W, Xing D, Kollman C, Fiallo-Scharer R, Mauras N, Ruedy K, Tansey M, Weinzimer S, Wysocki T, Group DRiCNDS: Continuous glucose monitoring in children with type 1 diabetes. J Pediatr 2007;151:388-393, 393.e381-382
10. Bode B, Schwartz S, Stubbs H, Block J: Glycemic characteristics in continuously monitored patients with type 1 and type 2 diabetes: normative values. Diabetes Care 2005;28:2361-2366
11. Cryer PE: Current concepts: Diverse causes of hypoglycemia-associated autonomic failure in diabetes. New England Journal of Medicine 2004;350:2272-2279
12. Cryer PE: Mechanisms of hypoglycemia-associated autonomic failure and its component syndromes in diabetes. Diabetes 2005;54:3592-3601
13. Wild D, von Maltzahn R, Brohan E, Christensen T, Clauson P, Gonder-Frederick L: A critical review of the literature on fear of hypoglycemia in diabetes: Implications for diabetes management and patient education. Patient Education and Counseling 2007;68:10-15
14. Deis D, Bolinder J, Riveline JP, Battelino T, Bosi E, Tubiana-Rufi N, Kerr D, Phillip M: Improved glycemic control in poorly controlled patients with type 1 diabetes using real-time continuous glucose monitoring. Diabetes Care 2006;29:2730-2732
15. Tamborlane WV, Beck RW, Bode BW, Buckingham B, Chase HP, Clemons R, Fiallo-Scharer R, Fox LA, Gilliam LK, Hirsch IB, Huang ES, Kollman C, Kowalski AJ, Laffel L, Lawrence JM, Lee J, Mauras N, O'Grady M, Ruedy KJ, Tansey M, Tsalikian E, Weinzimer S, Wilson DM, Wolpert H, Wysocki T, Xing DY, Juvenile Diabet Res Fdn C: Continuous glucose monitoring and intensive treatment of type 1 diabetes. New England Journal of Medicine 2008;359:1464-U1465
16. Kordonouri O, Pankowska E, Rami B, Kapellen T, Coutant R, Hartmann R, Lange K, Knip M, Danne T: Sensor-augmented pump therapy from the diagnosis of childhood type 1 diabetes: results of the Paediatric Onset Study (ONSET) after 12 months of treatment. Diabetologia 2010;
17. Hirsch IB, Abelseth J, Bode BW, Fischer JS, Kaufman FR, Mastrototaro J, Parkin CG, Wolpert HA, Buckingham BA: Sensor-augmented insulin pump therapy: Results of the first randomized treat-to-target study. Diabetes Technology & Therapeutics 2008;10:377-383
18. Hovorka R: Continuous glucose monitoring and closed-loop systems. Diabetic Medicine 2006;23:1-12
19. Hovorka R, Kumareswaran K, Harris J, Allen JM, Elleri D, Xing D, Kollman C, Nodale M, Murphy HR, Dunger DB, Amiel SA, Heller SR, Wilinska ME, Evans ML: Overnight closed loop insulin delivery (artificial pancreas) in adults with type 1 diabetes: crossover randomised controlled studies. BMJ 2011;342:d1855
20. Hovorka R, Allen JM, Elleri D, Chassin LJ, Harris J, Xing DY, Kollman C, Hovorka T, Larsen AMF, Nodale M, De Palma A, Wilinska ME, Acerini CL, Dunger DB: Manual closed-loop insulin delivery in children and adolescents with type 1 diabetes: a phase 2 randomised crossover trial. Lancet 2010;375:743-751

21. Elleri D, Allen JM, Nodale M, Wilinska ME, Mangat JS, Larsen AM, Acerini CL, Dunger DB, Hovorka R: Automated overnight closed-loop glucose control in young children with type 1 diabetes. Diabetes Technol Ther 2011;13:419-424
22. Kumareswaran K, Elleri D, Allen JM, Harris J, Xing D, Kollman C, Nodale M, Murphy HR, Amiel SA, Heller SR, Wilinska ME, Acerini CL, Evans ML, Dunger DB, Hovorka R: Meta-analysis of overnight closed-loop randomized studies in children and adults with type 1 diabetes: the Cambridge cohort. J Diabetes Sci Technol 2011;5:1352-1362
23. Murphy HR, Elleri D, Allen JM, Harris J, Simmons D, Rayman G, Temple R, Dunger DB, Haidar A, Nodale M, Wilinska ME, Hovorka R: Closed-loop insulin delivery during pregnancy complicated by type 1 diabetes. Diabetes Care 2011;34:406-411
24. Murphy HR, Kumareswaran K, Elleri D, Allen JM, Caldwell K, Biagioni M, Simmons D, Dunger DB, Nodale M, Wilinska ME, Amiel SA, Hovorka R: Safety and Efficacy of 24-H Closed-Loop Insulin Delivery in Well-Controlled Pregnant Women With Type 1 Diabetes: A randomized crossover case series. Diabetes Care 2011;
25. Elleri D, Acerini CL, Allen JM, Hayes J, Pesterfield C, Wilinska ME, Dunger DB, Hovorka R: Parental attitudes towards overnight closed-loop glucose control in children with type 1 diabetes. Diabetes Technol Ther 2010;12:35-39
26. Elleri D, Allen JM, Nodale M, Wilinska ME, Acerini CL, Dunger DB, Hovorka R: Suspended insulin infusion during overnight closed-loop glucose control in children and adolescents with Type 1 diabetes. Diabet Med 2010;27:480-484
27. Wilinska ME, Budiman ES, Taub MB, Elleri D, Allen JM, Acerini CL, Dunger DB, Hovorka R: Overnight closed-loop insulin delivery with model predictive control: assessment of hypoglycemia and hyperglycemia risk using simulation studies. J Diabetes Sci Technol 2009;3:1109-1120
28. Elleri D, Allen JM, Kumareswaran K, Leelarathna L, Nodale M, Caldwell K, Cheng P, Kollman C, Haidar A, Murphy HR, Wilinska ME, Acerini CL, Dunger DB, Hovorka R: Closed-Loop Basal Insulin Delivery Over 36 Hours in Adolescents With Type 1 Diabetes: Randomized clinical trial. Diabetes Care 2013;36:838-844
29. DeVries J, Avogaro A, Benesch C, Bruttomesso D, Caldwell K, Cobelli C, Doll W, Del Favero S, Heinemann L, Hovorka R, Leelarathna L, Luijf Y, Mader J, Magni L, Nodale M, Place J, Renard E, Toffanin C, On behalf of AP@home Consortium: Comparison of Two Closed Loop Algorithms With Open Loop Control in Type 1 Diabetes. Diabetes 2012;61 (Suppl. 1):A60
30. Elleri D, Allen JM, Biagioni M, Kumareswaran K, Leelarathna L, Caldwell K, Nodale M, Wilinska ME, Acerini CL, Dunger DB, Hovorka R: Evaluation of a portable ambulatory prototype for automated overnight closed-loop insulin delivery in young people with type 1 diabetes. Pediatr Diabetes 2012;13:449-453
31. Seaquist ER, Anderson J, Childs B, Cryer P, Dagogo-Jack S, Fish L, Heller SR, Rodriguez H, Rosenzweig J, Vigersky R: Hypoglycemia and diabetes: a report of a workgroup of the American Diabetes Association and the Endocrine Society. Diabetes Care 2013;36:1384-1395
32. Nodale M, Haidar A, Wilinska ME, Hovorka R: The use of continuous glucose monitoring (CGM) to evaluate performance of closed-loop insulin delivery systems. Diabetes 2011;60:A64
33. Norman G, Monteiro S, Salama S: Sample size calculations: should the emperor's clothes be off the peg or made to measure? BMJ 2012;345:e5278
